# Highly Active Halogen Bonding and Chalcogen Bonding Chloride Transporters with Non‐Protonophoric Activity

**DOI:** 10.1002/chem.202101681

**Published:** 2021-06-14

**Authors:** Laura E. Bickerton, Andrew Docker, Alistair J. Sterling, Heike Kuhn, Fernanda Duarte, Paul D. Beer, Matthew J. Langton

**Affiliations:** ^1^ Department of Chemistry Chemistry Research Laboratory University of Oxford Mansfield Road Oxford OX1 3TA UK

**Keywords:** anions, chalcogen bonding, halogen bonding, ionophores, membranes

## Abstract

Synthetic anion transporters show much promise as potential anti‐cancer agents and therapeutics for diseases associated with mis‐regulation of protein anion channels. In such applications high activity and anion selectivity are crucial to overcome competing proton or hydroxide transport which dissipates cellular pH gradients. Here, highly active bidentate halogen bonding and chalcogen bonding anion carriers based on electron deficient iodo‐ and telluromethyl−triazole derivatives are reported. Anion transport experiments in lipid bilayer vesicles reveal record nanomolar chloride transport activity for the bidentate halogen bonding anion carrier, and remarkably high chloride over proton/hydroxide selectivity for the chalcogen bonding anionophore. Computational studies provide further insight into the role of sigma‐hole mediated anion recognition and desolvation at the membrane interface. Comparison with hydrogen bonding analogues demonstrates the importance of employing sigma‐hole donor motifs in synthetic anionophores for achieving both high transport activity and selectivity.

## Graphical abstract

**Achieving highly selective** chloride over proton or hydroxide transport is crucial for the development of supramolecular anionophores for medical applications, where competing dissipation of pH gradients is undesired. Halogen and chalcogen bonding anionophores with record nanomolar activity are reported, and high selectivity for chloride over hydroxide is demonstrated to be achievable using these sigma‐hole interactions.

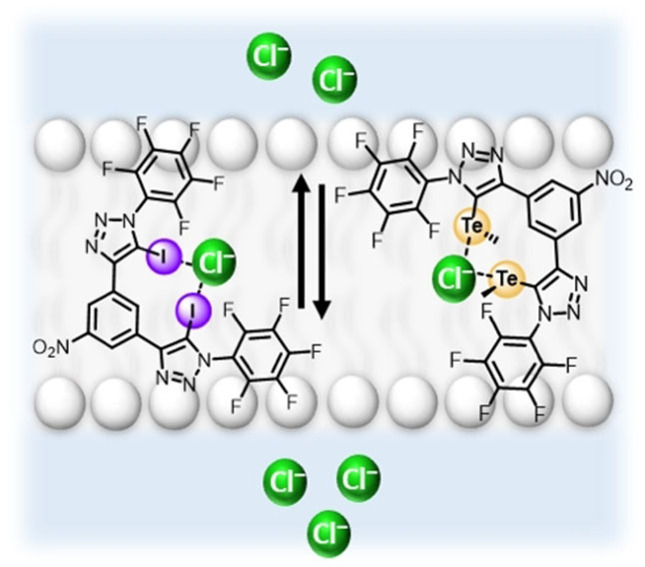

## Introduction

Nature is capable of exquisitely selective and highly efficient transmembrane ion transport using membrane‐spanning protein ion channels and pumps. Synthetic anion carriers have recently gained significant interest as tools for biomedical research and as therapeutics for channelopathies associated with anion‐channel dysfunction, such as cystic fibrosis and Best disease.[^1^, ^2^, ^3^, ^4^, ^5^, ^6^, ^7^, ^8^] These synthetic anionophores invariably employ acidic hydrogen bond donors to mediate anion recognition and transport. However, in recent years, non‐covalent sigma‐hole interactions, including halogen bonding (XB) and chalcogen bonding (ChB), have emerged as versatile intermolecular interactions for a variety of applications in supramolecular chemistry.[^9^, ^10^, ^11^, ^12^, ^13^] In the area of transmembrane anion transport, halogen bonding,[^14^, ^15^, ^16^, ^17^, ^18^, ^19^] and more recently chalcogen^[20]^ and pnictogen[^21^, ^22^, ^23^] bonding transporters represent effective alternatives to hydrogen bonding systems.

Whilst enormous progress has been made in the development of synthetic anion carriers, achieving both high selectivity *and* activity is challenging. High selectivity for chloride over proton/hydroxide (Cl^−^>H^+^/OH^−^) is particularly desirable because transmembrane pH gradients are essential for cellular function.^[24]^ Unintended dissipation of pH gradients by anionophores, such as those developed as therapies for channelopathies,^[25]^ may lead to toxicity. Indeed, many anionophores, such as those based on the naturally occurring H^+^/Cl^−^ symporter prodigiosin,[^26^, ^27^] are known to neutralise acidic organelles and uncouple ATPase proton pumps, and are therefore studied as potential anti‐cancer therapeutics.[^28^, ^29^, ^30^] Beyond applications in medicine, non‐protonophoric chloride transporters may also find utility as an anion equivalent to the potassium transporter valinomycin, which does not mediate H^+^/OH^−^ transport and finds numerous applications in physiological research.^[31]^ Recently, Gale, Davis and co‐workers highlighted that a key challenge to overcome in order to develop a highly selective Cl^−^>H^+^/OH^−^ transporter, is that employing acidic hydrogen bond donors – which are necessary to afford strong anion binding and high transport activity – will significantly suppress or completely nullify chloride selectivity.^[32]^ Common hydrogen bonding anionophores based on simple thiourea and squaramide derivatives exhibit little to no Cl^−^>H^+^/OH^−^ selectivity. Increasing the degree of anion encapsulation has been shown to improve the selectivity,^[32]^ such as within macrocyclic cholaphane derivatives,^[33]^ but at some expense to synthetic accessibility.

**Figure 1:**
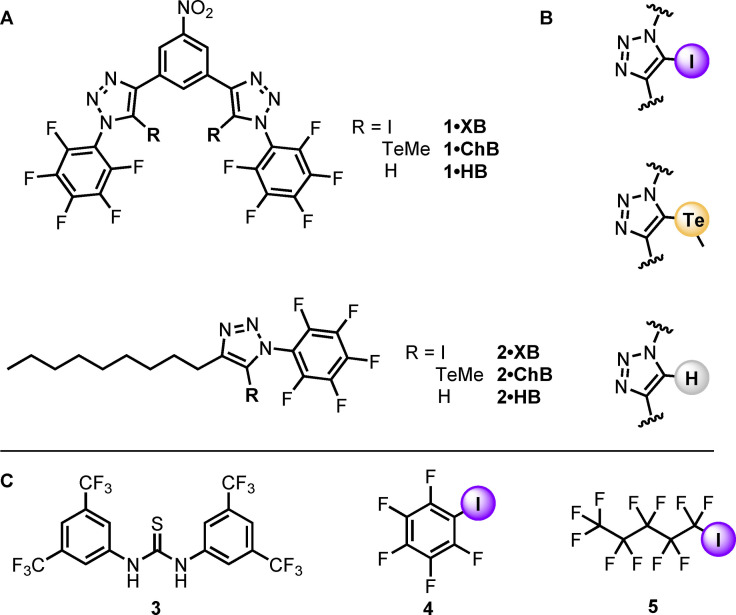
(A) Bidentate halogen, chalcogen and hydrogen bonding chloride transporters, **1 ⋅ XB**, **1 ⋅ ChB**, and **1 ⋅ HB**, and monodentate transporters **2 ⋅ HB**, **2 ⋅ XB**, **2 ⋅ ChB**. (B) Halogen, chalogen and hydrogen bonding triazole donors. (C) Control hydrogen and halogen bonding transporters.

Herein we report electron deficient bidentate iodo‐ and telluromethyl‐triazole derivatives that employ halogen bonding and chalcogen bonding intermolecular interactions to mediate highly effective transmembrane anion transport (Figure 1).The bidentate XB anionophore **1 ⋅ XB** exhibits record nanomolar activity, 3 orders of magnitude more active than the most active XB transporters reported to date.^[18]^ Importantly, remarkable Cl^−^>H^+^/OH^−^ selectivity is achieved by employing chalcogen bonding interactions in **1 ⋅ ChB**, whilst maintaining excellent anion transport activity. Comparison with the proto‐triazole hydrogen bonding analogues, and representative polar NH donor anionophores, highlights the superior capabilities of halogen bonding and chalcogen bonding anionophores for developing highly selective *and* active anion transporters.

## Results and Discussion

### Design and synthesis of XB, ChB and HB anionophores

Recently, we reported that simple acyclic, monodentate iodotriazole derivatives are potent anion transporters, outperforming the archetypal XB donors iodoperfluorobenzene **4** and iodoperfluorohexane **5**.^[18]^ Analysis of the anion transport data and computational studies revealed that multiple triazole XB donors were required per anion to effectively mediate transmembrane anion transport. To improve anion transport activity, we therefore sought to incorporate two halogen bond or chalcogen bond donors to afford bidentate anion carrier scaffolds.

Building on our previous work exploiting 3,5‐bis‐iodo‐ and 3,5‐bis‐telluromethyl‐triazole motifs for anion recognition,[^34^, ^35^, ^36^, ^37^, ^38^] including in aqueous solution,[^39^, ^40^] we incorporated this bidentate sigma‐hole donor structural framework into a neutral, lipophilic carrier design, to generate halogen‐, chalcogen‐ and hydrogen‐bonding anionophores, **1 ⋅ XB**, **1 ⋅ ChB** and **1 ⋅ HB**, respectively (Figure 1A). Analogous monodentate triazoles **2 ⋅ XB**, **2 ⋅ ChB** and **2 ⋅ HB** were also prepared for means of comparison. The anion carriers were designed such that log *P*∼5 in each case, which is within the optimum lipophilicity range for transport efficiency.^[41]^ Furthermore, in contrast to previous studies,^[21]^ the structural similarity of **1 ⋅ XB**, **1 ⋅ ChB** and **1 ⋅ HB** facilitates a more direct comparison between HB, XB and ChB intermolecular interactions for anion transport applications.

**Scheme 1:**
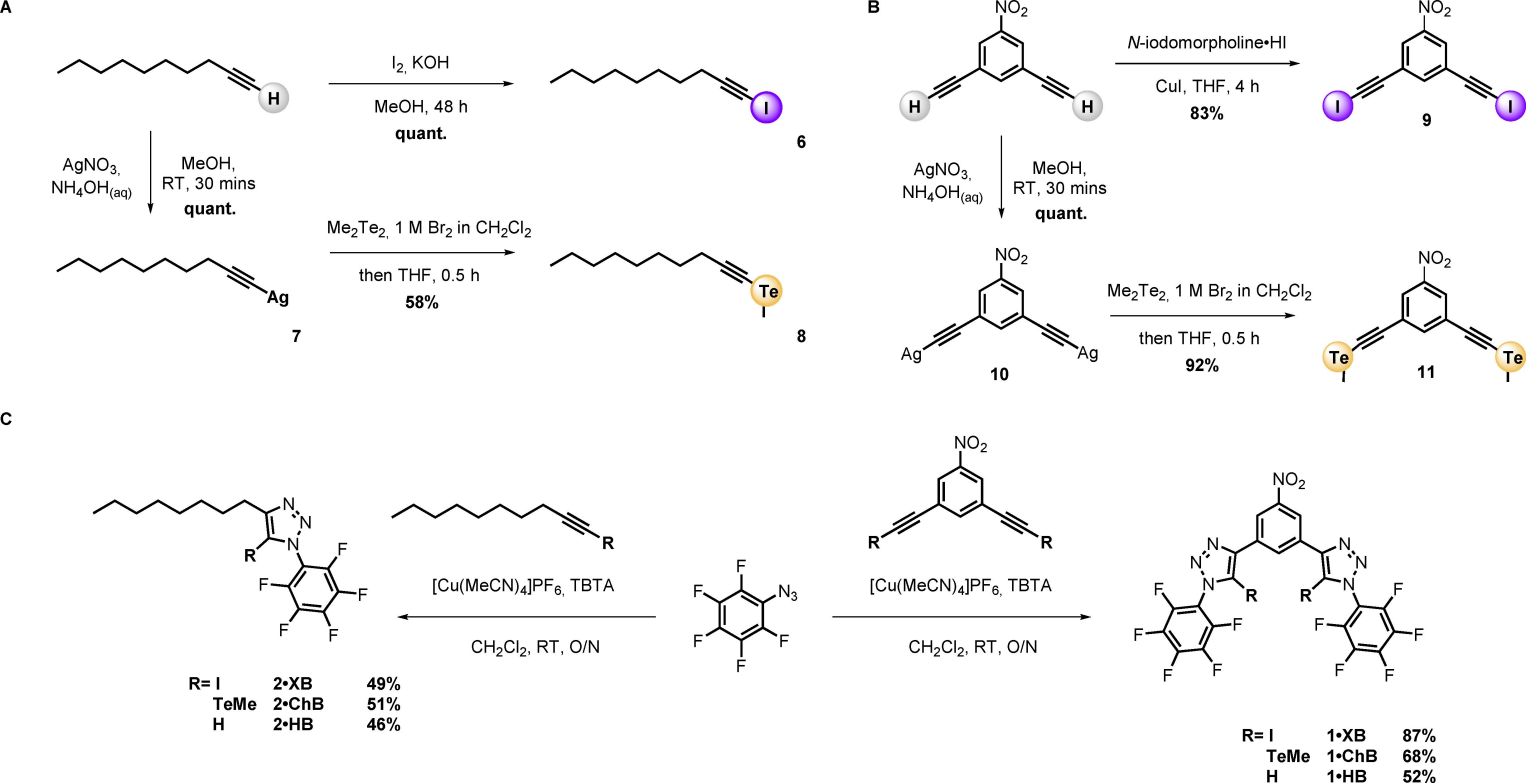
Synthesis of mono‐ and bi‐dentate halogen, chalcogen and hydrogen bonding anionophores. Preparation of monodentate (A) and bidentate (B) alkyne precursors. (C) Preparation of triazole anionophores via CuAAC click reactions

The target XB, ChB and HB transporters were prepared via a copper‐catalyzed azide−alkyne cycloaddition (CuAAC) reaction between the appropriately functionalised alkyne precursors and azido‐pentafluorobenzene. The prerequisite iodo‐ and telluromethyl‐functionalised alkyne precursors were prepared from 1‐decyne and 1,3‐diethynylnitrobenzene according to Scheme 1A. Treatment of 1‐decyne with KOH in methanol and subsequent reaction with elemental iodine afforded iodoalkyne **6** quantitatively. Reaction of 1,3‐diethynylnitrobenzene with *N*‐iodomorpholine hydroiodide in the presence of catalytic copper (I) iodide in anhydrous THF solution afforded the target bis‐iodoalkyne **9** in 83 % yield (Scheme 1B). Reaction of 1‐decyne and 1,3‐diethynylnitrobenzene with freshly ground AgNO_3_ in methanol in the presence of NH_4_OH_(aq)_ gave the required silver acetylide intermediates (**7** and **10**) which were subsequently reacted with a freshly generated solution of methyl tellurium bromide to afford **8** and **11** in 58 % and 92 % yield respectively. The alkyne precursors were subjected to CuAAC reactions with azido‐pentafluorobenzene in the presence of catalytic [Cu(MeCN)_4_]PF_6_ and Cu(I) stabilising ligand tris(benzyltriazolyl) amine (TBTA), affording the bidentate and monodentate anionophores in yields ranging from 46–87 % (Scheme 1C, see the Supporting Information for further synthetic details and characterisation data).

### Solid‐state structure determination

**Figure 2:**
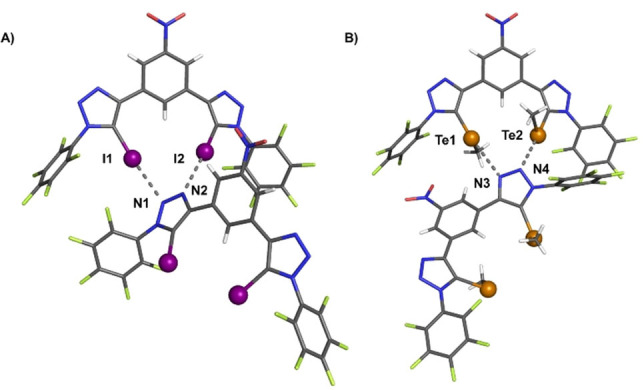
X‐ray crystallographic structures of (**A**) **1 ⋅ XB**, (**B**) **1 ⋅ ChB**. XB and ChB interactions shown as dashed lines. Grey=carbon, blue=nitrogen, white=hydrogen, red=oxygen, green=fluorine, purple=iodine, orange=tellurium. Solvent molecules omitted for clarity.

Insight into the potency of the sigma‐hole donors was provided by solid‐state characterisation of **1 ⋅ XB** and **1 ⋅ ChB**.Crystals of **1 ⋅ XB** and **1 ⋅ ChB** grown by slow evaporation of chloroform solutions were suitable for X‐ray structural analysis. The structures show the sigma‐hole donors participating in intermolecular N⋅⋅⋅I XB or N⋅⋅⋅Te ChB interactions (Figure 2, and summarised in Table S2). Of particular note is the significant 84 % and 83 % contraction in the van der Waals radii for I1⋅⋅⋅N1 and Te1⋅⋅⋅N3, respectively, indicative of intermolecular XB and ChB sigma hole interactions in the solid state.

### Anion recognition properties

^1^H NMR chloride binding titration experiments with the bidentate and monodentate carriers were conducted in acetone‐*d*_6_ with tetrabutylammonium chloride. The data was fitted to a 1 : 1 binding isotherm (see the Supporting Information) and the stoichiometric 1 : 1 association constants determined using the Bindfit[^42^, ^43^] program (Table 1, entry 1). The data revealed an overall chloride affinity trend of **XB**>**ChB**>**HB** for the bidentate and monodentate receptors. Indeed, the binding of chloride by XB receptor **1 ⋅ XB** was too strong to be determined in acetone‐d_6_ (*K*_a_ >10^5^ M^−1^), requiring the addition of 2.5 % D_2_O in order to quantify binding and revealing a forty‐fold enhancement over **1 ⋅ ChB** (Table 1, line 2). For each carrier, cooperative bidentate chloride binding between both triazole donor atoms significantly enhanced the binding affinity relative to the monodentate analogues.

**Table 1:** Chloride binding, transport, and selectivity data.

		Transporter						
entry		**1 ⋅ HB**	**1 ⋅ XB**	**1 ⋅ ChB**	**2 ⋅ HB**	**2 ⋅ XB**	**2 ⋅ ChB**	**3** ^[i]^
1	*K*_1_^Cl^ acetone (M^−1^)^[a]^	1012	>10^5^	18543	18	440	66	–^[j]^
2	*K*_1_^Cl^ acetone+2.5 % D_2_O (M^−1^)^{a]^	–^[h]^	6073	156	–^[j]^	–^[j]^	–^[j]^	–^[j]^
3	*c*log*P*^[b]^	4.7	5.2	4.9	5.1	5.4	5.1	–^[j]^
4	EC_50_ (μM)^[c]^	0.048±0.003	0.003±0.001	0.032±0.003	2.7±0.1	0.9±0.01	1.2±0.09	–^[j]^
5	EC_50_ (mol %)^[c]^	0.15	0.01	0.1	8.6	2.9	3.8	–^[j]^
6	n^[d]^	1.9±0.2	0.7±0.1	1.1±0.1	3.4±0.6	3.9±0.8	2.8±0.5	–^[j]^
7	EC_50_^NMDG^ (μM)^[e]^	0.28±0.02	0.048±0.01	0.8±0.2	–^[j]^	5.4±0.3	6.5±0.1	0.016±0.002
8	n^NMDG [d,e]^	1.7±0.2	1.0±0.1	0.5±0.1	–^[j]^	2.4±0.3	2.3±0.1	1.2±0.2
9	EC_50_^Gramicidin^ (μM)^[e,f]^	0.178±0.004	0.009±0.002	0.012±0.002	–^[j]^	2.6±0.1	4.1±0.5	0.016±0.003
10	n^Gramicidin [d,f]^	2.4±0.1	1.2±0.3	1.0±0.1	–^[j]^	2.9±0.2	1.7±0.3	1.6±0.6
11	Selectivity (*S*)^[g]^	1.6±0.01	5.3±1.1	67±20	–^[j]^	2.1±0.1	1.6±0.2	1.0±0.02

[a] 1 : 1 association constant for chloride binding in acetone‐d_6_ or acetone‐d_6_+2.5 % D_2_O. Chloride added as the TBA salt. Errors less than <7 % [b] Calculated log*P*. [c] Effective concentration to reach 50 % of maximal activity in the HPTS assay, reported as both an absolute concentration and mol % relative to lipid. LUVs of POPC (mean diameter 200 nm, lipid concentration 31 μM) loaded with 1 mM HPTS, NaCl (100 mM) and buffered at pH 7.0 with 10 mM HEPES. 5 mM NaOH added to generate the transmembrane pH gradient. Errors at the 95 % confidence limit. [d] Hill coefficient. [e] As with [c], except that lipid concentration=100 μM, NMDG−Cl (100 mM) used in place of NaCl, and 5 mM NMDG solution added to generate pH gradient. [f] In the presence of 0.1 mol% gramicidin D. [g] Defined as EC_50_^NMDG^/EC_50_^Gramicidin^. An *S* value of >1 indicates transport of Cl^−^ is faster than H^+^/OH^−^ transport. [h] Anion induced chemical shift perturbations too small to determine association constant. [i] Compound previously reported, data from assay reproduces literature values.^[32]^ [j] Not determined.

**Figure 3:**
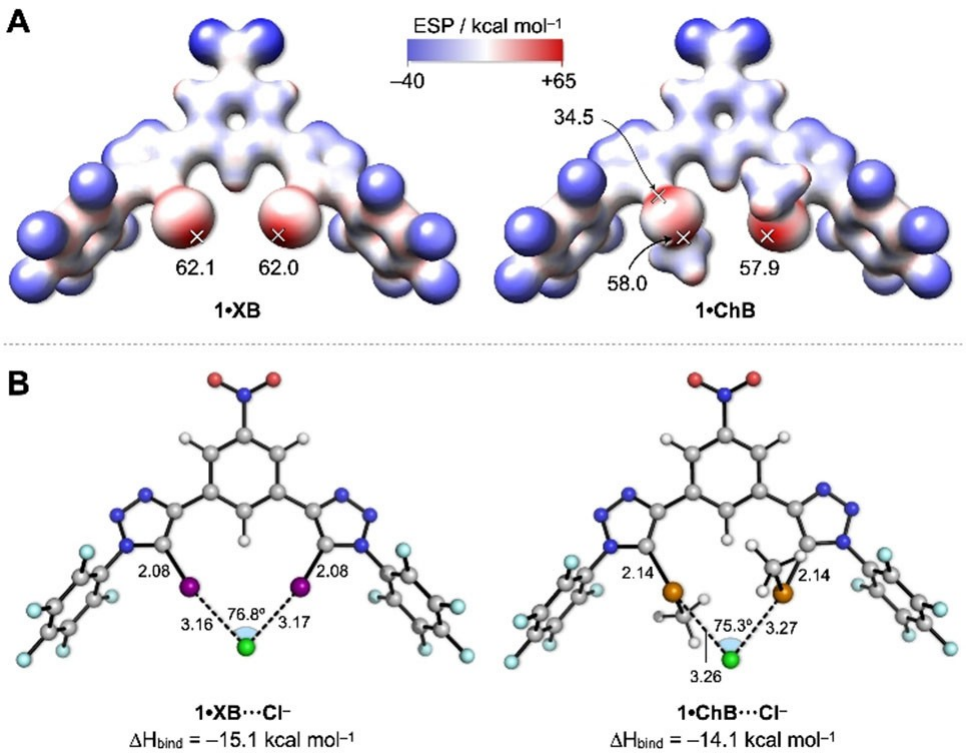
(A) Calculated electrostatic potentials (ESPs, kcal mol^−1^) on the van der Waal's surface of **1 ⋅ XB** and **1 ⋅ ChB**, with maximum values for sigma‐holes marked with crosses. (B) Calculated structures of the chloride complexes of **1 ⋅ XB** and **1 ⋅ ChB**, showing key distances (Å) and angles (°).

Calculation of the electrostatic potential (ESP) surface for **1 ⋅ XB** reveals one iodine‐centred sigma‐hole per atom (V_max_=62 kcal mol^−1^, Figure 3A).[^12^, ^44^] Similarly, the ESP of **1 ⋅ ChB** reveals the two tellurium‐centred sigma‐holes per Te atom, with the sigma‐hole opposite to the triazole C−Te bond being largest in size and magnitude (58.0 vs. 34.5 kcal mol^−1^). Computational studies were also used to probe the structures of the 1 : 1 anionophore−chloride complexes in chloroform implicit solvent as a membrane‐mimetic environment (Figure 3B). These revealed bidentate coordination of the chloride anion by **1 ⋅ XB** and **1 ⋅ ChB** in a 1 : 1 stoichiometric complex, in agreement with results from anion binding titration experiments. Calculated binding enthalpies suggest a stronger C−I⋯Cl^−^ interaction than C−Te⋯Cl^−^ (ΔΔH_bind_=−1.0 kcal mol^−1^, Table S4), in agreement with the trend from the NMR titrations and calculated ESP values. Compared with the calculated structures of **1 ⋅ XB** and **1 ⋅ ChB**, the Cl^−^‐complexed structures have slightly increased C−I/C−Te bond lengths (by 0.02 and 0.03 Å, respectively).

### Transmembrane anion transport activity

The anion transport capabilities of anionophores **1** and **2** were determined using 1‐palmitoyl‐2‐oleoyl‐sn‐glycero‐3‐phosphocholine large unilamellar vesicles (POPC LUVs), loaded with 8‐hydroxypyrene‐1,3,6‐trisulfonate (HPTS) and buffered to pH 7.0 in NaCl aqueous solution. A pH gradient was applied across the membrane by addition of sodium hydroxide, followed by addition of the carrier as a DMSO solution (<0.5 % v/v). The ability of the anionophore to dissipate the pH gradient by transmembrane OH^−^/Cl^−^ exchange was determined by recording the change in the HPTS emission, *I*_rel_ (λ_em_=510 nm), with time following excitation at λ_ex_=405/465 nm. At the end of each experiment, excess detergent was added to lyse the vesicles for calibration.

**Figure 4:**
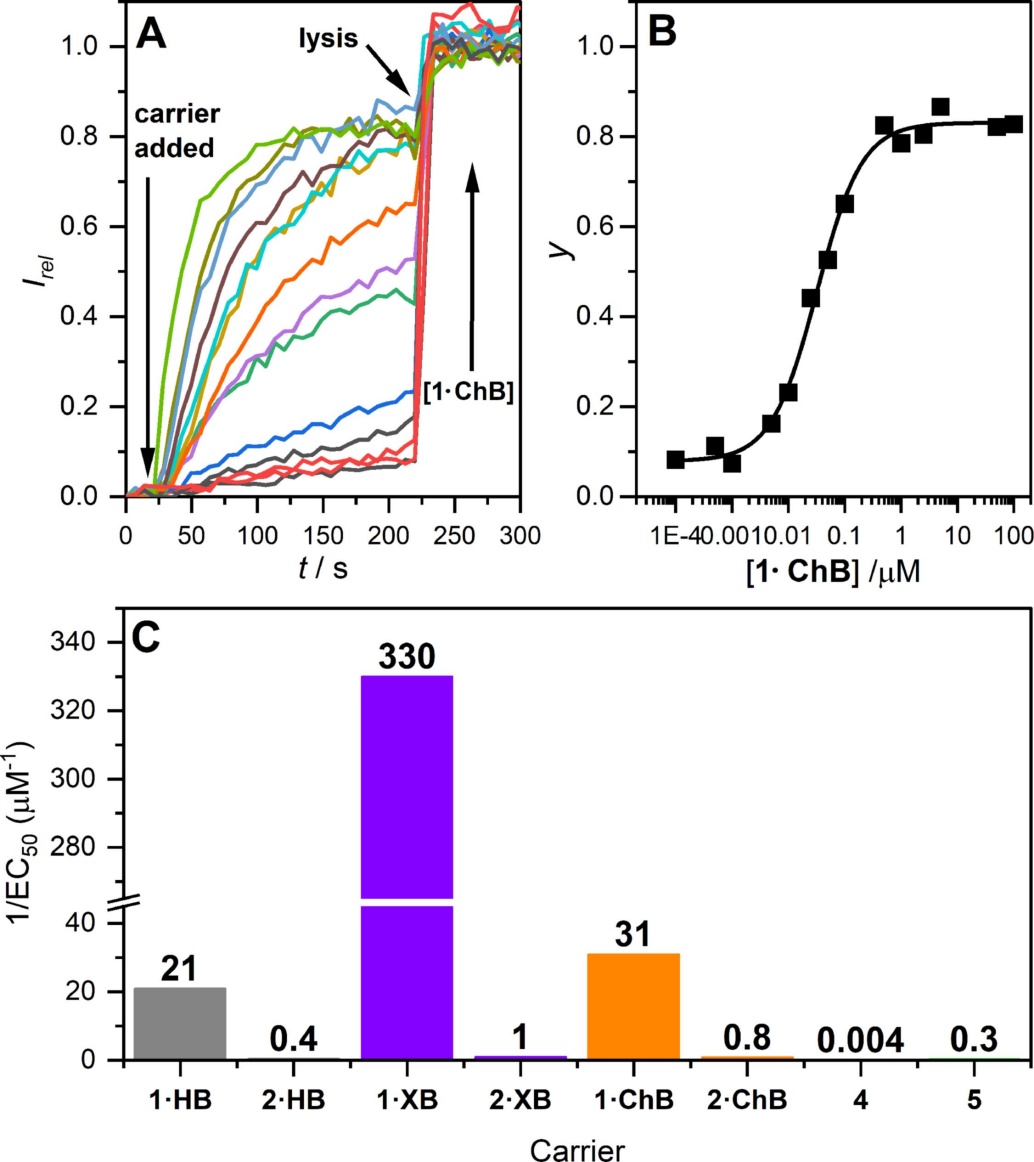
Anion transport in the HPTS assay. Data for **1 ⋅ ChB** is shown in (A) and (B). (A) Change in ratiometric emission (λ_em_=510 nm; λ_ex1_=405 nm, λ_ex2_=460 nm) upon addition of **1 ⋅ ChB** in DMSO (10^−4^–10^2^ μM) to POPC vesicles containing 1 mM HPTS, 100 mM internal and external NaCl, buffered with 10 mM HEPES at pH 7.0. A pH gradient is generated by addition of a NaOH base pulse (0.5 M). Addition of **1 ⋅ ChB** in DMSO initiates the experiment, and vesicle lysis by Triton X‐100 calibrates the assay. (B) Dependence of fractional activities (*y*, the relative intensity at *t*=288 s just prior to lysis) on concentration of **1 ⋅ ChB** (black squares), and fit to the Hill equation (black line). (C) Summary of transport activity data (reported as 1/EC_50_). Values for control XB anionophores **4** and **5** taken from ref. [15].

This assay was used to determine the concentration dependence of the activity of each anion carrier (Table 1, entry 4 and 5, with the corresponding calculated log*P* values^[45]^ in entry 3). Transport data for **1 ⋅ ChB** is shown in Figures 4A and B (see the Supporting Information for data for all other compounds). The activity of all three bidentate carriers **1** was high (nM activity, EC_50_<0.05 mol% with respect to lipid) and followed the chloride binding affinity trend of **1 ⋅ XB**>**1 ⋅ ChB**>**1 ⋅ HB** (Figure 4C) The activity of XB donor **1 ⋅ XB** was remarkably high (3 nM; 0.01 mol%), and to the best of our knowledge, is the most active XB anionophore reported to date. The activity exceeds that of the archetypal XB donor iodoperfluorobenzene **4** (Figure 1C) by five orders of magnitude (EC_50_=260 μM),^[15]^ and exceeds iodoperfluorohexane **5** (EC_50_=3 μM)^[15]^ as well as our previously reported iodotriazole XB anionophores^[18]^ by a factor of 10^3^ under identical assay conditions.

The activity of chalcogen bonding anion carrier **1 ⋅ ChB** was also excellent (EC_50_=32 nM), favourably comparing to the previously reported bis(pentafluorophenyl)tellurium anionophore (EC_50_=200 nM).^[21]^ The monodentate control compounds **2** were less active, consistent with reduced chloride binding affinity, but maintained the transport activity trend of XB>ChB>HB.

Hill coefficients *n*>1 reveals endergonic self‐assembly (un‐stable supramolecules active in the transport process) and provides information on the stoichiometry; *n*=1 or *n*<1 reveals exergonic self‐assembly (stable supramolecules).^[46]^ Hill analysis of the dose response curves for the monodentate anionophores **2** reveals *n*∼3 (Table 1, entry 6), implying that such trimers represent a minority population in the membrane, with the majority coexisting as inactive monomers. Hill coefficients of ∼1 suggest active monomeric complexes for bidentate **1 ⋅ XB** and **1 ⋅ ChB**, in agreement with results from titrations and calculation, and validating our approach of incorporating multiple XB/ChB donors within a single anionophore to maximise transport efficiency. Hydrogen bonding anionophore **1 ⋅ HB**, with n∼2, suggests dimeric transport species at work.

**Figure 5:**
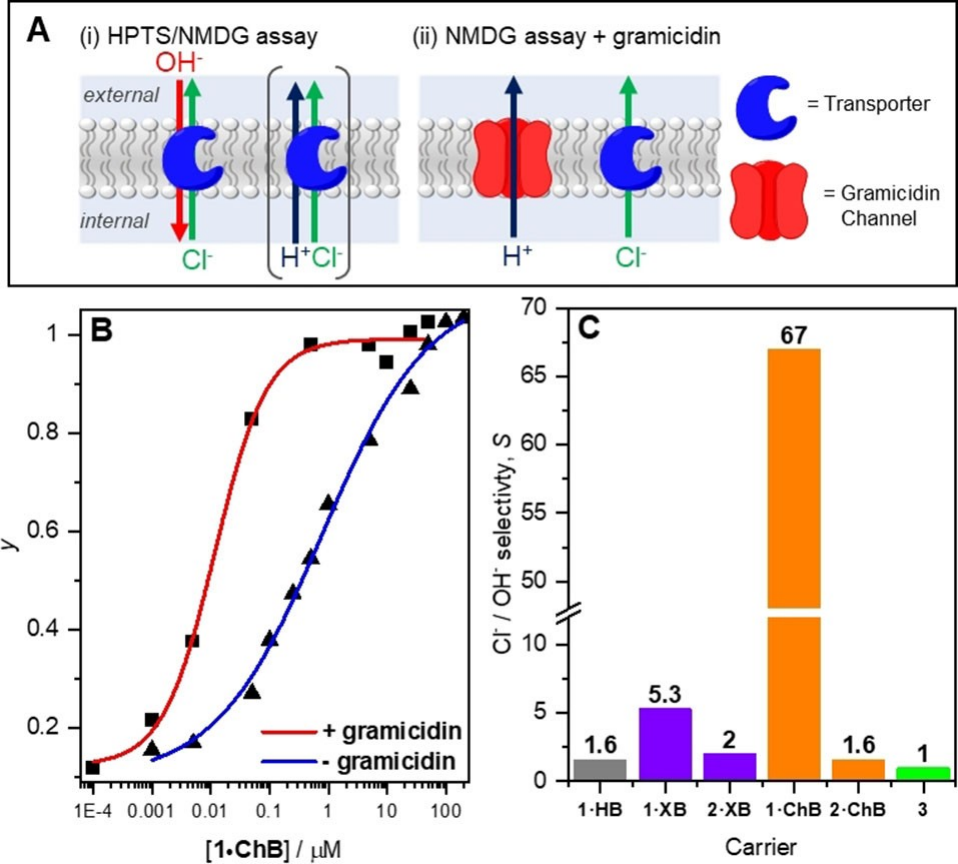
(A) LUV‐based transport assays. (i) Transport mechanisms occurring in the HPTS and NMDG assays in the absence of gramicidin: Cl^−^/OH^−^ exchange (antiport), or H^+^/Cl^−^ co‐transport (symport). The antiport mechanism dominates the transport in **1 ⋅ XB**, **1 ⋅ ChB** and **1 ⋅ HB** due to a lack of basic atoms for protonation. (ii) Coupled electrogenic carrier‐mediated chloride transport and electrogenic channel‐mediated proton transport mechanism occurring in the NMDG assay in the presence of gramicidin. (B) Dependence of fractional activities (*y*, the relative intensity at *t*=288 s just prior to lysis) on concentration of **1 ⋅ ChB** in the presence (▪) and absence (▴) of gramicidin, and fit to the Hill equation (red and blue solid lines, respectively). (C) Summary of Cl^−^>OH^−^ transport selectivity data, where *S*=EC_50_^NMDG^/EC_50_^Gramicidin^.

The transport activities of all anionophores were independent of iso‐osmolar replacement of the external Na^+^ cation with Li^+^, K^+^ or Rb^+^ (see the Supporting Information), indicative of selective anion transport (OH^−^/A^−^ antiport or H^+^/A^−^ symport, Figure 5A.i). Replacement of the zwitterionic POPC lipids in the HPTS assay with anionic egg yolk phosphatidylglycerol (EYPG) lipids led to a significant decrease in observed activity, consistent with the requirement for formation of an anionic complex in the rate limiting transport process (Figure S45). Negligible transport upon replacement of chloride with the hydrophilic gluconate anion provided further evidence into the anion transport mechanism, by demonstrating that neither OH^−^/gluconate antiport or H^+^/gluconate symport mechanisms are active, and that the anion‐independent H^+^/Na^+^ cation antiport process is negligible (Figure S46). Given the low basicity of the triazole anionophores (p*K*_aH_∼0–1),^[47]^ H^+^/Cl^−^ symport is improbable and the transport is likely dominated by a Cl^−^/OH^−^ antiport mechanism, in agreement with observations from previous studies on XB‐mediated anion transport.[^15^, ^17^] Inactivity in the carboxyfluorescein (CF) dye leakage assay ruled out non‐specific leakage and membrane damage by the anion carriers (Figure S47). Suppression of carrier activity in gel phase dipalmitoylphosphatidylcholine (DPPC) LUVs at 25 °C with transporters **1** and **2**, and restoration of activity in the fluid lipid phase at 45 °C, provided evidence for a mobile carrier mechanism, ruling out formation of a membrane‐spanning channel structure whose activity would be independent of the lipid phase (Figure S48).

### Chloride over hydroxide transport selectivity

To investigate Cl^−^>OH^−^ selectivity of the anionophores, we adapted the pH‐gradient dissipation assay by replacing NaCl with *N*‐methyl‐d‐glucamine chloride (NMDG−Cl) in the internal and external buffer, and the sodium hydroxide base pulse with NMDG (5 mM). This “NMDG assay” has been recently reported by Gale, Davis and co‐workers,[^32^, ^48^] and is used here to facilitate direct comparison with their extensive analysis of Cl^−^>H^+^/OH^−^ selectivity of a range of anionophores.^[32]^ The assay employs the proton channel gramicidin D to investigate the relative rates of Cl^−^ and H^+^/OH^−^ transport by facilitating fast electrogenic H^+^ transport, and therefore an overall coupled H^+^/Cl^−^ symport process (Figure 5A.ii). In the case where H^+^/OH^−^ transport is rate limiting, addition of gramicidin leads to acceleration of the pH gradient dissipation; where Cl^−^ is rate limiting, the rate of pH gradient dissipation is unaffected by gramicidin. The ratio of EC_50_ values in the absence and presence of gramicidin provides a measure of the Cl^−^>H^+^/OH^−^ selectivity, *S*, (where *S*=EC_50_^NMDG^/EC_50_^gramicidin^) under the assay conditions. For triazole derivatives **1** and **2**, given the probable Cl^−^/OH^−^ antiport mechanism (as opposed to H^+^/Cl^−^ symport), the assay will report on Cl^−^>OH^−^ selectivity.

Representative dose‐response curves for **1 ⋅ ChB** are shown in Figure 5B, and selectivity data for the bidentate anionophores are shown in Table 1, entry 11, and Figure 5C. In each case, the transport activity is enhanced upon addition of 0.1 mol% gramicidin, revealing selective Cl^−^ transport. Excellent Cl^−^>OH^−^ selectivity was determined for XB‐donor **1 ⋅ XB** (*S*=5.3) in comparison to the analogous hydrogen bonding **1 ⋅ HB** which exhibited little selectivity (*S*=1.6). Incorporation of chalcogen bonding telluromethyl groups in **1 ⋅ ChB** led to exceptional selectivity (*S*=67), despite the simple, planar bidentate structure of the anionophore. For comparison, hydrogen bonding thiourea **3** (Figure 1C) is not chloride selective (*S*=1), and to date only a handful of compounds have been reported with *S*>10, and in one case up to 100, which are based around complex hydrogen bonding tripodal or preorganised macrocyclic cholaphane scaffolds.^[32]^ In HB anionophores enhancing anion transport by increasing receptor acidity (such as by adding electron withdrawing groups) is severely detrimental to the selectivity. Importantly, however, high activity *and* Cl^−^>OH^−^ selectivity is achieved in XB and ChB anionophores **1 ⋅ XB** and **1 ⋅ ChB**, despite the significant electron withdrawing nature of the substituents.

### Computational studies

**Figure 6:**
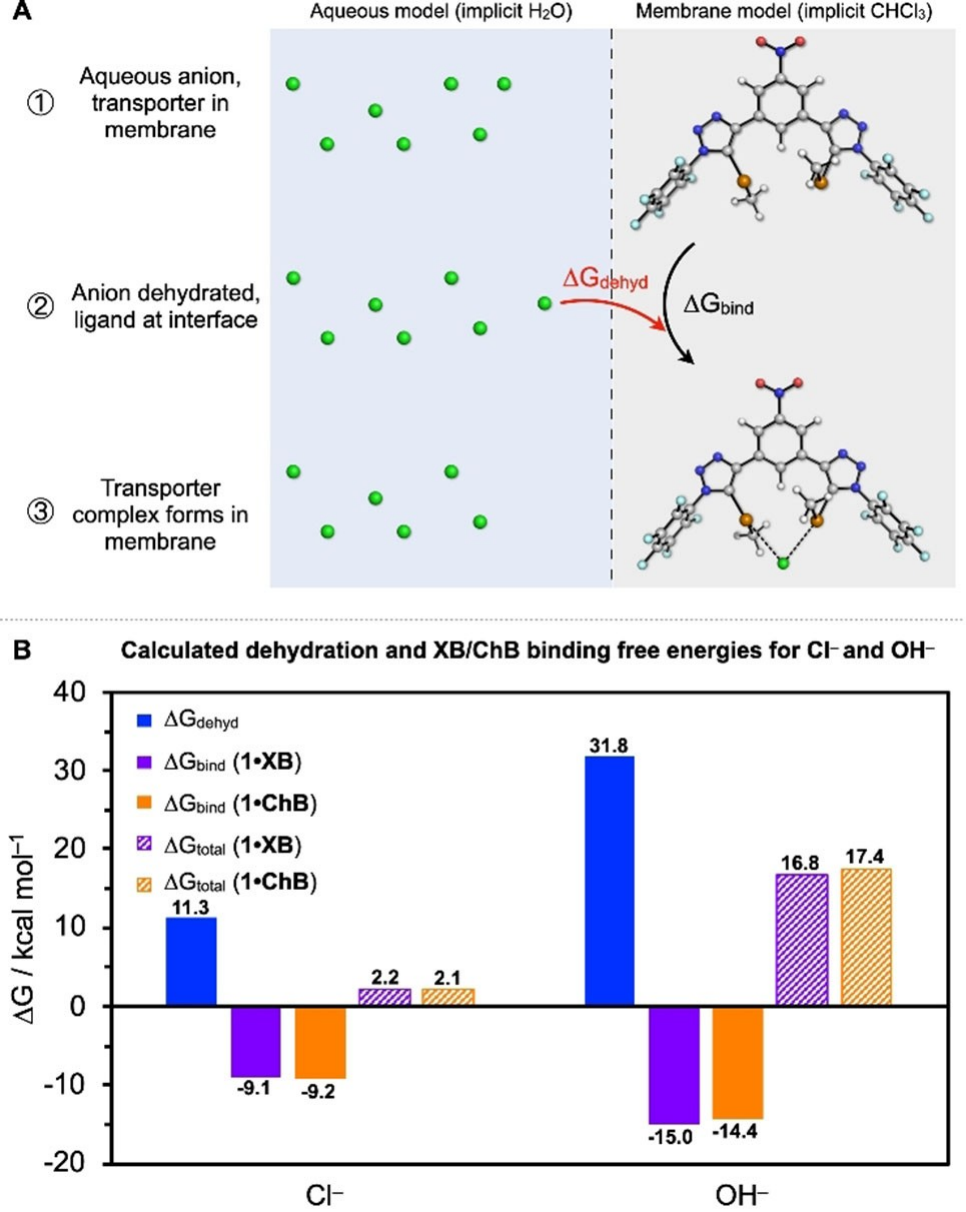
(A) Schematic representation of the computational model for anion binding at a membrane/water interface (B) Calculated dehydration and binding free energies (298 K) for **1 ⋅ XB** and **1 ⋅ ChB** with Cl^−^ and OH^−^. Computational protocols to calculate ΔG_dehyd_ and ΔG_bind_ are described in the Supporting Information.

To further explore the role of sigma‐hole mediated anion recognition within the transmembrane anion transport process, we developed a computational model that accounts for both anion binding affinity to the XB and ChB anionophores, and chloride and hydroxide anion dehydration at the aqueous‐membrane interface (Figure 6A). This model assumes that the hydrophobic transporter is confined within the membrane bilayer, and binds the anion at the water/membrane interface. This binding event incurs an anion dehydration penalty, where the equilibrium position depends on the balance of anion dehydration with the strength of binding to the transporter. To describe this process, we computed the dehydration free energies (ΔG_dehyd_) for Cl^−^ and OH^−^ transfer from implicit water to chloroform (as a membrane‐mimetic environment), and the respective binding free energies (ΔG_bind_) of these anions to **1 ⋅ XB** and **1 ⋅ ChB** in implicit chloroform (Figure 6B).^[49]^ The sum of these terms describes the anion transport process: ΔG_total_=ΔG_dehyd_+ΔG_bind_. Dehydration is strongly disfavoured for OH^−^ (ΔG_dehyd_=31.8 kcal mol^−1^), a value which may also be underestimated by up to 10 kcal mol^−1^ due to the use of an implicit solvent model (see the Supporting Information for further details).^[50]^

In comparison, ΔG_dehyd_ is only 11.3 kcal mol^−1^ for Cl^−^. The trends are reversed for the binding free energies in chloroform (ΔG_bind_), where binding of OH^−^ to **1 ⋅ XB** is preferred over Cl^−^ (−15.0 vs. −9.1 kcal mol^−1^, respectively). This prediction is opposite to the Cl^−^>OH^−^ transport selectivity observed experimentally, likely due to a combination of stronger binding and underestimation of short‐range solvent‐OH^−^ interactions with implicit solvent models. However, when considering the anion transport process in full, the stronger binding of OH^−^ to **1 ⋅ XB**/**1 ⋅ ChB** compared with Cl^−^ is insufficient to overcome the greater penalty of dehydration associated with this anion.

Due to the strongly disfavoured OH^−^ dehydration process, we also considered the possibility that a water molecule could accompany the OH^−^ anion into the membrane to stabilise the naked anion. In this case we also found the same strong preference for Cl^−^ transport over OH^−^, and a slight increase in anion selectivity for **1 ⋅ ChB** vs. **1 ⋅ XB** (see the Supporting Information). From these computational models, it is clear that the role of anion dehydration is vital to explain the observed anion selectivity, where our model suggests a bias for Cl^−^ over OH^−^ transport of ∼15 kcal mol^−1^. We also observed slightly stronger anion binding with **1 ⋅ XB** than **1 ⋅ ChB**, in qualitative agreement with the experimental activity.

Overall, these results demonstrate the critical role of anion dehydration in the transport process. Furthermore, XB and ChB mediated anion binding interactions are known to be more hydrophobic than acidic hydrogen bond donors, exhibiting a significant covalent contribution to the bonding,[^9^, ^34^, ^51^, ^52^] and anecdotally possess halide>oxoanion binding selectivity.[^12^, ^37^, ^53^, ^54^, ^55^, ^56^] We suggest that a combination of these effects contribute to the observed Cl^−^>OH^−^ selectivity concomitant with high activity, whereby binding and transport of the less hydrated and less basic chloride anion is favoured.

## Conclusion

We report novel bidentate halogen bonding and chalcogen bonding iodo‐ and telluromethyl‐triazole derivatives that act as highly active anionophores. Importantly, anion transport experiments reveal both record nanomolar activity for the bidentate halogen bonding carrier and outstanding Cl^−^>OH^−^ transport selectivity for the chalcogen bonding anionophore. The unique nature of such sigma‐hole interactions enables both high activity and high selectivity to be achieved in a relatively simple molecular design. This is in contrast to hydrogen bonding anion carriers where more acidic hydrogen bond donors lead to decreased selectivity despite enhancing activity. Using a computational model that accounts for both anionophore−anion binding in a membrane‐mimetic environment and anion solvation, we reveal the role of sigma‐hole mediated anion binding and dehydration in achieving efficient and selective anion transport. These results demonstrate how the judicious selection of sigma‐hole non‐covalent interactions within abiotic anionophores is a powerful approach to engineering selectivity and activity, and also highlight the significant potential for exploiting halogen and chalcogen bonding interactions in biological contexts. Future applications in channelopathy therapeutics and tools for physiological research where both high selectivity and activity is required may be envisaged.

## Experimental Section

**Crystallographic data**: Deposition numbers 2075766 (for 1⋅XB) and 2075767 (for 1⋅ChB) contain the supplementary crystallographic data for this paper. These data are provided free of charge by the joint Cambridge Crystallographic Data Centre and Fachinformationszentrum Karlsruhe Access Structures service.

## Conflict of interest

The authors declare no conflict of interest.

## Supporting information

As a service to our authors and readers, this journal provides supporting information supplied by the authors. Such materials are peer reviewed and may be re‐organized for online delivery, but are not copy‐edited or typeset. Technical support issues arising from supporting information (other than missing files) should be addressed to the authors.

Supporting Information
