# Supplementary material for: Highly Active Halogen Bonding and Chalcogen Bonding Chloride Transporters with Non‐Protonophoric Activity
Source: Chemistry. 2021 Jun 14;27(45):11738–45. doi: 10.1002/chem.202101681 (PMC8453555; doi:10.1002/chem.202101681)
Supplement: Supplementary file 1 — Supporting Information [file CHEM-27-11738-s001.pdf]

Chemistry–A European Journal

Supporting Information

Highly Active Halogen Bonding and Chalcogen Bonding Chloride Transporters with Non-Protonophoric Activity

Laura E. Bickerton[+], Andrew Docker[+], Alistair J. Sterling, Heike Kuhn, Fernanda Duarte,* Paul D. Beer,* and Matthew J. Langton*

Contents

Contents 1

1. Materials and methods 2
2. Synthesis and characterization 3
3. Single crystal X-ray diffraction experiments 20
4. Anion transport experiments 22
5. NMR titration experiments 34
6. Computational 40
7. References 47

1. Materials and methods

All reagents and solvents were purchased from commercial sources and used without further purification. Lipids were purchased from Avanti polar lipids and used without further purification. Where necessary, solvents were dried by passing through an MBraun MPSP-800 column and degassed with nitrogen. Column chromatography was carried out on Merck® silica gel 60 under a positive pressure of nitrogen. Where mixtures of solvents were used, ratios are reported by volume. NMR spectra were recorded on a Bruker AVIII 400, Bruker AVII 500 (with He cryoprobe) and Bruker AVIIIHD 500 spectrometers. Chemical shifts are reported as δ values in ppm. Mass spectra were carried out on an Agilent 6120 bench-top single quadrupole, a Waters LCT Premier XE benchtop (oa-TOF) and a Thermo Exactive High-Resolution Orbitrap FTMS spectrometer. Fluorescence spectroscopic data were recorded using a Horiba Duetta fluorescence spectrophotometer, equipped with Peltier temperature controller and stirrer. Experiments were conducted at 25°C unless otherwise stated. Vesicles were prepared as described below using Avestin "LiposoFast" extruder apparatus, equipped with polycarbonate membranes with 200 nm pores. GPC purification of vesicles was carried out using GE Healthcare PD-10 desalting columns prepacked with Sephadex G-25 medium.

Abbreviations

$CDCl_3$: deuterated chloroform; CF: 5(6)-Carboxyfluorescein; ChB: Chalcogen Bonding; CuAAC: Copper(I)-catalyzed azide-alkyne cycloaddition; DCM: Dichloromethane; DMF: *N*,*N*-Dimethylformamide; DMSO: Dimethylsulfoxide; DPPC: 1,2-dipalmitoyl-sn-glycero-3-phosphocholine; EC_{50}: Effective concentration; EDTA: Ethylenediaminetetraacetic acid; EtOAc: Ethyl acetate; EYPG: egg-yolk phosphatidylglycerol; FCCP: Carbonyl cyanide-p-trifluoromethoxyphenylhydrazone; HB: Hydrogen Bonding; HEPES: *N*-(2-hydroxyethyl)piperazine-*N*'-(2-ethanesulfonic acid); HPTS: 8-hydroxy-1,3,6-pyrenetrisulfonate; HRESI MS: High resolution electrospray ionisation mass spectrometry; KF: Potassium Fluoride; KOH: Potassium hydroxide; LUVs: large unilamellar vesicles; MeCN: Acetonitrile; MeOH: Methanol; NaCl: Sodium chloride; NMDG-Cl: N-methyl-D-glucamine chloride; POPC: 1-palmitoyl-2-oleoyl-sn-glycero-3-phosphocholine; rt: Room temperature; TBA salt: Tetrabutylammonium salt; TBTA: Tris((1-benzyl-4-triazolyl)methyl)amine; THF: Tetrahydrofuran; VdW: Van der Waals; XB: Halogen Bonding.

2. Synthesis and characterization

Warning! Low molecular weight organic azides used in this study are potentially explosive and should be used on a small scale. Appropriate protective measures should always be taken when handling these compounds.

Tris[(1-benzyl-1H-1,2,3-triazol-4-yl)methyl]amine (TBTA)[1], azido-pentafluorobenzene[2] and 1,3-diethynylnitrobenzene[3] were prepared according to literature procedures. Preparation of silver acetylide intermediates (general procedure 2) were conducted via a reported procedure.[4]

General Procedure 1

$Cu(CH_3CN)_4PF_6$ (0.1 equivalents per alkyne) and TBTA (0.1 equivalents per alkyne) were stirred in anhydrous degassed DCM (ca. 5ml per 0.30 mmol of alkyne) for 10 minutes under a nitrogen atmosphere, followed by the addition of iodo/proto-alkyne and aryl azide (1.5 equivalents per alkyne) and left to stir overnight. The reaction mixture was diluted with DCM (ca. 50 mL) and the organic phase washed with 10% aqueous EDTA/NH_4OH solution (2 x 10 mL), followed by brine (20 mL) and subsequently dried over $MgSO_4$. The solvent was removed *in vacuo* to give the crude product. The residue was purified by silica gel flash chromatography using either DCM/MeOH or DCM/EtOAc mixtures as the eluent.

General Procedure 2

The appropriate alkyne (0.30 mmol) was dissolved in the minimum amount of MeOH (ca. 10 mL per 0.30 mmol of alkyne) to which was added $AgNO_3$ (1 equivalent per alkyne) followed by one drop of concentrated NH_4OH (0.1 mL) immediately inducing precipitation of the desired silver acetylide. The reaction mixture was left to stir for 30 minutes at room temperature, after which time the solvent was removed *in vacuo*. The silver acetylide was subsequently suspended in anhydrous THF (20 mL) under an atmosphere of nitrogen. In a separate flask Me_2Te_2 (0.50 equivalents per alkyne) was dissolved in THF (5 ml) and cooled to 0°C, to the solution of the ditelluride 1 M of Br_2 in CH_2Cl_2 (0.50 equivalents per alkyne) was added dropwise, after which time the ditelluride solution darkened indicating the formation of MeTeBr which was added immediately to the suspension of the silver acetylide dropwise. The reaction mixture was left to stir for 30 minutes at room temperature in the absence of light, after which time it was filtered through Celite and the reaction mixture was concentrated to dryness *in vacuo*. The crude telluromethyl alkyne was then used immediately for the following CuAAC reaction.

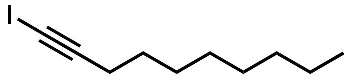

Iododecyne 6. Under N_2, decyne (3.623 mmol) and KOH (9.058 mmol) were stirred in MeOH for 10 mins, followed by the addition of I_2 (3.986 mmol). This was stirred at rt for 48 hours. The reaction was diluted with water and extracted with EtOAc (20 mL). The organic mixture was washed with saturated $Na_2S_2O_3$ solution (2 x 10 mL), followed by brine (20 mL) and then dried over $MgSO_4$. The solvent was removed *in vacuo* to give the pure product (quant.). Characterisation data aligns with the literature.[5] **^{1}H NMR** (400 MHz, $CDCl_3$) δ 2.35 (t, J = 7.1 Hz, 2H, CH), 1.51 (m, 2H, CH_2), 1.36-1.28 (m, 10H, 5 x CH_2), 0.88 (t, J = 6.9 Hz, 3H, CH_3).

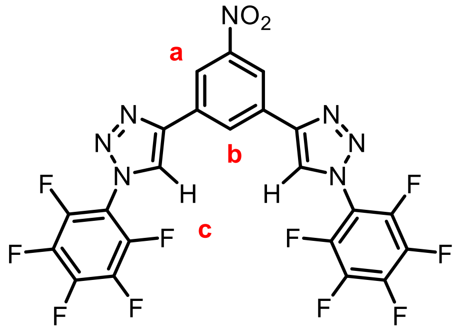

Carrier 1•HB. Synthesised according to General Procedure 1 from 1,3-diethynyl-5-nitrobenzene (71 mg, 0.411 mmol) to yield **1•HB** as a white solid (126 mg, 0.214 mmol, 52%). **^{1}H NMR** (500 MHz, DMSO) δ 9.51 (s, 2H$_c$), 8.99 (t, *J* = 1.5 Hz, 1H$_b$), 8.78 (d, *J* = 1.5 Hz, 2H$_a$). **^{19}F NMR** (470 MHz, DMSO) δ -146.74 – -147.52 (m), -150.65 – -151.27 (m), -160.45 – -161.21 (m). **^{13}C NMR** (126 MHz, DMSO) δ 149.19, 144.89, 141.95 (dm, *J* = 255 Hz), 137.74 (dm, *J* = 253 Hz), 131.93, 127.96, 126.07, 119.83, 112.36 (m). **HRMS** (ESI+ve) m/z: 590.0415 ([M+H]$^+$, $C_{22}H_6O_2N_7F_{10}$ requires 590.0418)

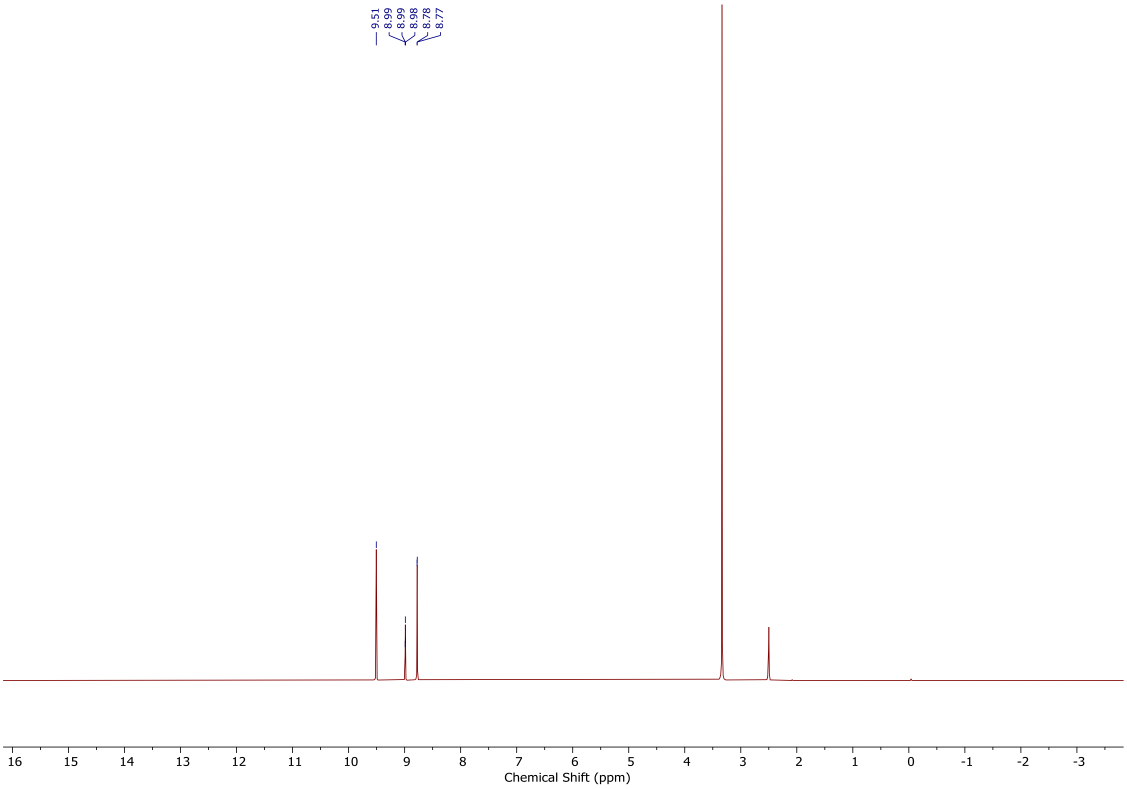

Figure S1. ^{1}H NMR Spectrum of **1•HB** (DMSO-d$_6$, 298 K, 500 MHz).

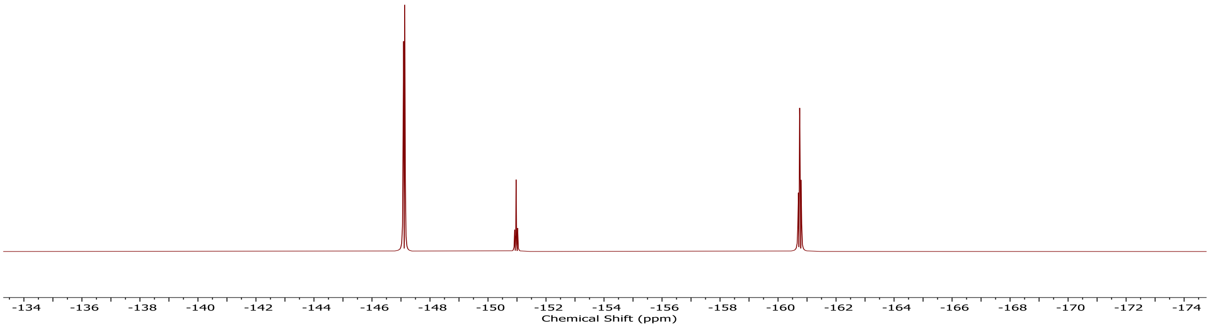

Figure S2. ^{19}F NMR Spectrum of **1•HB** (DMSO-d_6, 298 K, 470 MHz).

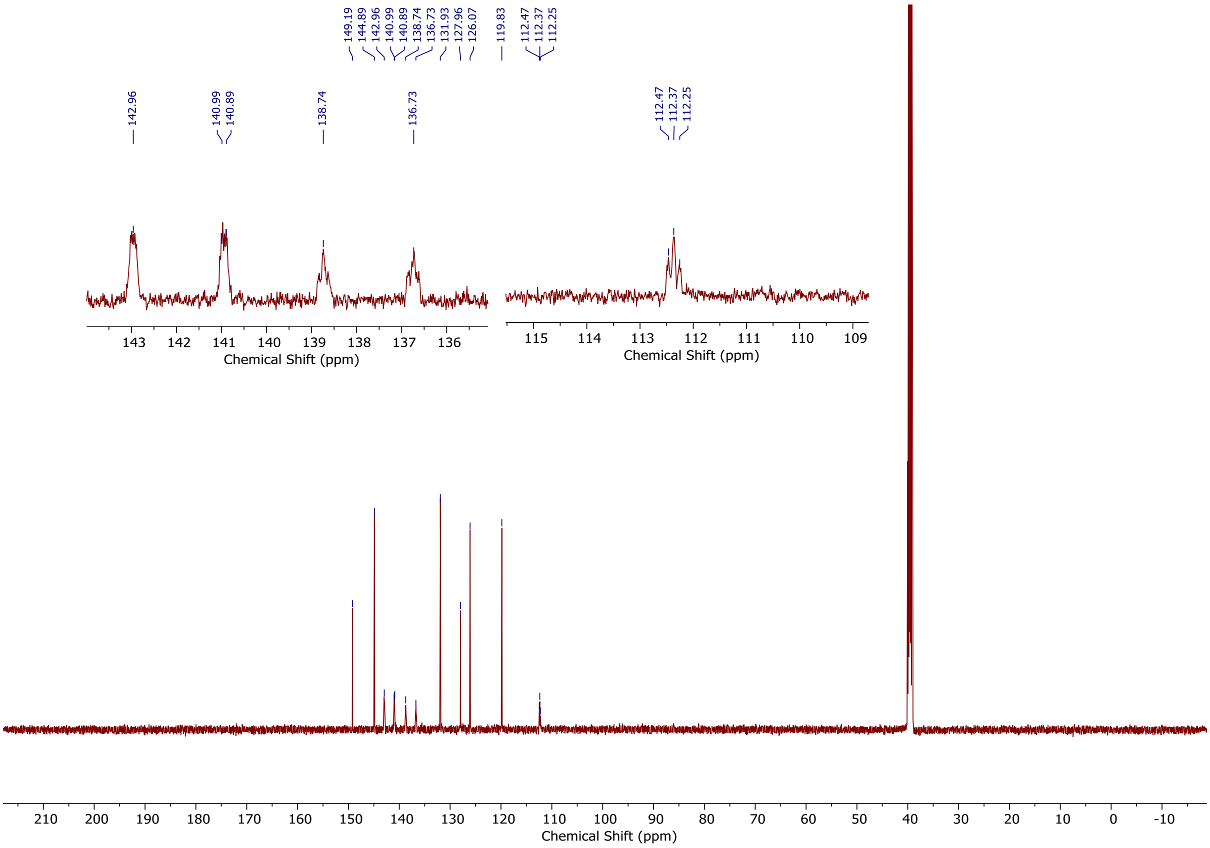

Figure S3. ^{13}C NMR Spectrum of **1•HB** (DMSO-d_6, 298 K, 126 MHz).

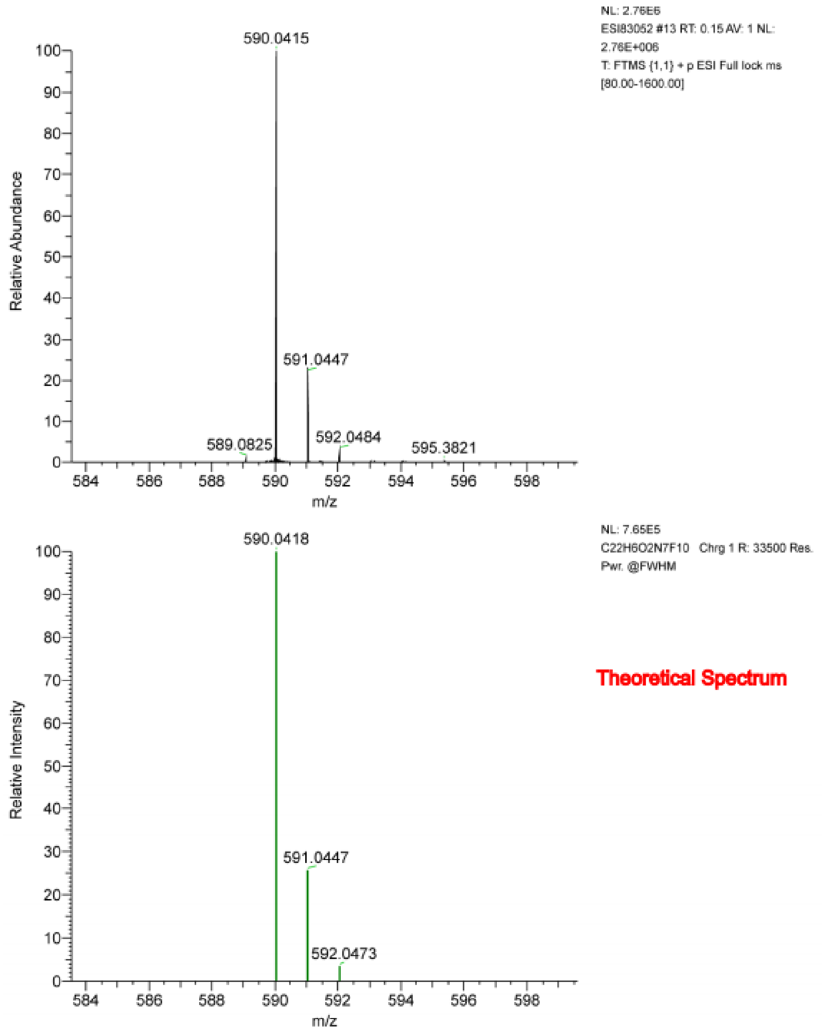

Figure S4. HRESI spectrum of **1•HB**.

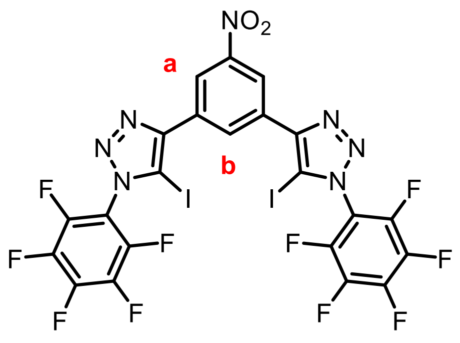

Carrier 1•XB. 1,3-diethynyl-5-nitrobenzene (100 mg, 0.584 mmol) was dissolved in anhydrous THF to which was added CuI (11 mg, 0.0583 mmol) and *N*-iodomorpholine hydroiodide (656 mg, 1.93 mmol) and left to stir for 4 hours in the absence of light. After which time the reaction mixture was diluted with DCM (300 mL) and filtered through a DCM saturated pad of alumina, the filtrate was concentrated *in vacuo* and adsorbed onto silica. The dry loaded compound was eluted with 10% EtOAC/hexane mixtures (500 mL) and the filtrate concentrated to dryness *in vacuo*. The iodoalkyne **9** was isolated as a yellow solid in 83% yield and then immediately used in a CuAAC reaction with perfluorbenzene azide according to general procedure 1, to yield **1•XB** (428 mg, 0.509 mmol, 87%) as an off white solid. **^{1}H NMR** (500 MHz, $CDCl_3$) δ 9.20 (t, J = 1.6 Hz, 1H_b), 9.07 (d, J = 1.6 Hz, 2H_a). **^{19}F NMR** (470 MHz, $CDCl_3$) δ -142.18 (m), -146.09 – -146.76 (m), -158.38 – -158.93 (m). **^{13}C NMR** (126 MHz, $CDCl_3$) δ 149.00, 148.15, 143.83 (dm, J = 262 Hz), 138.21 (dm, J = 263 Hz), 131.79, 131.30, 122.31, 112.74 – 111.51 (m), 81.64, 29.84. **HRMS** (ESI+ve) m/z: 841.8337 ($[M+H]^+$ $C_{22}H_4O_2N_7F_{10}I_2$ requires 841.8351).

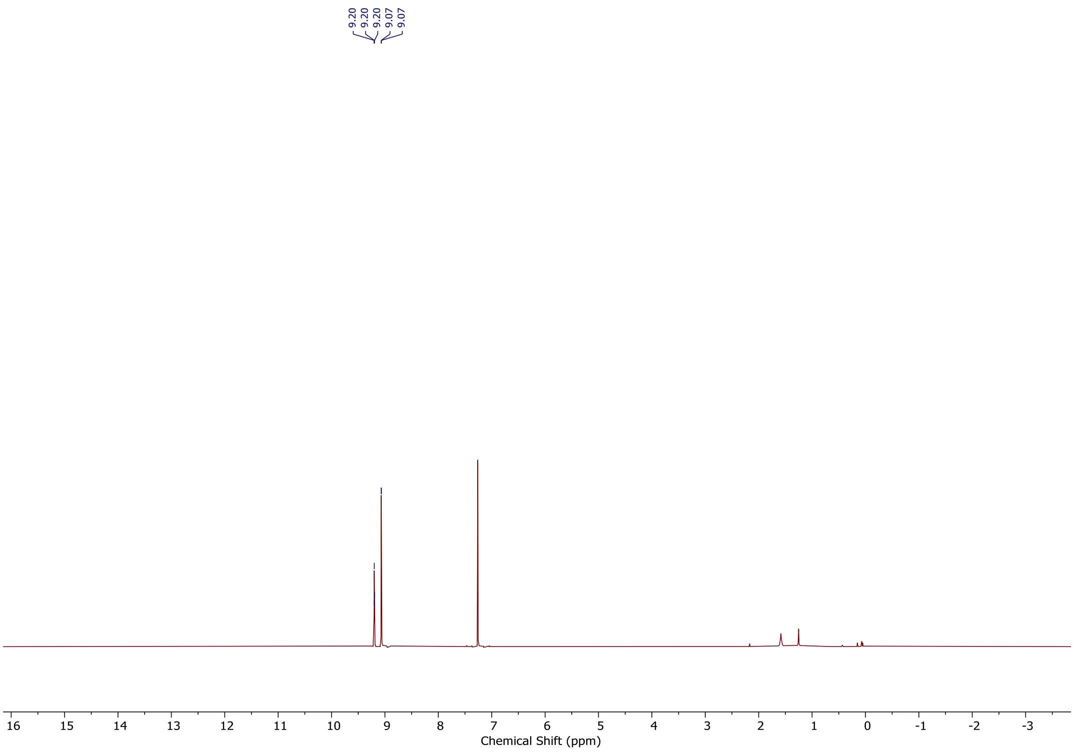

Figure S5. ^{1}H NMR Spectrum of **1•XB** ($CDCl_3$, 298 K, 500 MHz)**.**

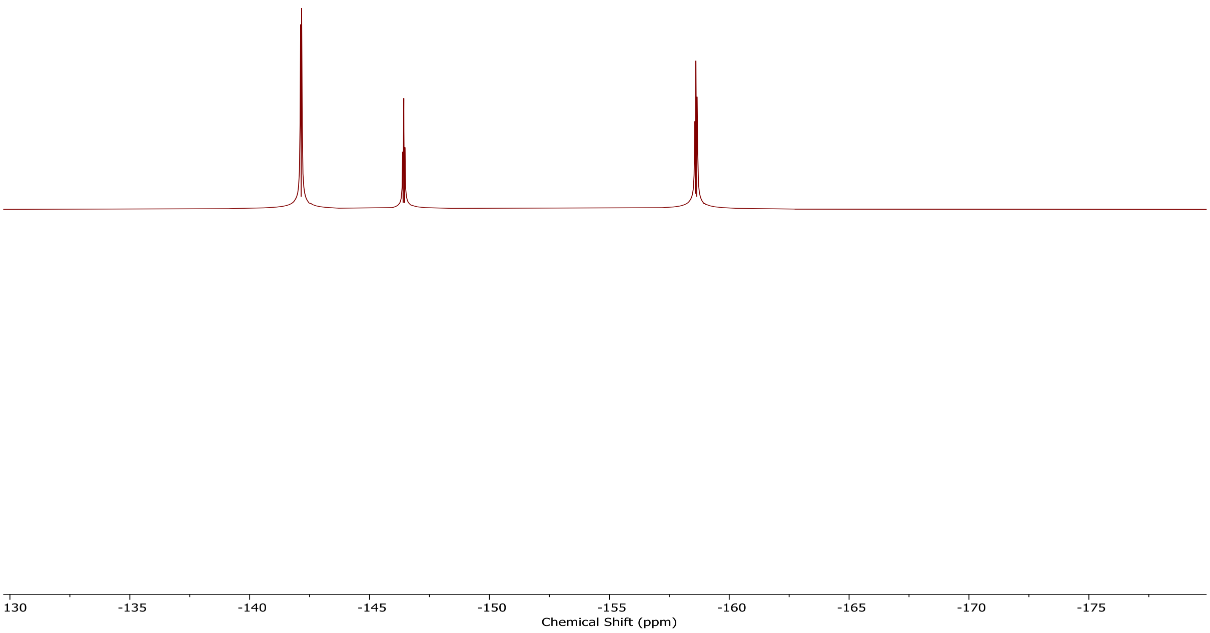

Figure S6. ^{19}F NMR Spectrum of **1•XB** ($CDCl_3$, 298 K, 470 MHz).

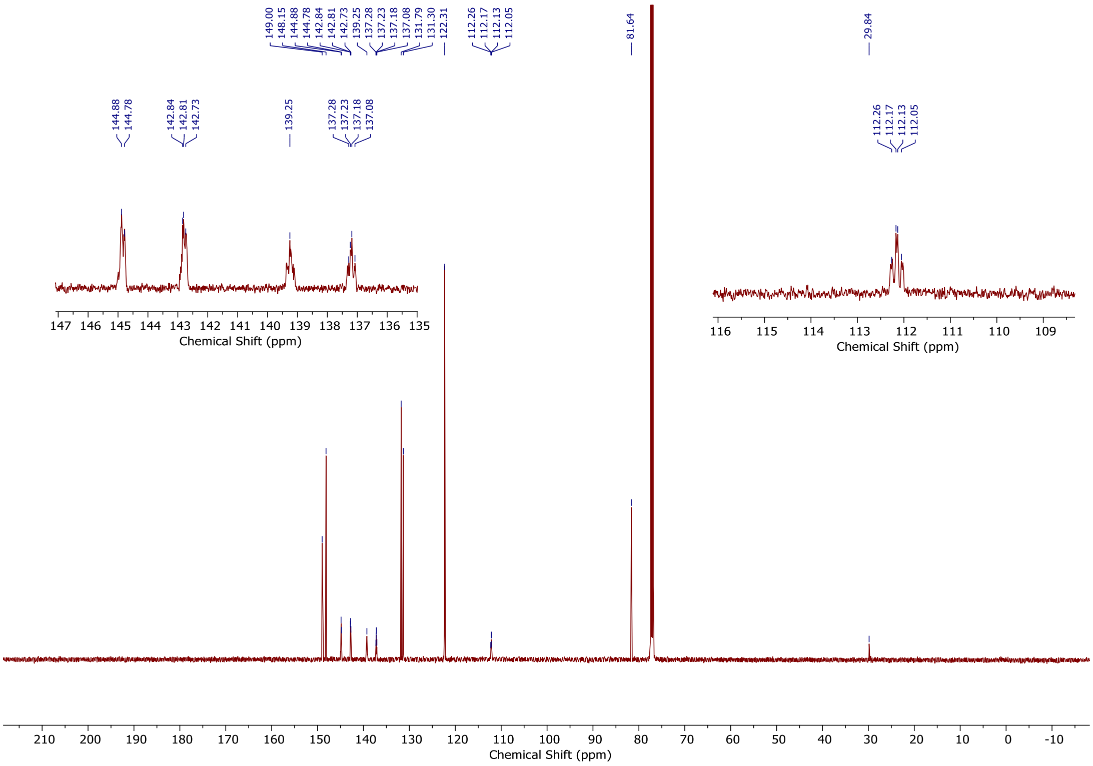

Figure S7. ^{13}C NMR Spectrum of **1•XB** ($CDCl_3$, 298 K, 126 MHz)**.**

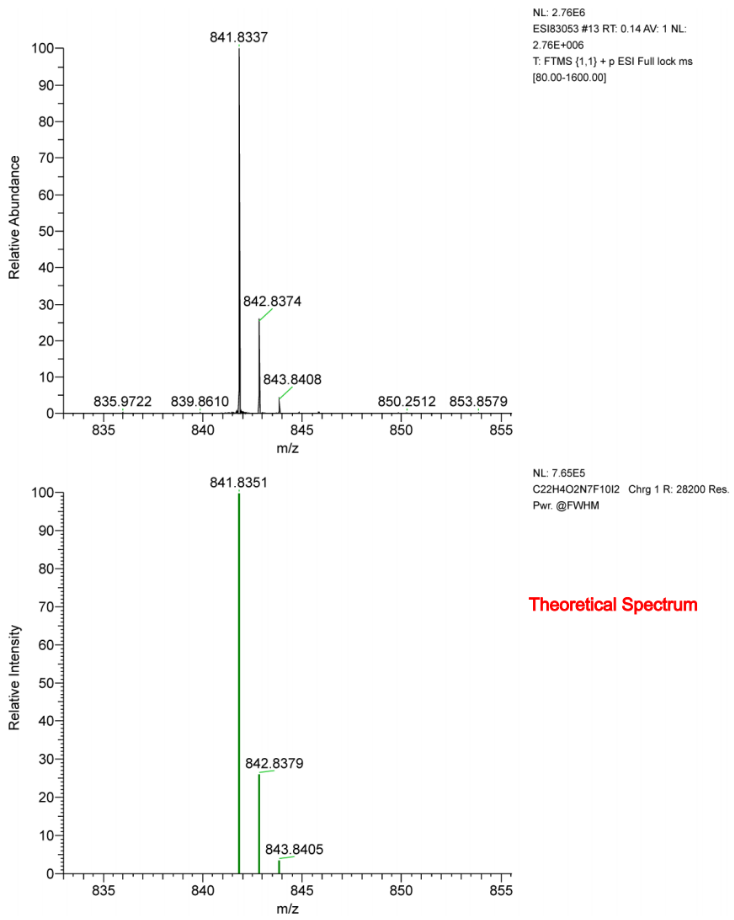

Figure S8. HRESI spectrum of **1•XB**.

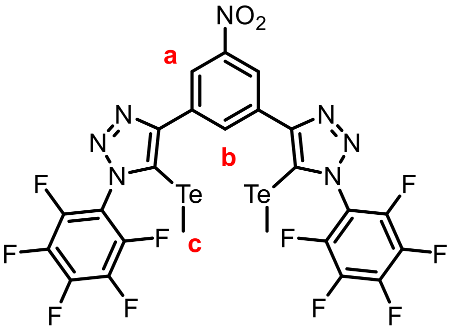

Carrier 1•ChB. Synthesised according to General Procedure 2 from 1,3-diethynyl-5-nitrobenzene (200 mg, 1.17 mmol) to afford the telluromethyl appended alkyne **11**, which was subsequently used immediately in a CuAAC reaction with pentafluorobenzene azide according to General Procedure 1 to afford **1•ChB** (695 mg 0.796 mmol, 68%) as a yellow solid. **^{1}H NMR** (500 MHz, $CDCl_3$) δ 9.36 (t, *J* = 1.6 Hz, $1H_b$), 9.16 (d, *J* = 1.6 Hz, $2H_a$), 2.10 (s, $6H_c$). **^{19}F NMR** (470 MHz, $CDCl_3$) δ -142.72 – -143.58 (m), -147.32 – -14.14 (m), -158.92 – -159.65 (m). **^{13}C NMR** (126 MHz, $CDCl_3$) δ 151.52, 148.94, 143.89 (dm, *J* = 253 Hz), 138.14 (dm, *J* = 267 Hz), 132.80, 132.74, 12262, 113.44 (m), 103.77, -12.55. **HRMS** (ESI+ve) m/z: 875.8832 ($[M+H]^+$, $C_{24}H_{10}O_2N_7F_{10}Te_2$ requires 875.8838).

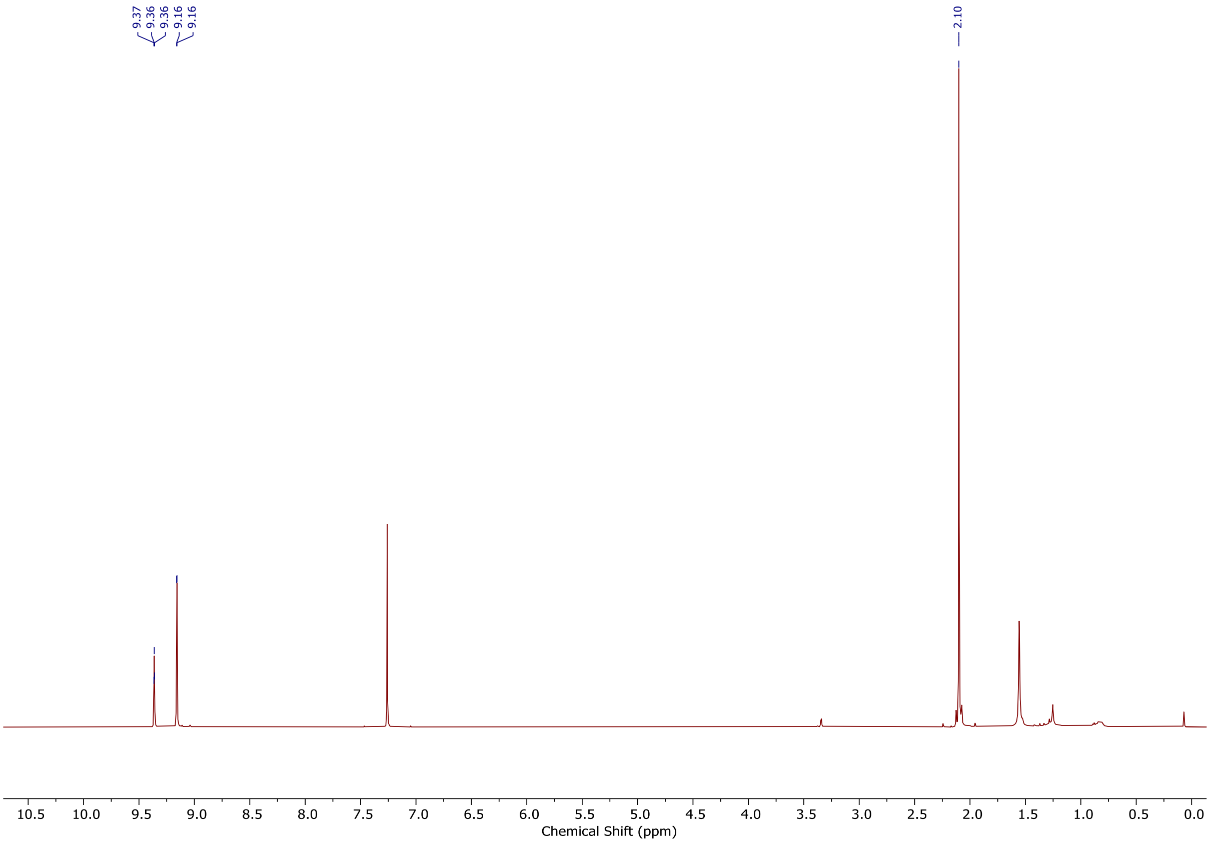

Figure S9. ^{1}H NMR Spectrum of **1•ChB** ($CDCl_3$, 298 K, 500 MHz).

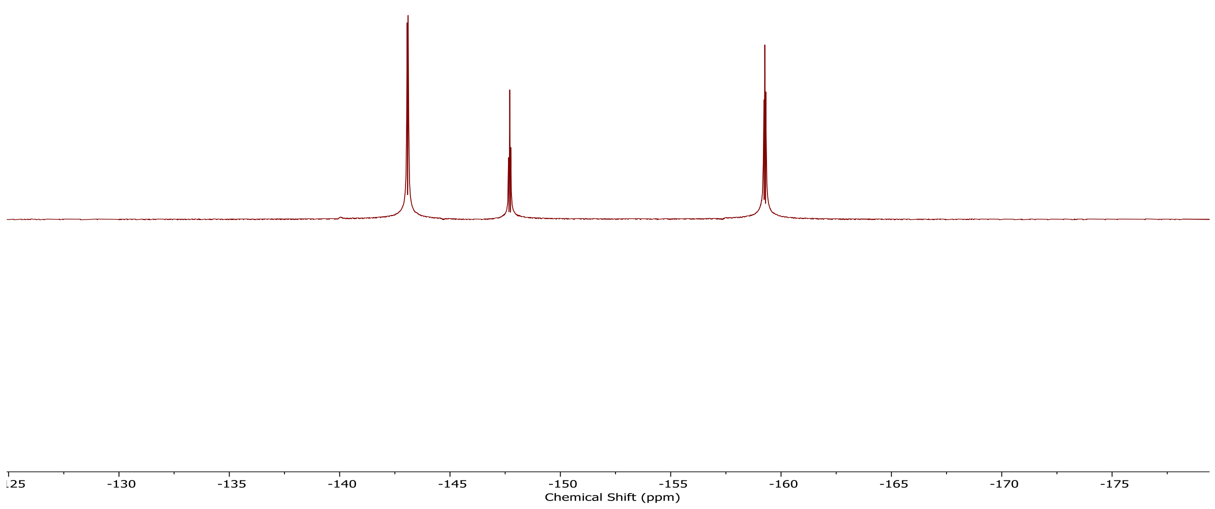

Figure S10. ^{19}F NMR Spectrum of **1•ChB** ($CDCl_3$, 298 K, 476 MHz).

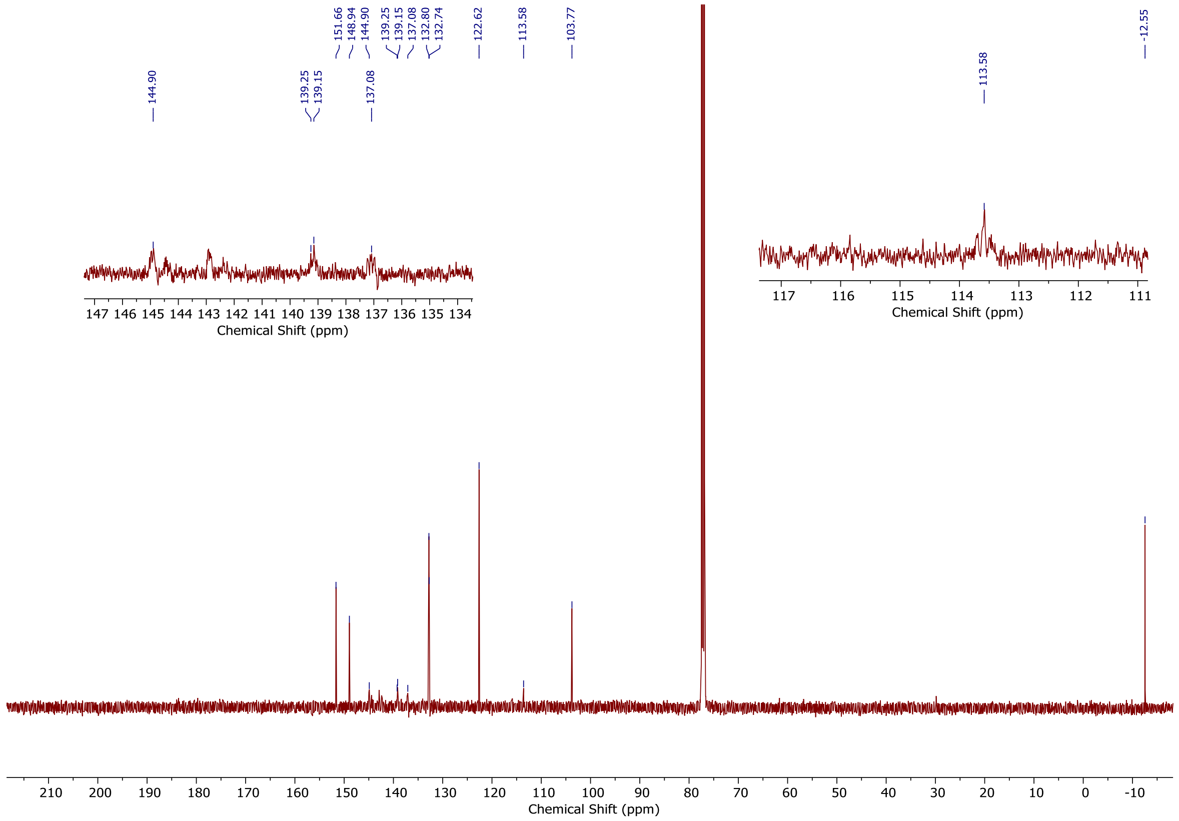

Figure S11. ^{13}C NMR Spectrum of **1•ChB** ($CDCl_3$, 298 K, 476 MHz).

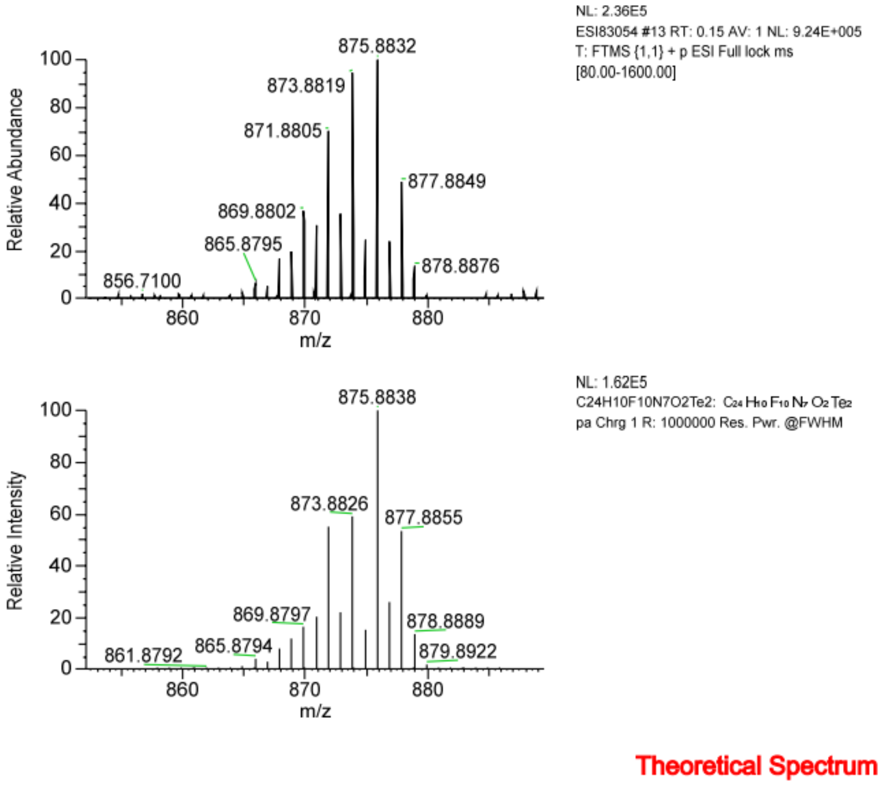

Figure S12. HRESI spectrum of **1•ChB**.

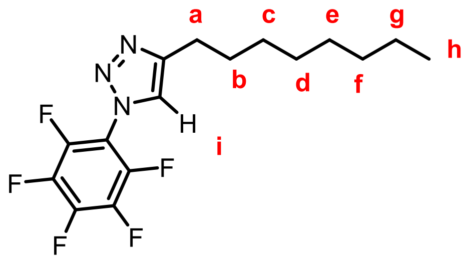

Carrier 2•HB. Synthesised according to General Procedure 1 with 1-decyne (50 mg, 0.362 mmol). The product was purified by column chromatography (30 % ethyl acetate in hexane) to give a white solid (73.2 mg, 0.211 mmol, 46 %). **^{1}H NMR** (400 MHz, $CDCl_3$) δ 7.55 (s, $1H_i$), 2.82 (t, J = 7.7 Hz, $2H_a$), 1.75 (quint, J = 7.5 Hz, $2H_b$), 1.40-1.28 (m, $10H_{c\text{-}g}$), 0.88 (t, J = 6.8 Hz, $3H_h$). **^{19}F NMR** (471 MHz, $CDCl_3$) δ -145.70 (m), -150.67 (m), -159.73 (m). **^{13}C NMR** (126 MHz, $CDCl_3$) δ 149.12, 142.51, 142.19, 138.18, 123.09, 113.41, 31.96, 29.42- 29.25, 25.61, 22.78, 14.21. **HRMS** (ESI+ve) m/z: 348.1491 ($[M+H]^+$, $C_{16}H_{19}N_3F_5$ requires 348.1494)

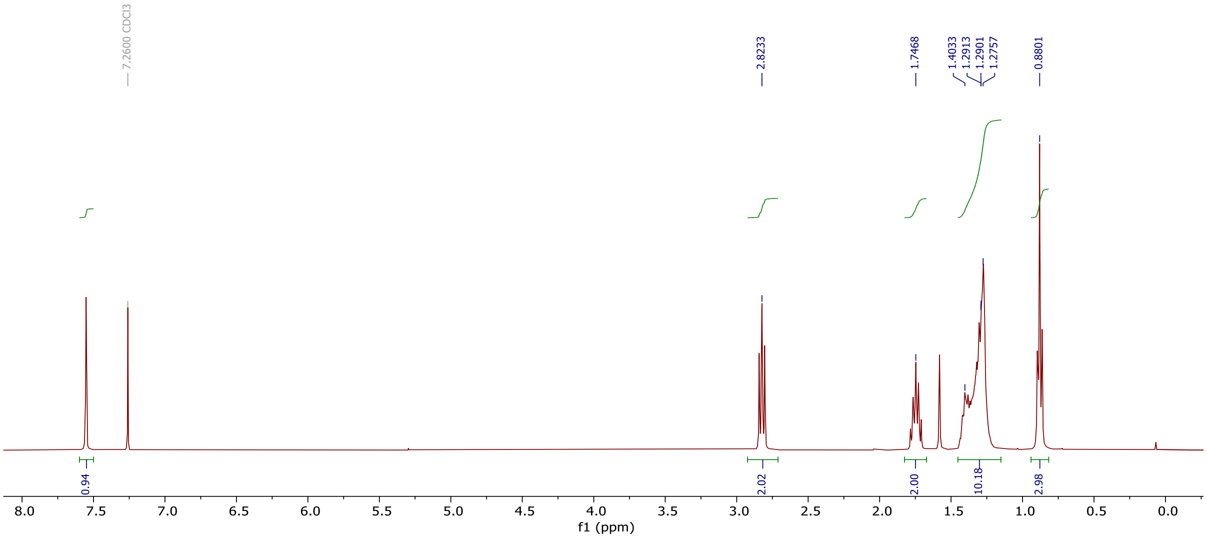

Figure S13. ^{1}H NMR Spectrum of **2•HB** ($CDCl_3$, 298 K, 400 MHz).

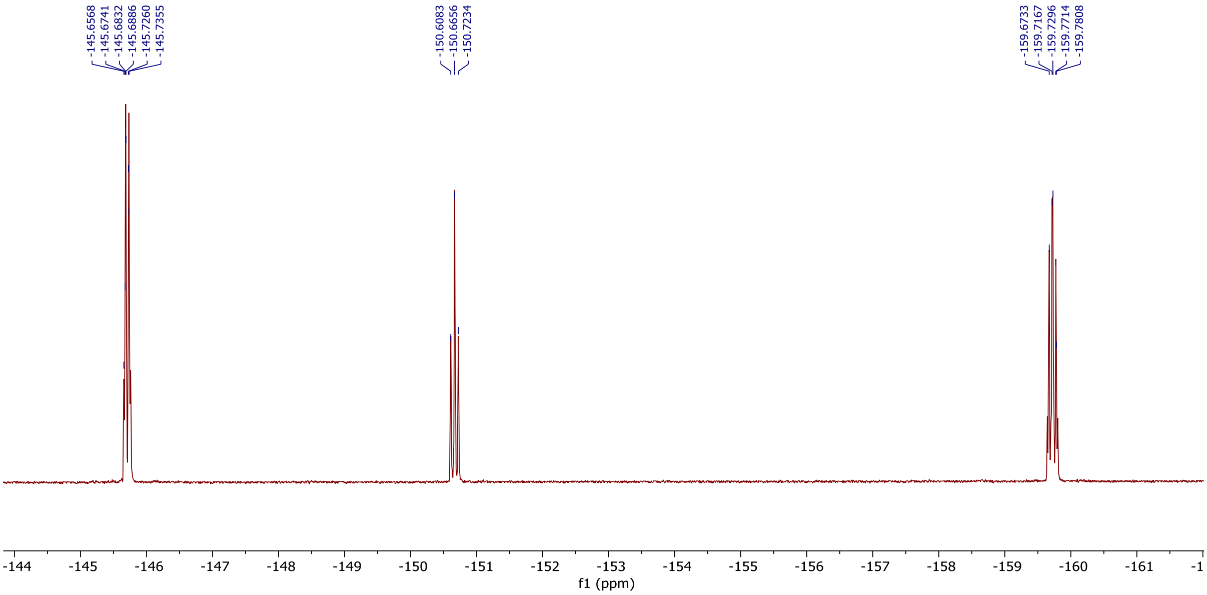

Figure S14. ^{19}F NMR Spectrum of **2•HB** ($CDCl_3$, 298 K, 471 MHz).

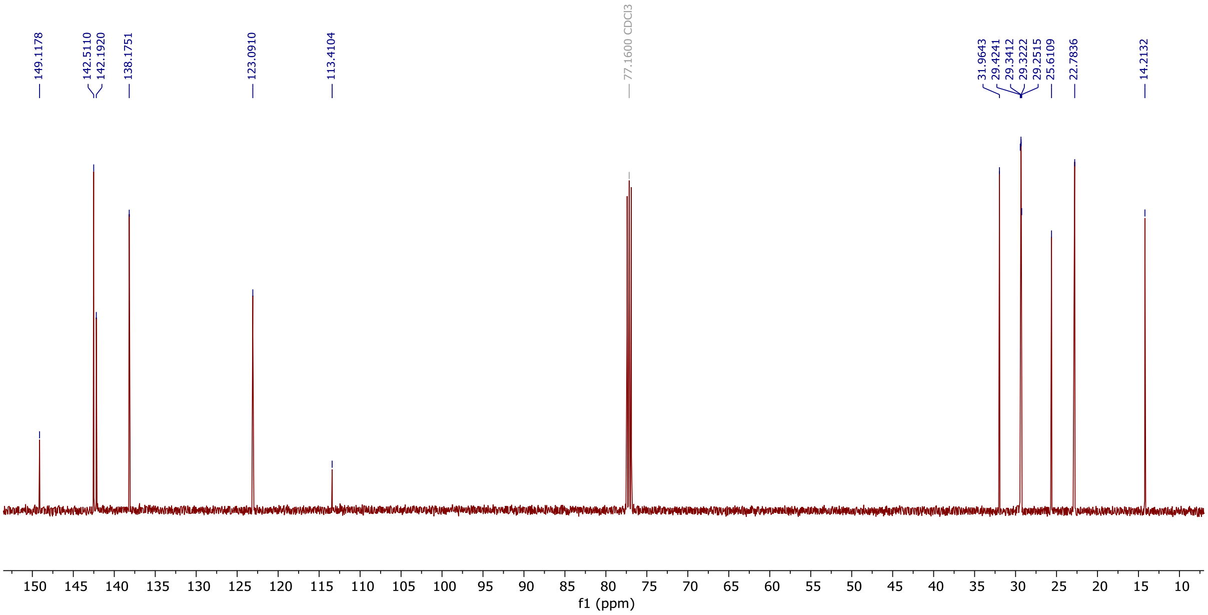

Figure S15. ^{13}C NMR Spectrum of **2•HB** ($CDCl_3$, 298 K, 126 MHz).

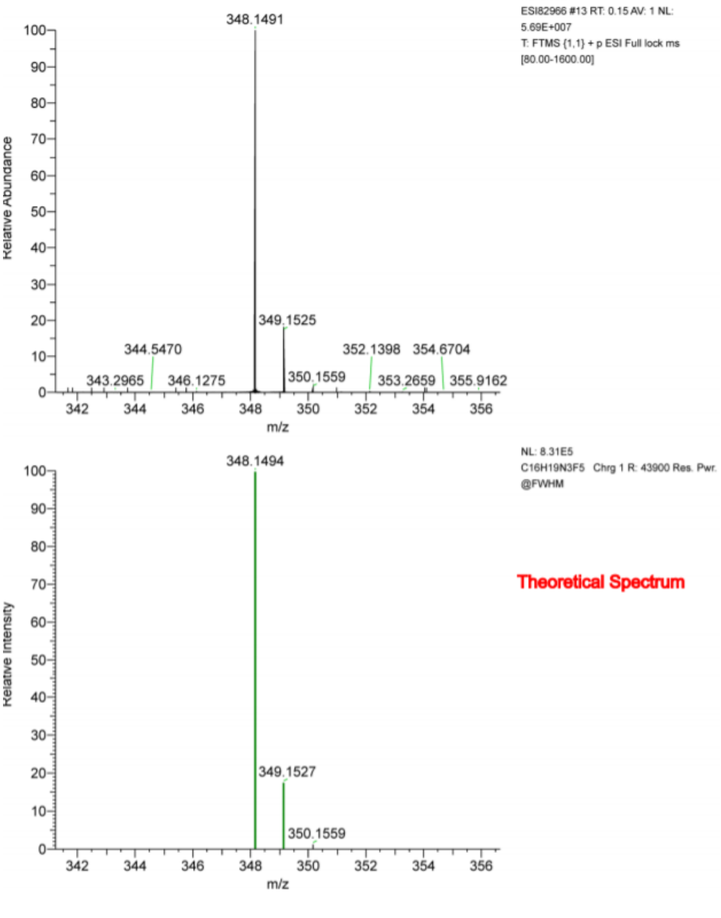

Figure S16. HRESI Spectrum of **2•HB**.

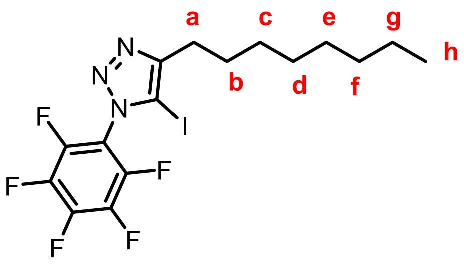

Carrier 2•XB. Synthesised according to General Procedure 1 with iododecyne **6** (120 mg, 0.454 mmol). The product was purified by column chromatography (30 % ethyl acetate in hexane) to give a white solid (43.7 mg, 0.0923 mmol, 49 %). **^{1}H NMR** (400 MHz, $CDCl_3$) δ 2.75 (t, J = 7.7 Hz, $2H_a$), 1.78 (quint, J = 7.5 Hz, $2H_b$), 1.37-1.28 (m, $10H_{c\text{-}g}$), 0.89 (t, J = 6.9 Hz, $3H_h$). **^{19}F NMR** (376 MHz, $CDCl_3$) δ -142.32 – -143.12 (m), -147.98 (tt, J = 21.4, 3.3 Hz), -159.19 – -159.85 (m). **^{13}C NMR** (126 MHz, $CDCl_3$) δ 143.68, 143.33, 138.05, 31.98, 29.39, 29.32, 29.29, 28.87, 26.27, 22.80, 14.25. **HRMS** (ESI+ve) m/z: 474.0457 ($[M+H]^+$ $C_{16}H_{18}N_3F_5I$, requires 474.0460)

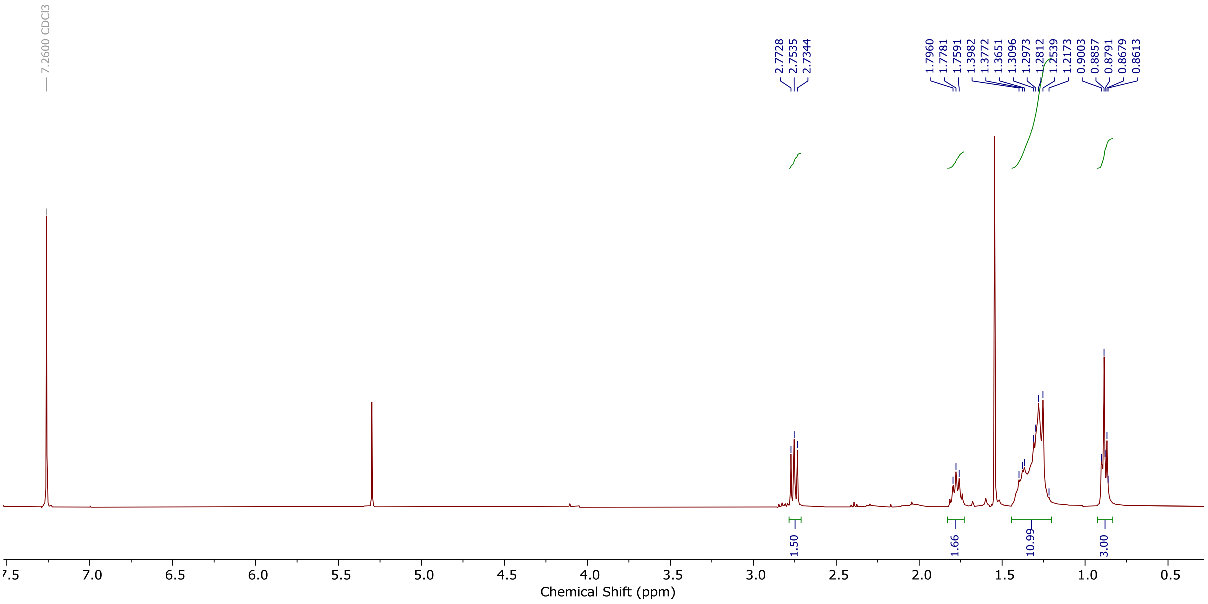

Figure S17. ^{1}H NMR Spectrum **of 2•XB** ($CDCl_3$, 298 K, 400 MHz).

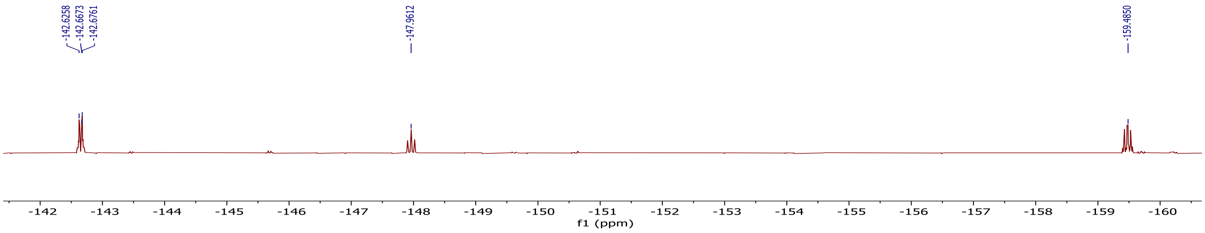

Figure S18. ^{19}F NMR Spectrum of **2•HB** ($CDCl_3$, 298 K, 376 MHz).

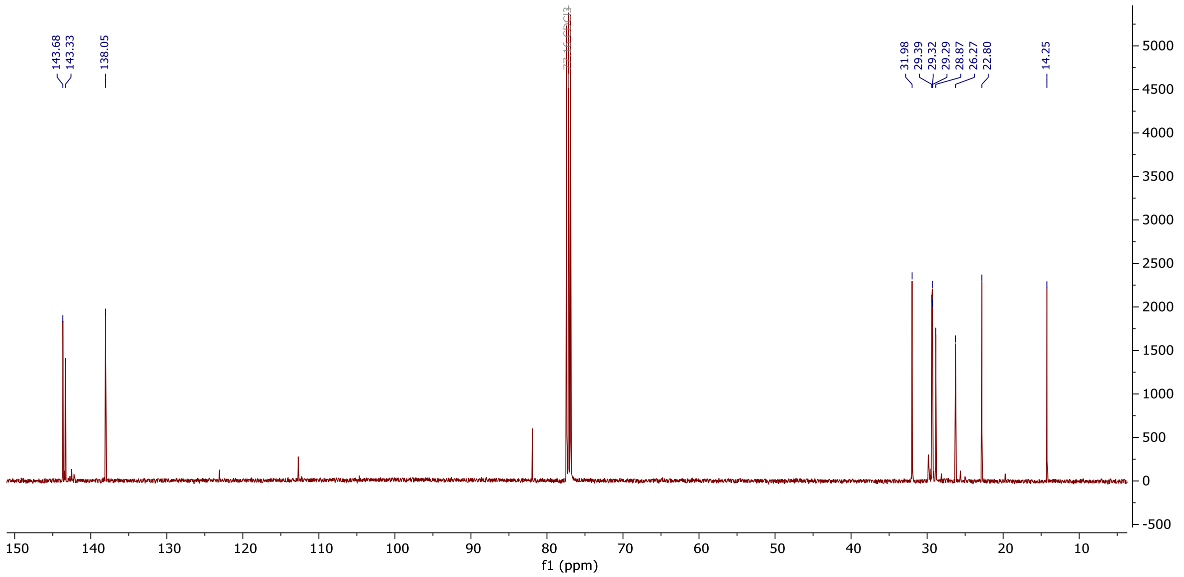

Figure S19. ^{13}C NMR Spectrum of **2•HB** ($CDCl_3$, 298 K, 126 MHz).

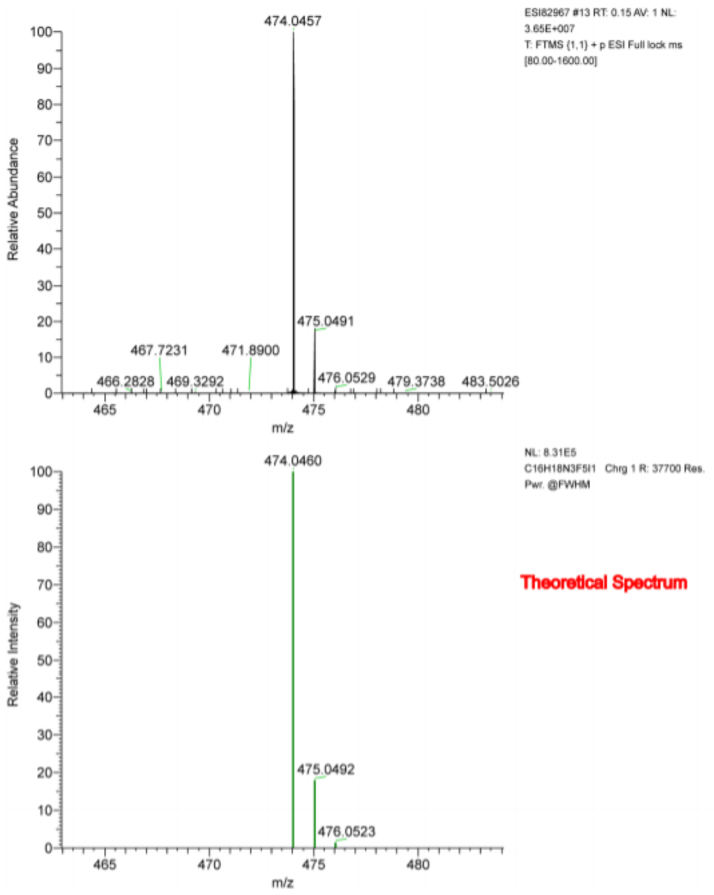

Figure S20. HRESI spectrum of **2•HB**.

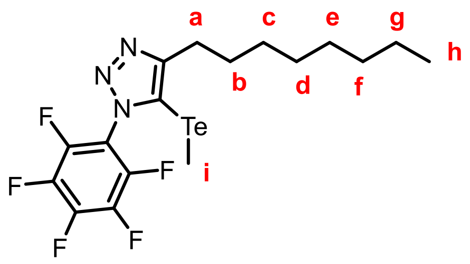

Carrier 2•ChB. Synthesised according to General Procedure 2 from 1-decyne (200 mg, 1.45 mmol) to afford the telluromethyl appended alkyne **8**, which was subsequently used immediately in a CuAAC reaction with pentafluorobenzene azide according to General Procedure 1 to afford **2•ChB** (362 mg 0.740 mmol, 51%) as a yellow solid. **^{1}H NMR** (500 MHz, $CDCl_3$) δ 2.93 – 2.86 (m, $2H_a$), 1.93 (s, $3H_i$), 1.80 (p, *J* = 7.6 Hz, $2H_b$), 1.47 – 1.21 (m, $10H_{c\text{-}g}$), 0.95 – 0.83 (m, $3H_h$). **^{19}F NMR** (470 MHz, $CDCl_3$) δ -143.32 – -143.95 (m), -148.81 – -149.25 (m), -159.66 – -160.40 (m). **^{13}C NMR** (126 MHz, $CDCl_3$) δ 157.62, 143.99 (dm, *J* = 217 Hz), 137.54 (dm, *J* = 268 Hz), 114.22, 104.57, 32.01, 30.01, 29.50, 29.48, 29.35, 27.33, 22.82, 14.25, -13.19, -14.01. **HRMS** (ESI+ve) m/z: 492.0710 ($[M+H]^+$, $C_{17}H_{21}N_3F_5Te$ requires 492.0713)

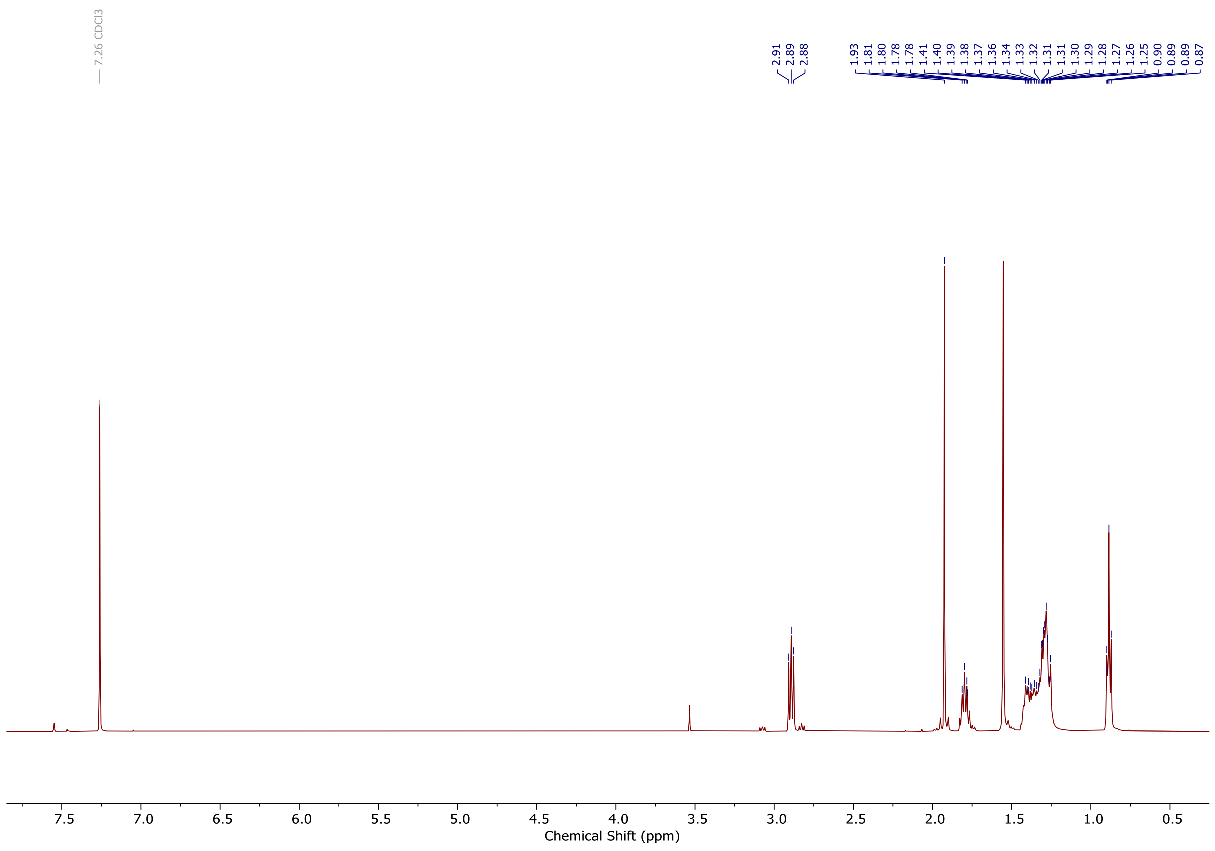

Figure S21. ^{1}H NMR Spectrum of **2•ChB** ($CDCl_3$, 298 K, 500 MHz).

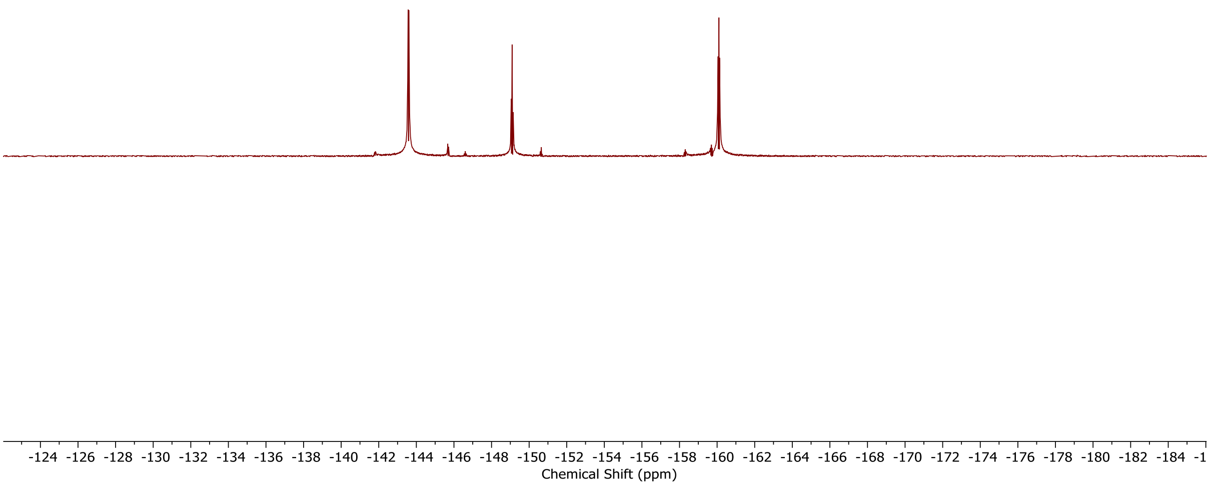

Figure S22. ^{19}F NMR Spectrum of **2•ChB** ($CDCl_3$, 298 K, 470 MHz).

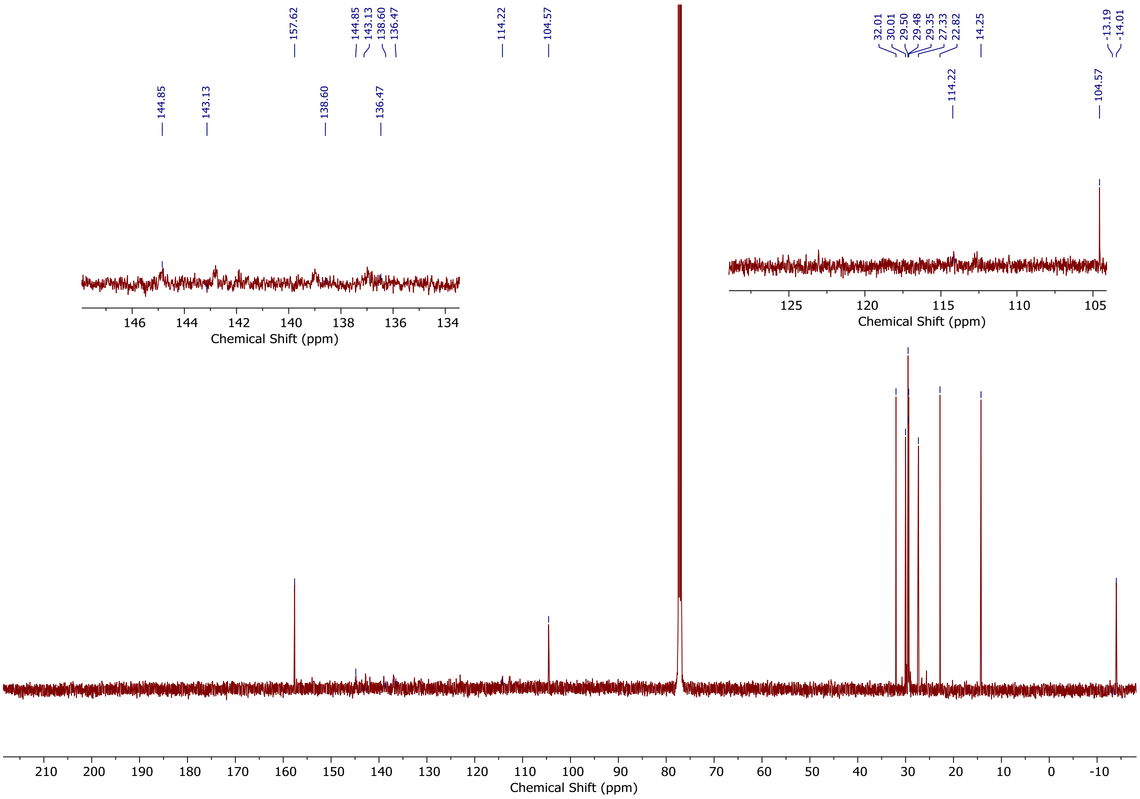

Figure S23. ^{13}C NMR Spectrum of **2•ChB** ($CDCl_3$, 298 K, 126 MHz).

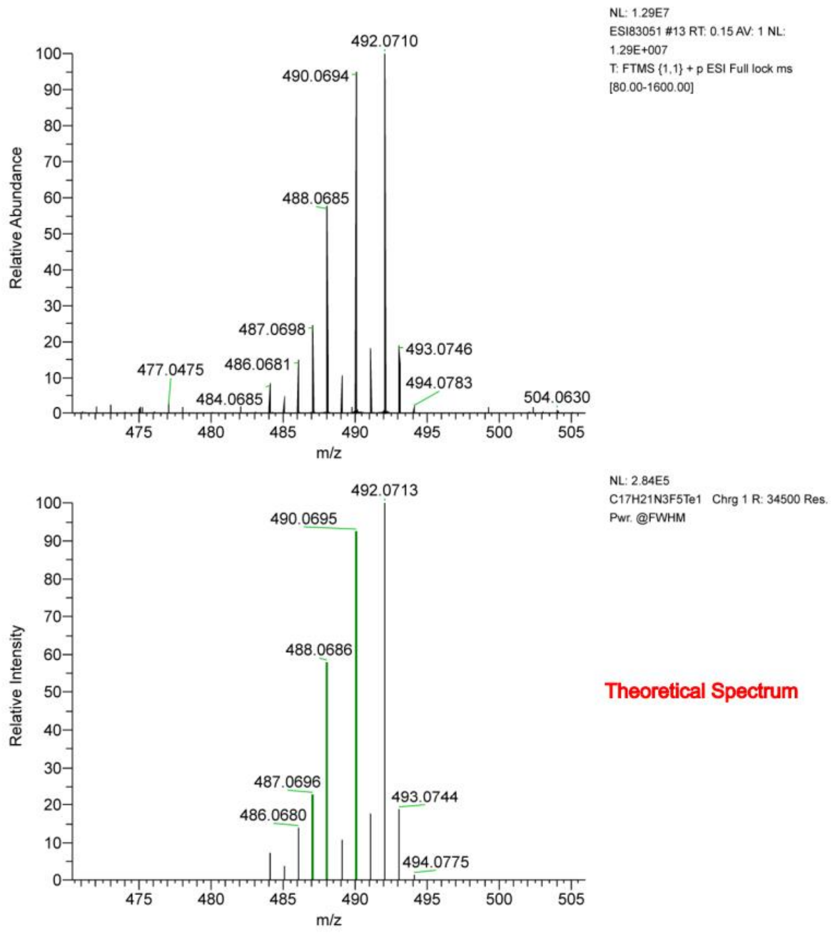

Figure S24. HRESI spectrum of **2•ChB**.

3. **Single crystal X-ray diffraction experiments**

Crystal Structure Determination

Deposition Number(s) 2075766 (for **1•XB**) and 2075767 (for **1•ChB**) contains the supplementary crystallographic data for this paper. These data are provided free of charge by the joint Cambridge Crystallographic Data Centre and Fachinformationszentrum Karlsruhe Access Structures service www.ccdc.cam.ac.uk/structures."

Single-crystal X-ray diffraction intensities for **1•XB** and **1•ChB** were collected at 150 K on Oxford Diffraction/Agilent SuperNovae diffractometers with Cu-Kα (λ = 1.54184 Å) radiation equipped with a nitrogen gas Oxford Cryosystems Cryostream unit.[6] A suitable crystal was chosen and mounted on a 200 μm MiTeGen loop using perfluoropolyether oil. The CrysAlisPro[7] software was used for data collection and integration. All structures were solved using SuperFlip[8] and refined using full-matrix least-squares refinement within the CRYSTALS[9] suite. All non-hydrogen atoms were refined anisotropically. The hydrogen atoms were positioned at geometrically sensible positions and refined using a riding model.

Table S1. Selected Crystallographic and Refinement Data: X-ray Crystal Structures of **1•XB, 1•ChB**.

Compound	**1•XB**	**1•ChB**
Formula	C23 H4 Cl3 F10 I2 N7 O2	C25 H11 Cl2 F10 N7 O2 Te2
Formula Weight	960.48	957.49
a (Å)	14.0124(4)	9.4324(1)
b (Å)	16.5955(3)	14.9145(2)
c (Å)	14.3324(4)	21.8284(3)
α (°)	90	90
β (°)	119.046(3)	99.7769(12)
γ (°)	90	90
Unit cell volume ($Å^3$)	2913.72(15)	3026.21(7)
Crystal system	monoclinic	Monoclinic
Space group	P 21/c	P 21/c
Z	4	4
Temperature (K)	150 K	150 K
Radiation Type	Copper	Copper
λ (Å)	1.54180	1.54180
Reflections (all)	56438	19516
Reflections (unique)	6127	6333
R_{int}	0.068	0.047
R[I > 2σ(I)]	0.0925	0.0593
wR(F^2) (all data)	0.2223	0.1592
S	1.053	1.005

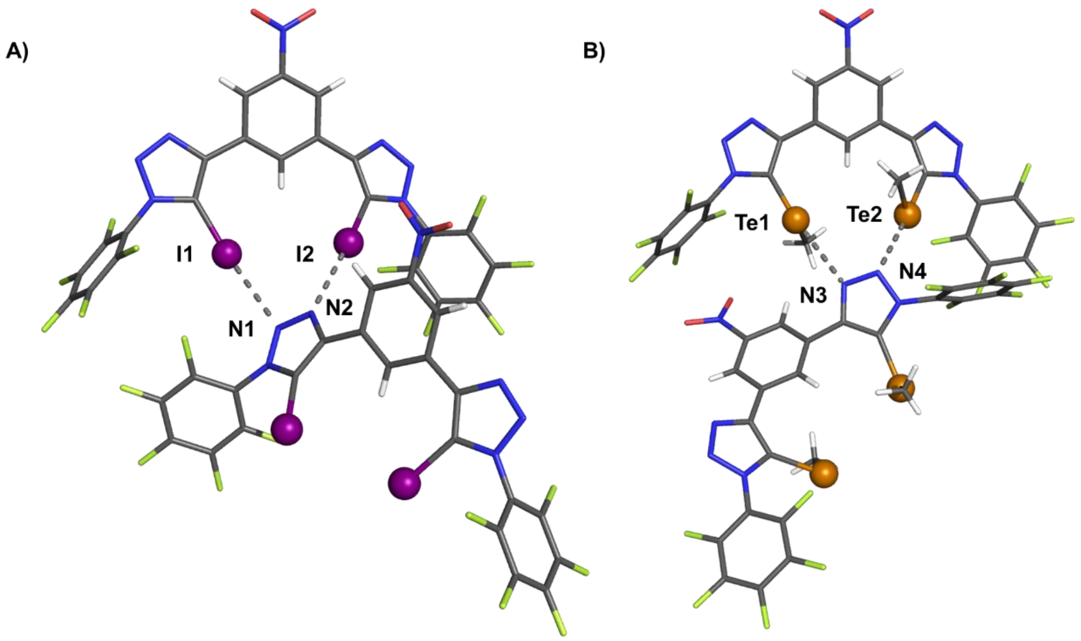

Figure S25. X-ray crystallographic structures of (A) **1•XB**, (B) **1•ChB**, XB and ChB interactions shown as dashed lines. Grey = carbon, blue = nitrogen, white = hydrogen, red = oxygen, green = fluorine, purple = iodine, orange = tellurium.

Table S2. Selected solid state XB and ChB contacts in **1•XB** and **1•ChB**.

Contact	Interatomic Distances (Å)[a]	Angle (°)[a],[b]	%VdW
I1···N1	2.954(5)	172.94(19)	84
I2···N2	3.161(5)	158.45(19)	90
Te1···N3	3.303(4)	176.27(18)	83
Te2···N4	2.982(4)	170.99(15)	91

[a] Errors indicated in parenthesis. [b] Contact angle (in degrees) with respect to the C-X bond. X = I or TeMe.

4. Anion transport experiments

Vesicle preparation

A thin film of lipid (1-palmitoyl-2-oleoyl-*sn*-3-phosphatidylcholine POPC, egg-yolk phosphatidylglycerol EYPG or dipalmitoyl phosphatidylcholine DPPC) was formed by evaporating a chloroform solution under reduced pressure on a rotary evaporator (40 °C) and then under high vacuum for 6 hours. The lipid film was hydrated by vortexing with the prepared buffer (100 mM NaCl, 10 mM HEPES, 1 mM 8-Hydroxypyrene-1,3,6-trisulfonic acid trisodium salt (HPTS), pH 7.0). The lipid suspension was then subjected to 5 freeze-thaw cycles using liquid nitrogen and a water bath (40°C) followed by extrusion 19 times through a polycarbonate membrane (pore size 200 nm). Extravesiclular components were removed by size exclusion chromatography on a Sephadex G-25 column with 100 mM NaCl, 10 mM HEPES, pH 7.0. Final conditions: LUVs (2.5 mM lipid); inside 100 mM NaCl, 10 mM HEPES, 1 mM HPTS, pH 7.0; outside: 100 mM NaCl, 10 mM HEPES, pH 7.0. Vesicles for the sodium gluconate assay were prepared by the same procedure, substituting NaCl for NaGluconate in the buffer solution.

Transport assays with HPTS[10],[11]

In a typical experiment, the LUVs containing HPTS (25 µL, final lipid concentration 31.3 µM) were added to buffer (1950 µL of 100 mM NaCl, 10 mM HEPES, pH 7.0) at 25°C under gentle stirring. A pulse of NaOH (20 µL, 0.5 M) was added at 40 secs to initiate the experiment. At 100 s the test transporter (various concentrations, in 5 µL DMSO) was added, followed by detergent (25 µL of Triton X-100 in 7:1 (v/v) H_2O-DMSO) at 300 secs to calibrate the assay. The fluorescence emission was monitored at λ_{em} = 510 nm (λ_{ex} = 405/460 nm). The fractional fluorescence intensity (I_{rel}) was calculated from equation (S1), where R_t is the fluorescence ratio at time t, R_0 is the fluorescence ratio at time 0, and R_d is the fluorescence ratio after the addition of detergent.

$$I_{rel} = \frac{R_t - R_0}{R_d - R_0} \qquad \text{(S1)}$$

The fractional fluorescence intensity (I_{rel}) at 288 s just prior to lysis, defined as the fractional activity y, was plotted as a function of the ionophore concentration (x / µM). Hill coefficients (n) and EC_{50} values were calculated by fitting to the Hill equation (S2):

$$y = y_0 + (y_{max} - y_0).\frac{x^n}{EC50^n + x^n} \qquad \text{(S2)}$$

where y_0 is the fractional activity in the absence of transporter, $y_{\max}$ is the fractional activity in with excess transporter, x is the transporter concentration in the cuvette. Hill plots were fitted to at-least 8, and up to 13 data points spanning the required concentration range, and each individual concentration was repeated at-least twice and averaged.

Experiments with DPPC lipids were conducted in the same way. For elevated temperature studies, the buffer was equilibrated at 45°C (using the Peltier temperature controller) for 5 minutes prior to initiating the experiment. Cation selectivity experiments were conducted by external ion exchange, by adding the POPC vesicle solution (prepared as above) to buffer (100 mM M^+Cl^-, 10 mM HEPES, pH 7.0), where M^+ = Li^+, Na^+, K^+, Rb^+.

Experiments with NMDG-Cl (100 mM) in place of NaCl in both the external and internal buffer was also carried out according to the above procedures, except that the POPC lipid concentration was increased to 0.1 mM and NMDG (20 µL of 0.5 M giving a final concentration 5 mM in the cuvette) was used for the base pulse in place of NaOH. This assay was carried out with and without the addition of the proton channel Gramicidin D (0.1 mol%, added in 4 µL DMSO), which when added was done so 30 seconds after the base pulse.

HPTS assay data for all transporters

In the following figures: Left: change in relative fluorescence intensity over time in the HPTS assay (LUVs (2.5 mM lipid); inside 100 mM NaCl, 10 mM HEPES, 1 mM HPTS, pH 7.0; outside: 100 mM NaCl, 10 mM HEPES, pH 7.0). Right: dependence of the fractional transport activity *y* in the HPTS assay on the concentration of transporter (black squares), and fitted to the Hill equation (red line).

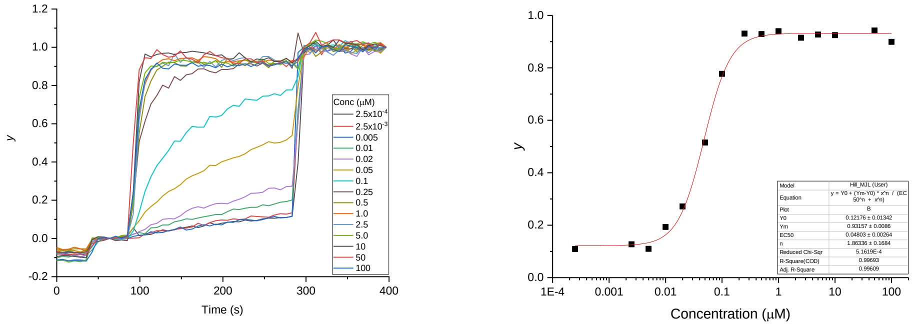

Figure S26. HPTS assay data for carrier **1•HB**.

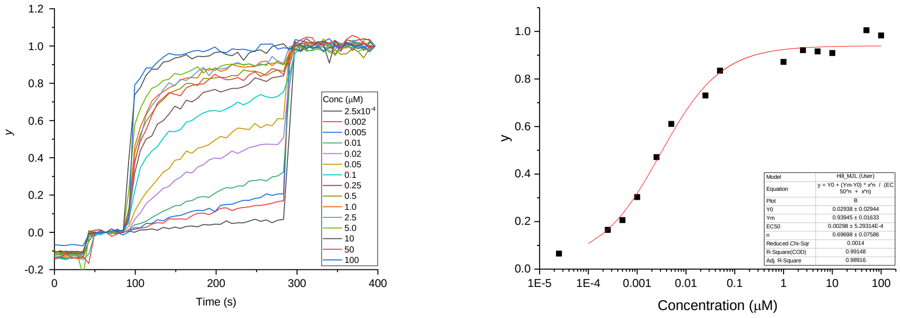

Figure S27. HPTS assay data for carrier **1•XB**.

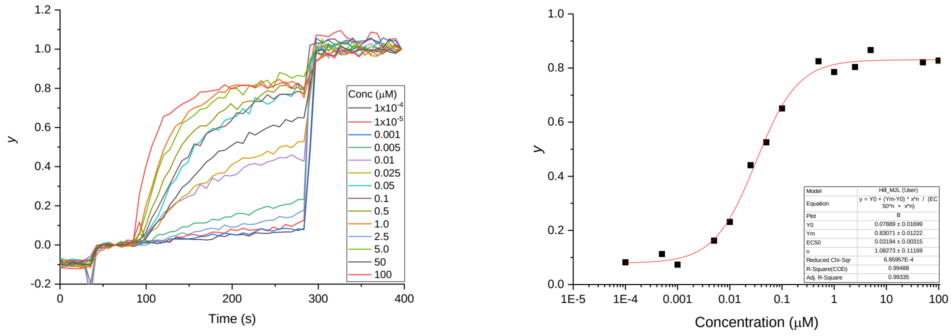

Figure S28. HPTS assay data for carrier **1•ChB**.

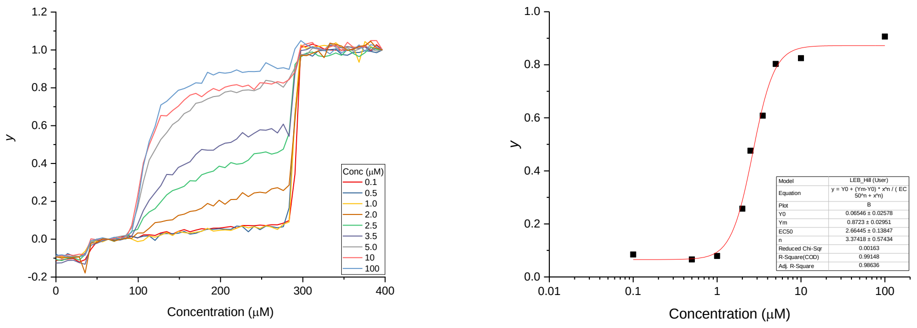

Figure S29. HPTS assay data for carrier **2•HB**.

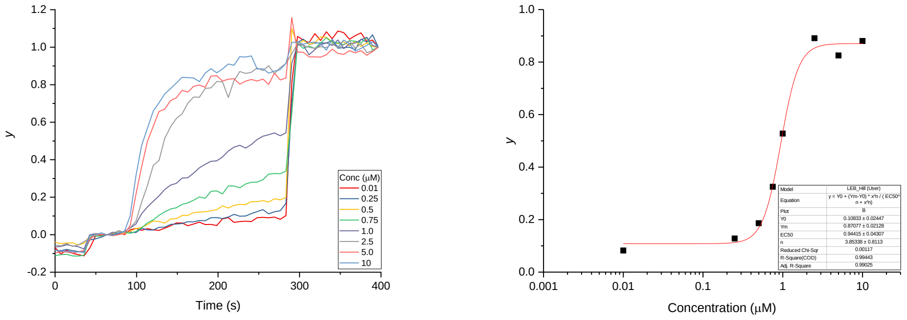

Figure S30. HPTS assay data for carrier **2•XB**.

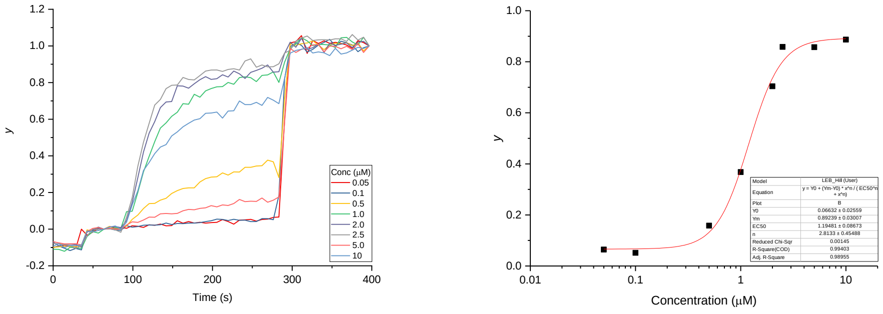

Figure S31. HPTS assay data for carrier **2•ChB**.

Cation Selectivity Studies

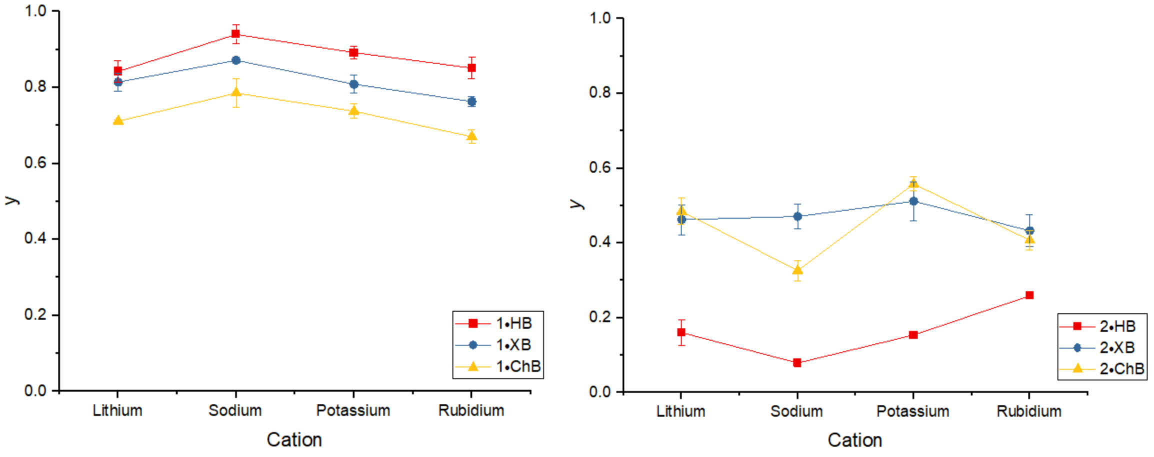

Figure S32. The fractional activity (y) obtained from the HPTS assay with exchange of the external cation (external buffer 100 mM M^+Cl^-, 10 mM HEPES). All carriers were added at 1 µM. Left: bidentate carriers, Right: monodentate carriers. Error bars represent 2 s.d.

NMDG Assay

In the following figures: Left: change in relative fluorescence intensity over time in the NMDG assay (LUVs (0.1 mM lipid); inside 100 mM NMDG-Cl, 10 mM HEPES, 1 mM HPTS, pH 7.0; outside: 100 mM NMDG-Cl, 10 mM HEPES, pH 7.0). Right: dependence of the fractional transport activity *y* in the HPTS assay on the concentration of transporter (black squares) and fitted to the Hill equation (red line). This assay was carried out with and without the addition of Gramicidin D (0.1 mol%, 4 µL DMSO solution) added after the NMDG base pulse (20 µL of 0.5 M) when used. Error bars represent 2 s.d. This assay uses gramicidin as a selective proton channel for the purposes of comparison with previous studies, and to avoid competing interactions between the anionophores and mobile protonophores such as FCCP and CCCP.[12]

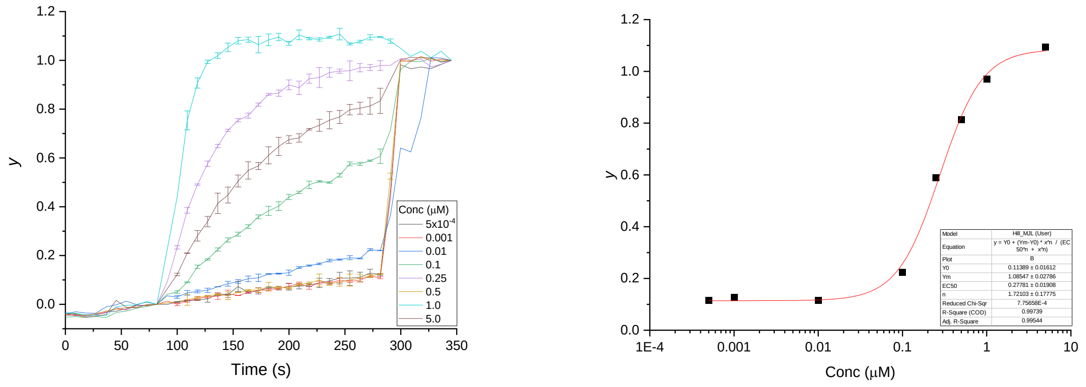

Figure S33. NMDG assay for carrier **1•HB** without gramicidin.

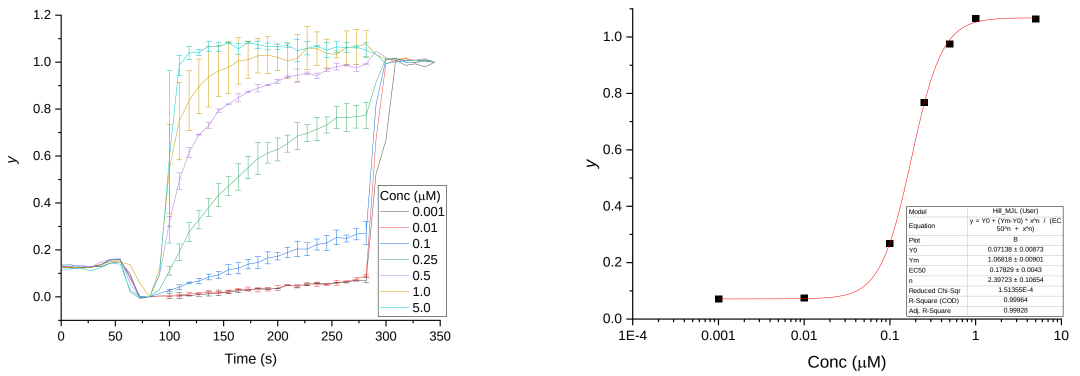

Figure S34. NMDG assay for carrier **1•HB** with gramicidin.

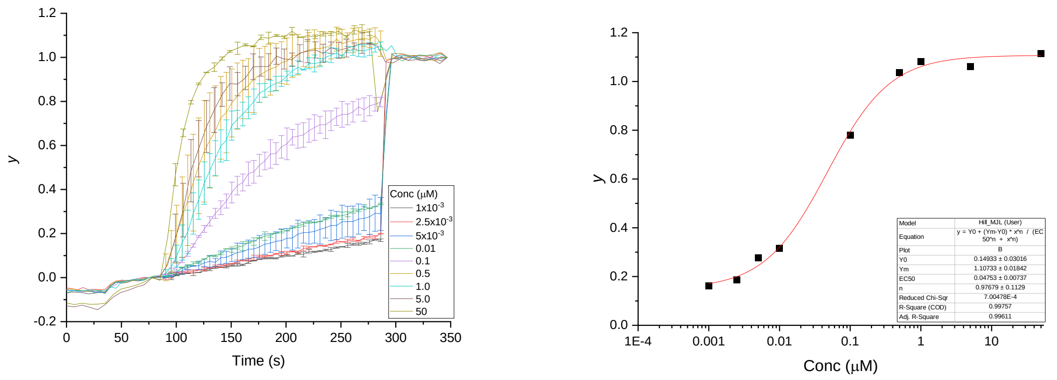

Figure S35. NMDG assay for carrier **1•XB** without gramicidin.

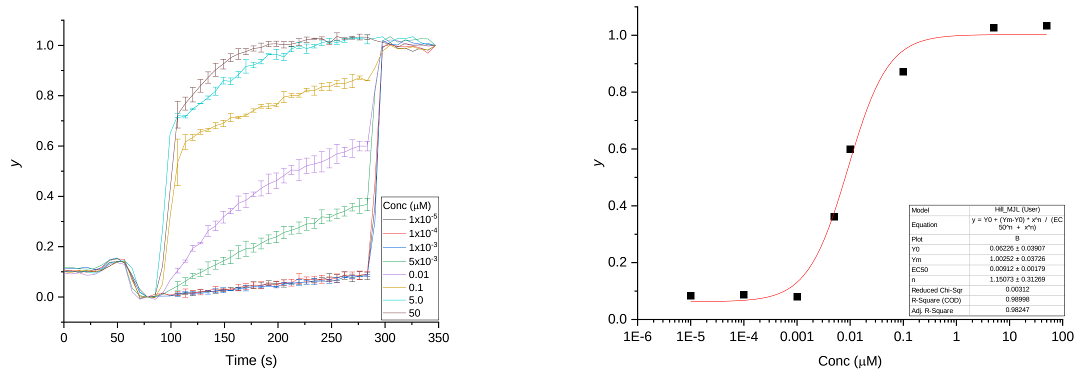

Figure S36. NMDG assay for carrier **1•XB** with gramicidin.

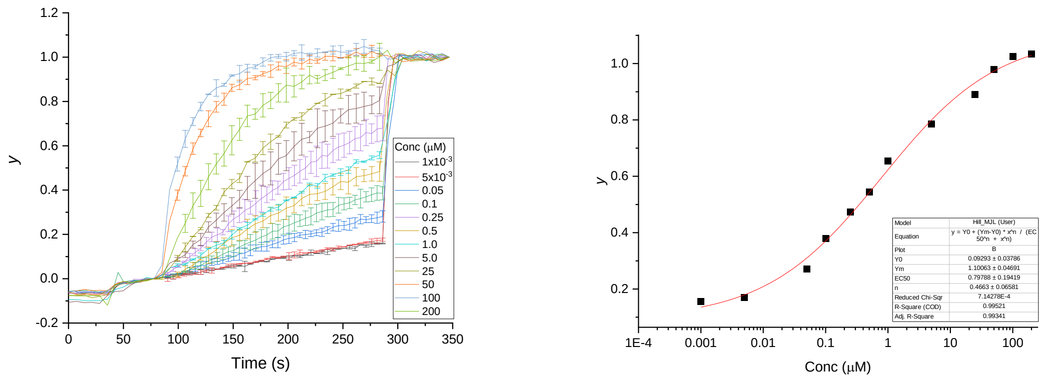

Figure S37. NMDG assay for carrier **1•ChB** without gramicidin.

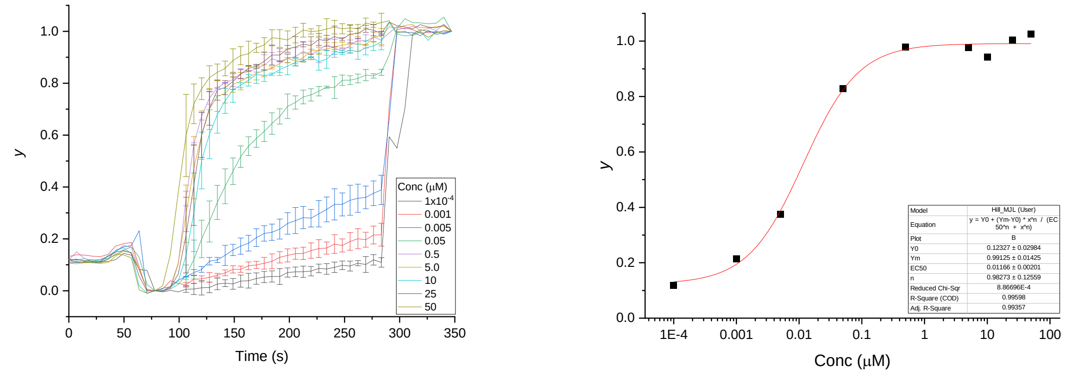

Figure S38. NMDG assay for carrier **1•ChB** with gramicidin.

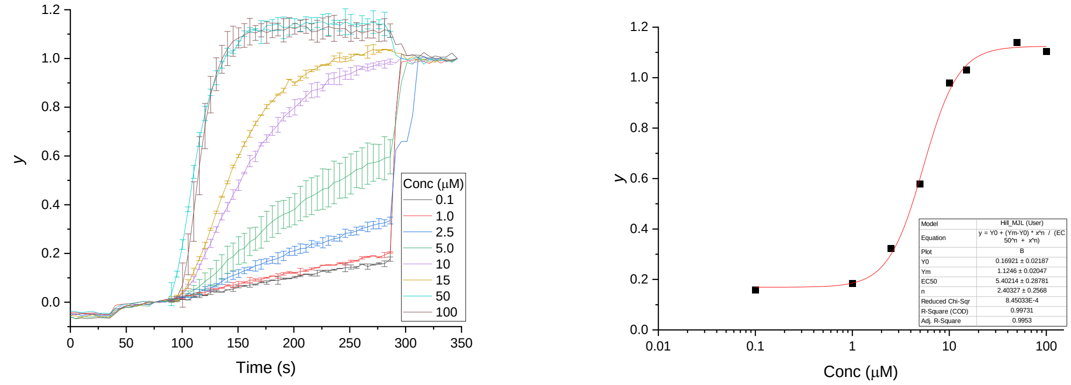

Figure S39. NMDG assay for carrier **2•XB** without gramicidin.

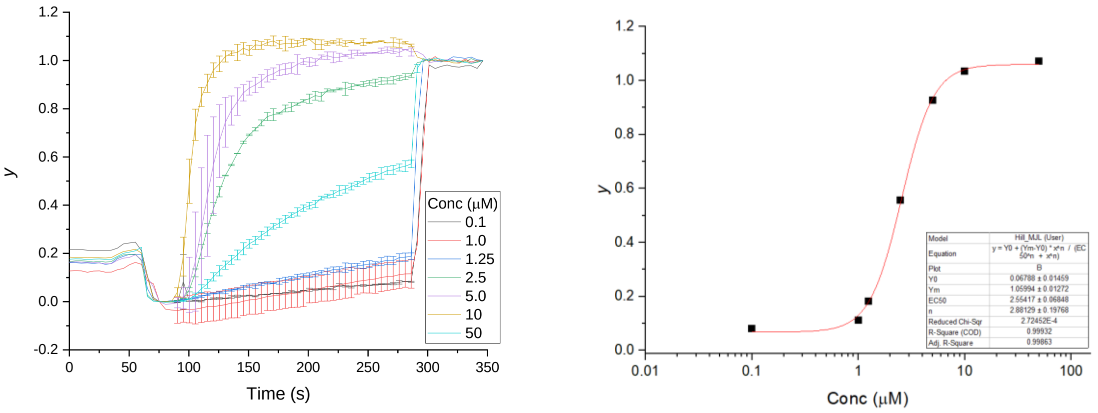

Figure S40. NMDG assay for carrier **2•XB** with gramicidin.

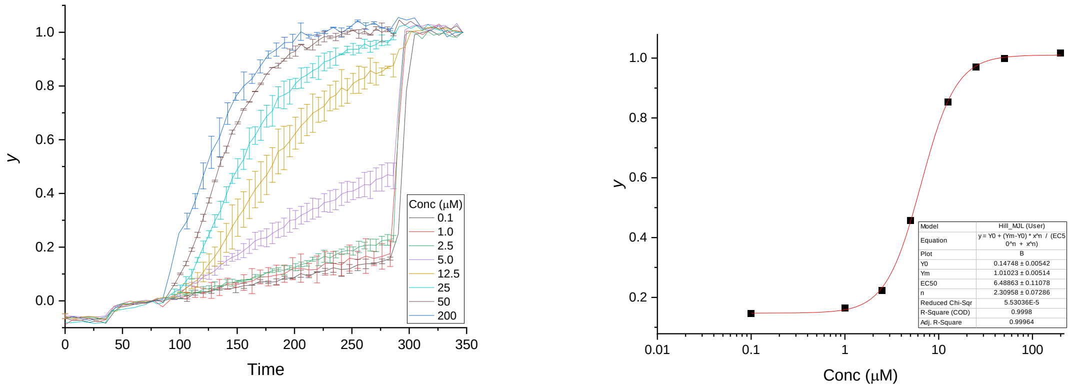

Figure S41. NMDG assay for carrier **2•ChB** without gramicidin.

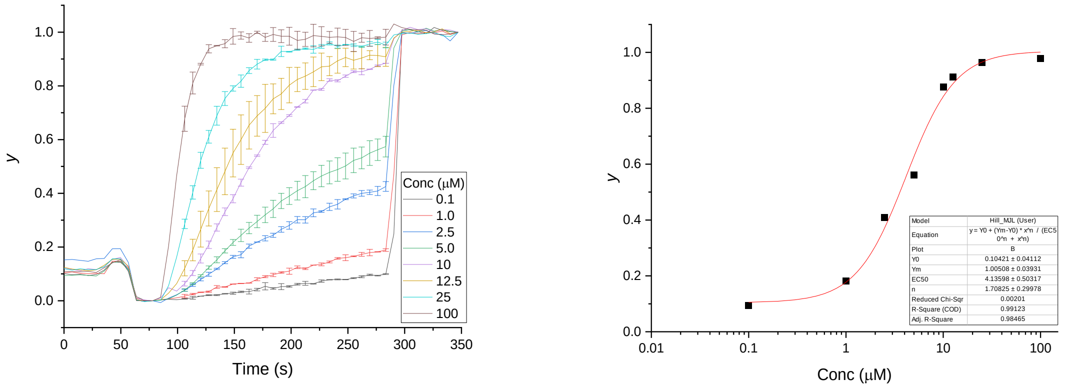

Figure S42. NMDG assay for carrier **2•ChB** with gramicidin.

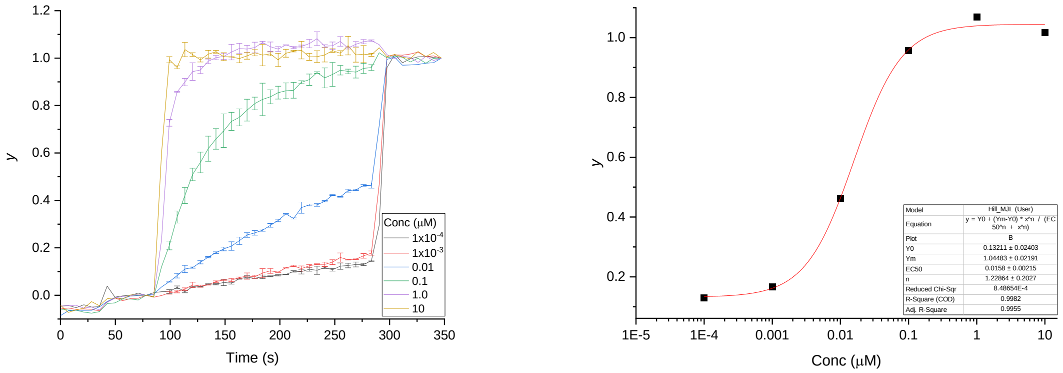

Figure S43. Carrier **3** without gramicidin.

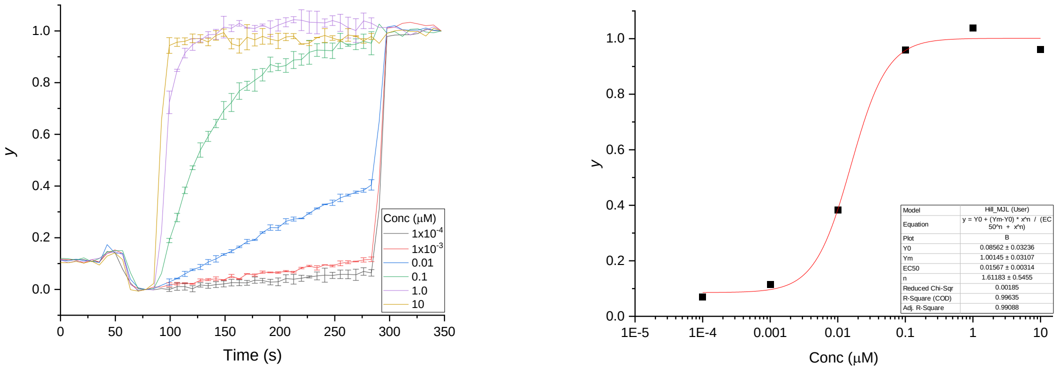

Figure S44. Carrier **3** with gramicidin.

Anionic lipid transport studies

(a)

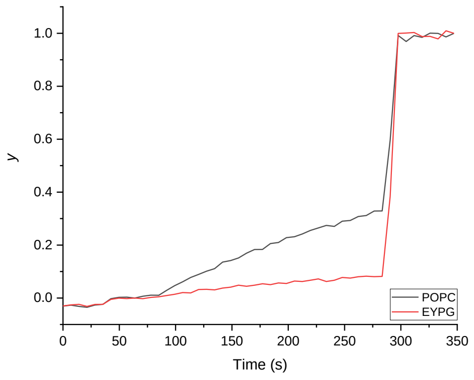

(b)

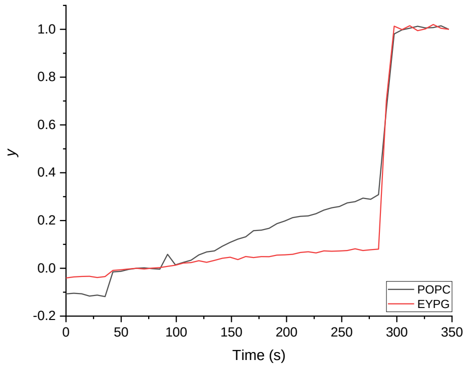

(c)

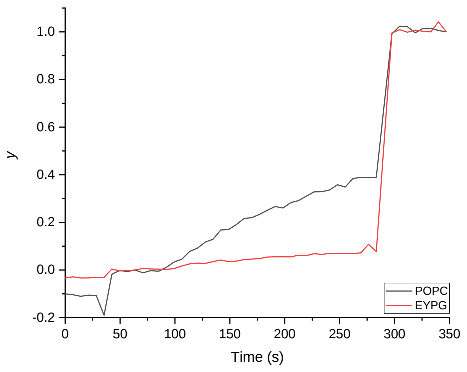

(d)

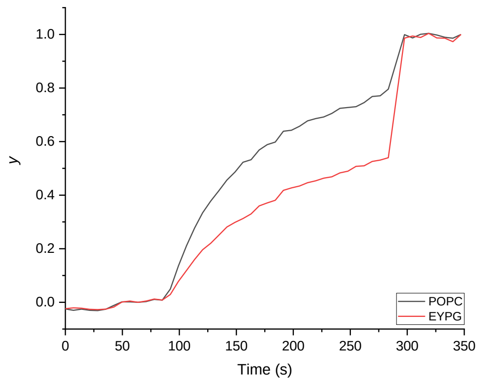

(e)

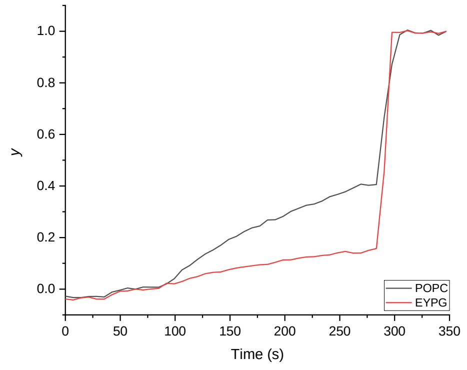

(f)

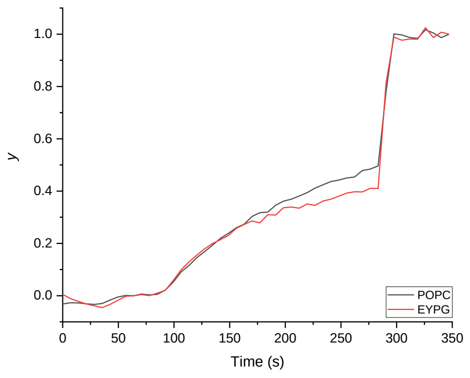

Figure S45. The change in relative fluorescence intensity over time in the HPTS assay utilizing both POPC and EYPG LUVs. Carriers were administered at their EC_{50} values: (a) **1•HB** (48 nM), (b) **1•XB** (3 nM), (c) **1•ChB** (32 nM), (d) **2•HB** (2.7 µM), (e) **2•XB** (1 µM), (f) **2•ChB** (1.2 µM). Reduction in transport when using negatively charged EYPG LUVs supports the conclusion of a Cl^-/OH^- antiporter mechanism.

Gluconate Assay

(a)

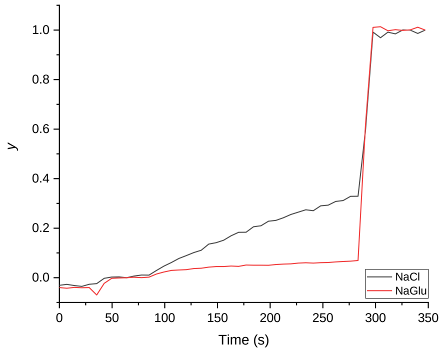

(b)

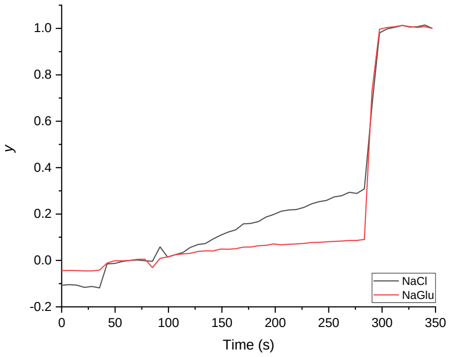

(c)

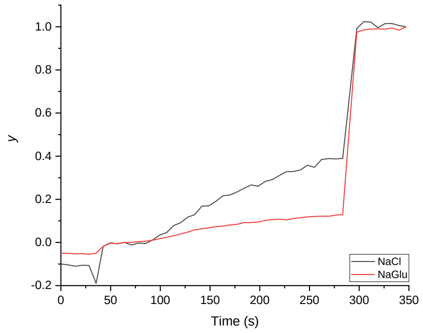

(d)

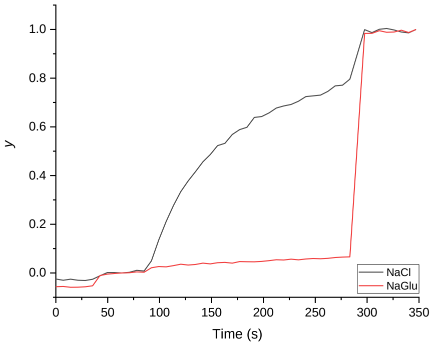

(e)

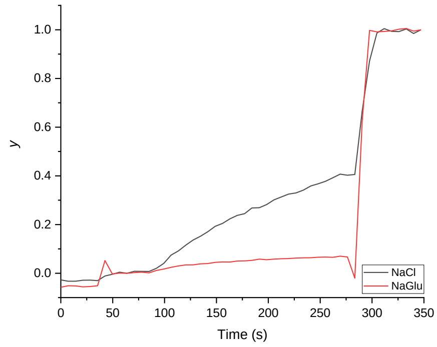

(f)

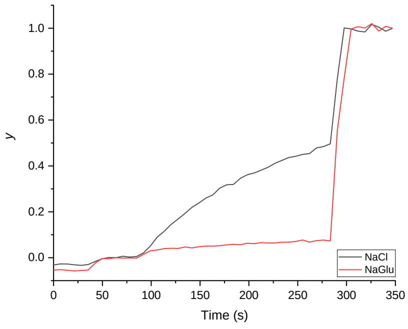

Figure S46. The time-dependent change in fluorescence intensity within the gluconate variation of the HPTS assay. Carriers were administered at their EC_{50} values. (a) **1•HB** (48 nM), (b) **1•XB** (3 nM), (c) **1•ChB** (32 nM), (d) **2•HB** (2.7 µM), (e) **2•XB** (1 µM), (f) **2•ChB** (1.2 µM). The decrease in transport with NaGlu for all carriers indicated they do not facilitate sodium cation transport (H^+/Na^+ antiport), and must therefore operate via an anion transport mechanism.

Carboxyfluorescein leakage assay

POPC vesicles were prepared containing 5(6)-Carboxyfluorescein (CF)[13] (Internal buffer: 10 mM NaCl, 10 mM HEPES, 50 mM CF, pH 7.0; external buffer: 107 mM NaCl, 10 mM HEPES, pH 7.0). Each transport experiment was carried out as follows: the CF-containing POPC vesicles (25 µL, 2.5 mM) were suspended in the external buffer (1995 µL, 107 mM NaCl, 10 mM HEPES, pH 7.0) at 25°C and gently stirrer. At 50 s, the carriers were administered at 1 µM in DMSO (5 µM). The assay was calibrated at 250 s with detergent (40 µL of Triton X-100 in 7:1 (v/v) H_2O-DMSO). The time-dependent change in fluorescence intensity (λ_{ex} = 492 nm, λ_{em} = 517 nm) was monitored, and normalised according to equation S3:

$$I_{rel} = \frac{I_t - I_0}{I_{max} - I_0} \qquad \text{(S3)}$$

where $I_0 = I_t$ before transporter addition, $I_{max} = I_t$ after lysis.

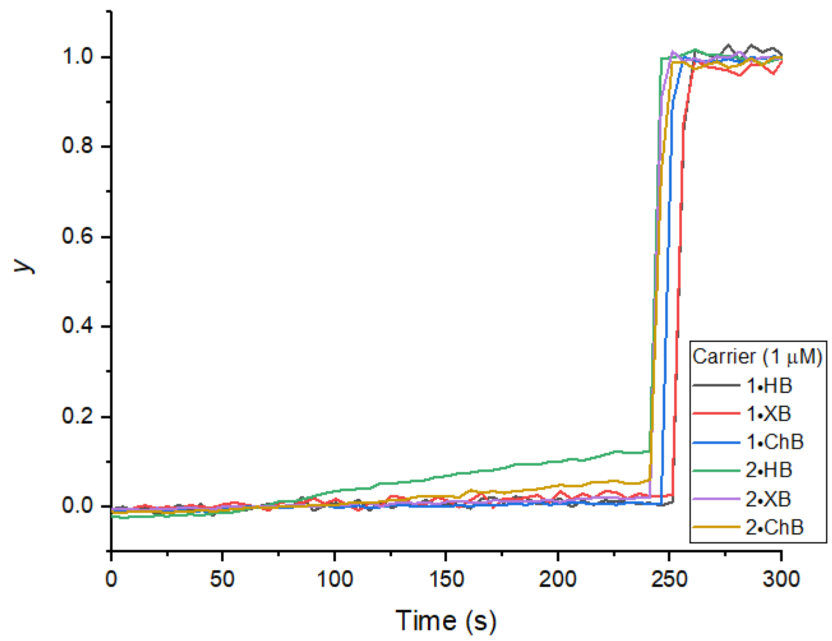

Figure S47. Change in relative fluorescence intensity over time in the CF assay (POPC LUVs (2.5 mM lipid); inside 100 mM NaCl, 10 mM HEPES, 1 mM HPTS, pH 7.0; outside: 100 mM NaCl, 10 mM HEPES, pH 7.0). Carriers added at 1µM.

Membrane fluidity studies

(a)

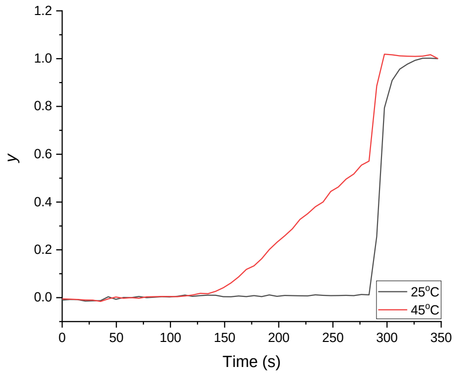

(b)

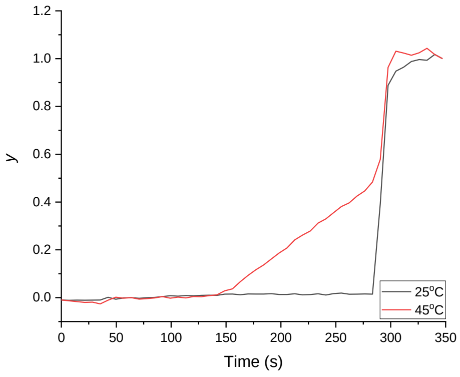

(c)

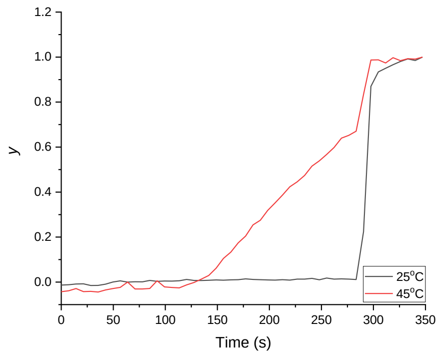

(d)

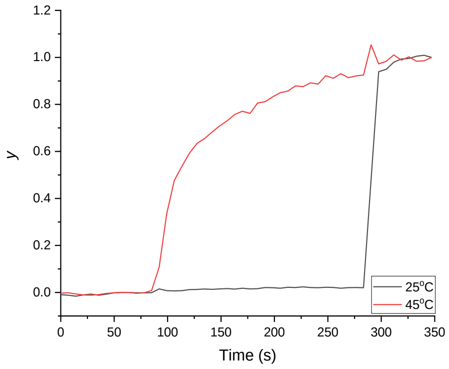

(e)

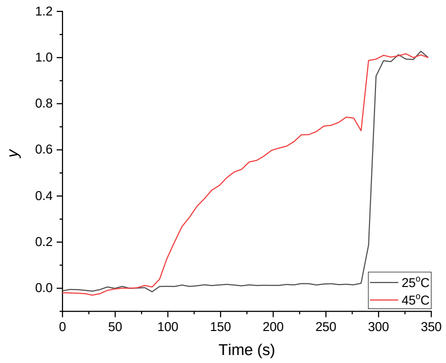

(f)

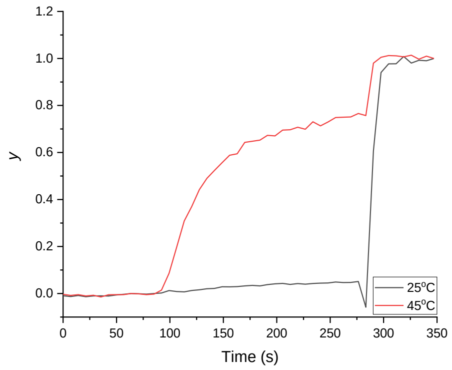

Figure S48. The change in relative fluorescence intensity over time in the HPTS assay utilizing DPPC LUVs at 25°C and 45°C. The temperature of the sample was controlled using a Peltier temperature controller. Carriers were administered at their EC_{50} values. (a) **1•HB** (48 nM), (b) **1•XB** (3 nM), (c) **1•ChB** (32 nM), (d) **2•HB** (2.7 µM), (e) **2•XB** (1 µM), (f) **2•ChB** (1.2 µM). Lack of transport in the gel phase at 25°C, and restoration above the phase transition temperature (41°C) at 45°C, is consistent with a mobile carrier transport mechanism.

5. NMR titration experiments

All binding constants were measured by ^{1}H NMR titrations in a Bruker AVIII 500 spectrometer at 500 MHz and 298 K. The host (anionophores **1** and **2**) was dissolved in acetone-d_6, or 2.5% D_2O-acetone-d_6 (v/v) mixtures, at 1 mM concentration and a known volume (0.5 mL) added to the NMR tube. Known volumes of anion guest (added as the TBA salt) in acetone-d_6, or 2.5% D_2O-acetone-d_6 (v/v) mixtures, were added and the spectra were recorded after each addition. The chemical shift perturbations of the host spectra were monitored as a function of guest concentration. In the case of **1•XB**, **1•ChB** and **1•HB** the internal aryl signal *a* was monitored, in the case of **2•XB**, **2•ChB** and **2•HB;** the methylene signal *b*, the telluromethyl signal *c* and the C-H triazole signal *d,* respectively were monitored (Figure S49). The data was analysed using a global fit procedure using the Bindfit[14],[15] program, using non-linear least squares analysis to obtain the best fit between observed and calculated chemical shifts for the 1:1 binding stoichiometry. In all experiments the association of guest and host was fast on the NMR timescale and the chloride association constants are summarised in **Error! Reference source not found.3.**

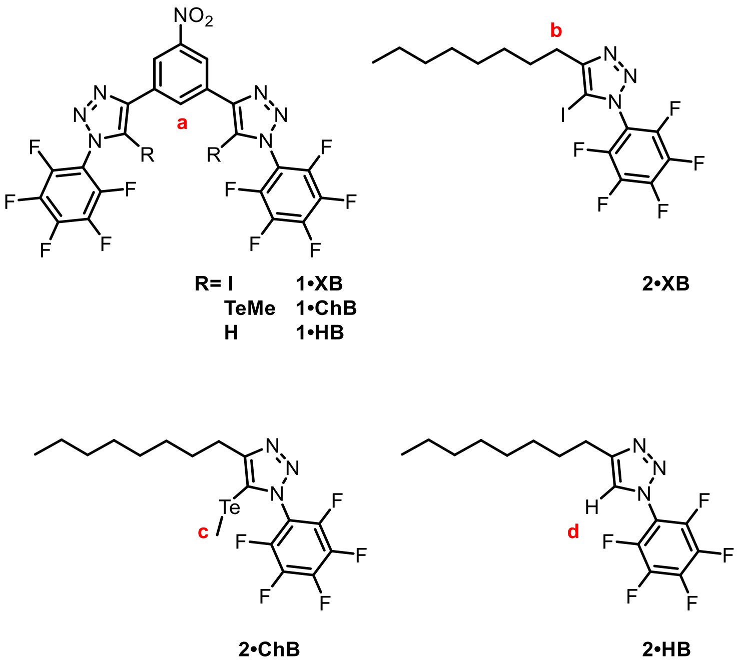

Figure S49. The structures of the bidentate and monodentate carriers with perturbations of the labelled proton signal used for fitting to determine chloride anion association constants.

Table S3. Anion association constants determined from ^{1}H NMR titration experiments.

Chloride Association Constants K_a [M^{-1}] [a]						
Solvent	**1•XB**	**2•XB**	**1•ChB**	**1•ChB**	**1•HB**	**2•HB**
acetone-d_6	>10^5	440	18,543	66	1012	18
2.5% D_2O acetone-d_6[b]	6073	-[c]	156	-[c]	-[d]	-[c]

[a] Association constants calculated using Bindfit, errors (±) less than 7%, chloride added as the TBA salt. [b] 2.5% D_2O-acetone-d_6 (v/v). [c] Not performed. [d] No binding.

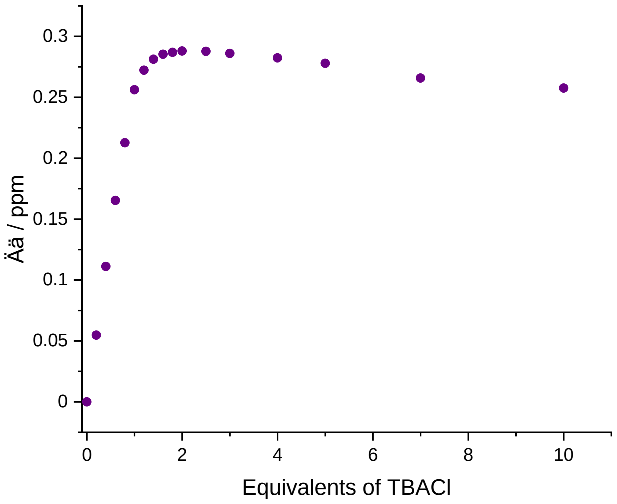

Figure S50. Anion binding isotherm for **1•XB** in acetone-d_6, circles representing experimental data

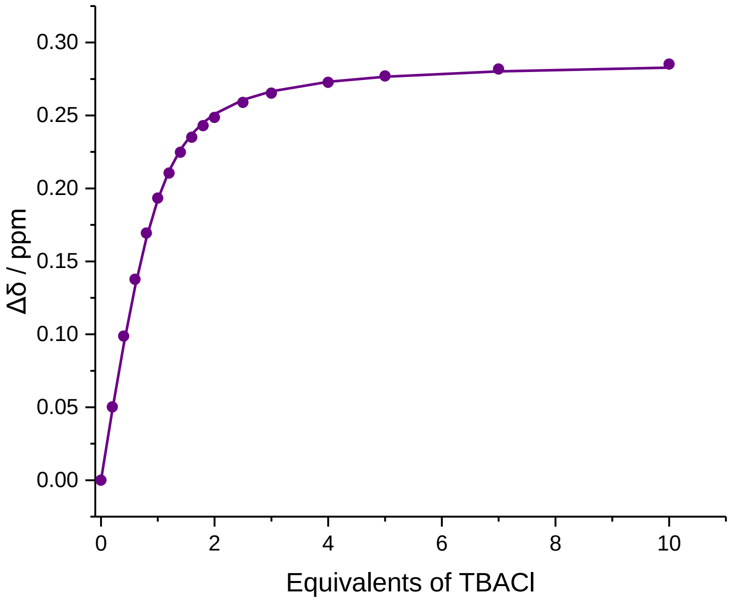

Figure S51. Anion binding isotherm for **1•XB** in 2.5% D_2O-acetone-d_6. Experimental data shown by ●, fitted 1:1 binding isotherm shown by solid line

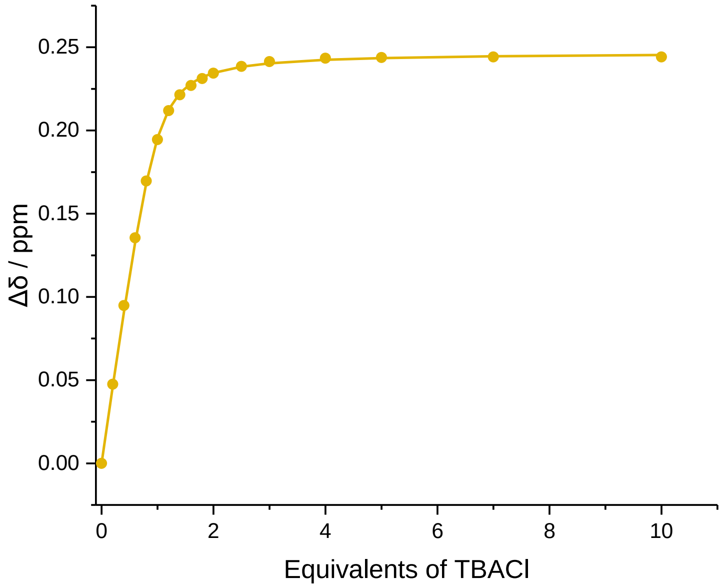

Figure S52. Anion binding isotherm for **1•ChB** in acetone-d_6. Experimental data shown by ●, fitted 1:1 binding isotherm shown by solid line

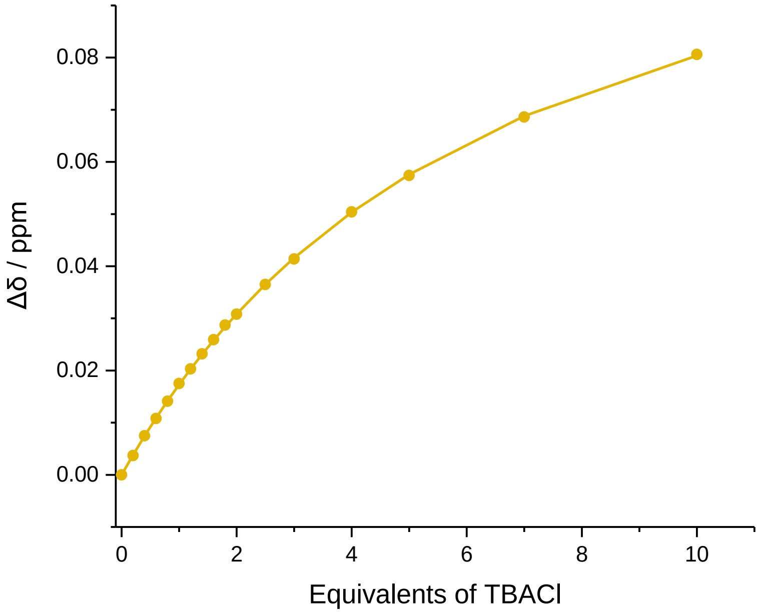

Figure S53. Anion binding isotherm for **1•ChB** in 2.5% D_2O-acetone-d_6. Experimental data shown by ●, fitted 1:1 binding isotherm shown by solid line.

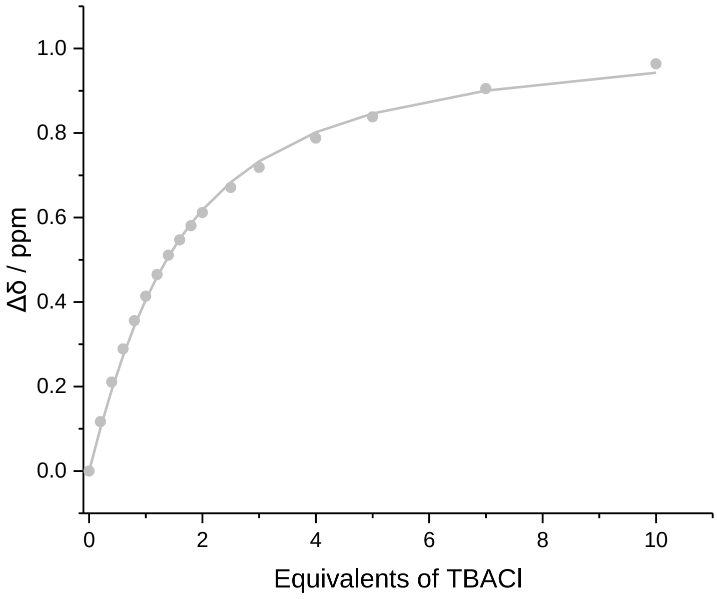

Figure S54. Anion binding isotherm for **1•HB** in acetone-d_6. Experimental data shown by ●, fitted 1:1 binding isotherm shown by solid line

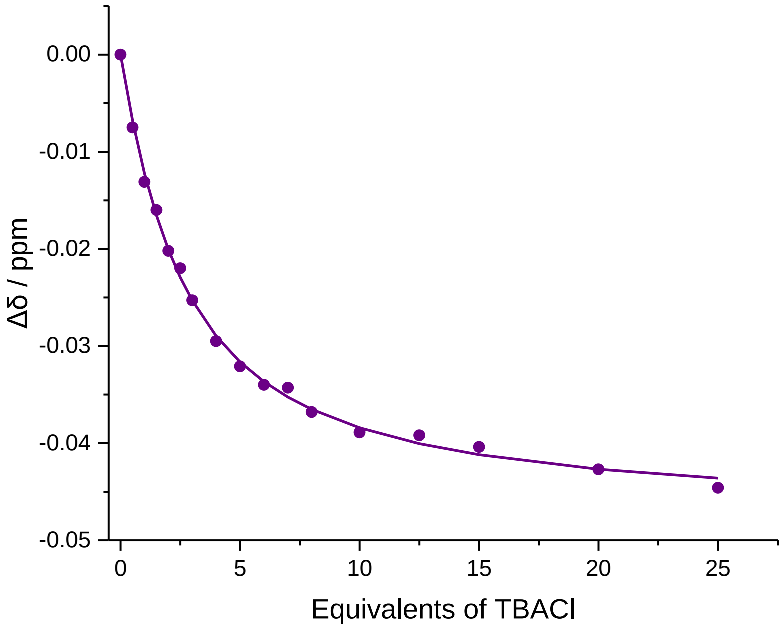

Figure S55. Anion binding isotherm for **2•XB** in acetone-d_6. Experimental data shown by ●, fitted 1:1 binding isotherm shown by solid line

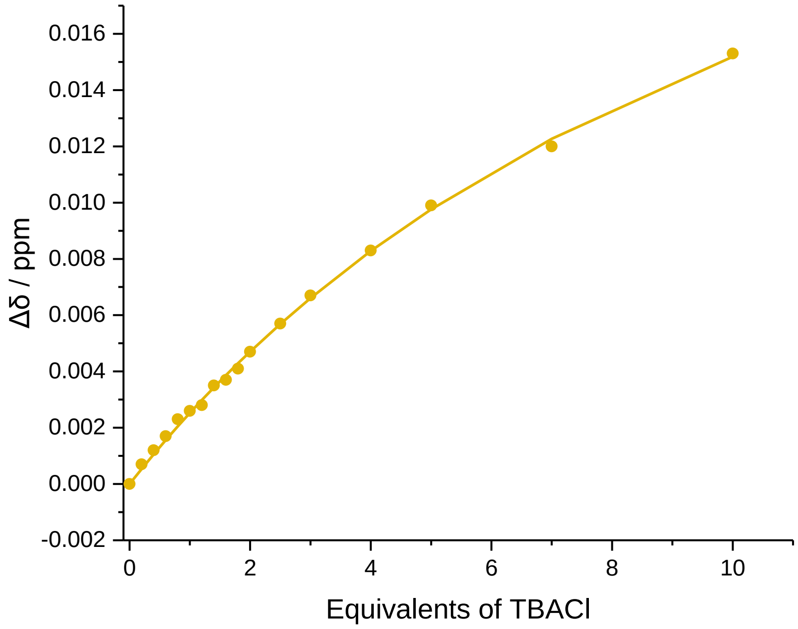

Figure S56. Anion binding isotherm for **2•ChB** in acetone-d_6. Experimental data shown by ●, fitted 1:1 binding isotherm shown by solid line

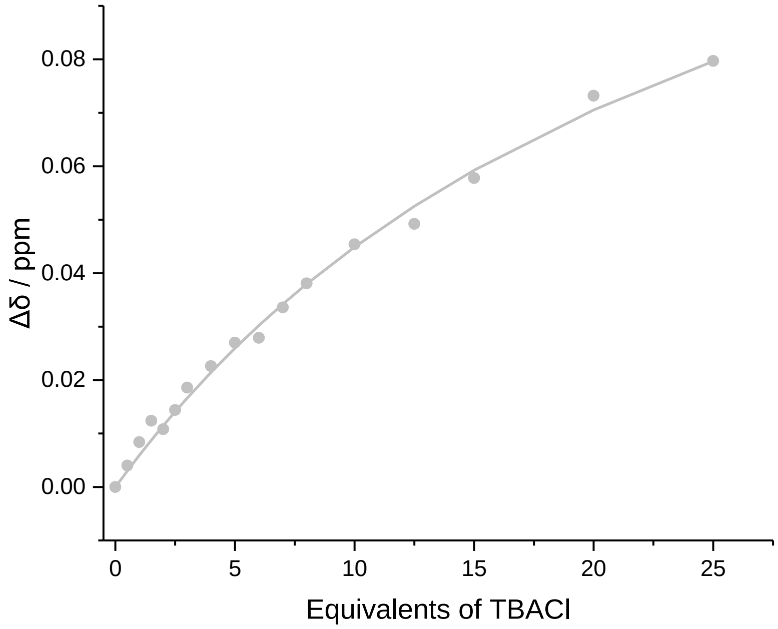

Figure S57. Anion binding isotherm for **2•HB** in acetone-d_6. Experimental data shown by ●, fitted 1:1 binding isotherm shown by solid line

6. Computational

Calculations were carried out using the ORCA suite of programs (version 4.2.1).[16] Optimisation and numerical frequency calculations were carried out at the SMD-ωB97X-D3/def2-SVP (ma-def2-SVP on anions, I and Te) level of theory (**M1**).[17–21] This choice was motivated by our previous study on halogen bonding interactions in similar systems.[22] 'Tight' convergence criteria (10^{-6} Ha on the optimisation step and 10^{-8} on the SCF energy change) were employed, and the RIJCOSX approximation was employed using an appropriate auxiliary basis set in each case.[23,24] The 'Grid6' and 'GridX6' integration grids were employed for all calculations. The def2 effective core potential (def2-ECP) was employed for 28 core electrons in calculations of iodine and tellurium.[25] Thermochemistry was calculated with the *otherm* program,[26] using Grimme's quasi-RRHO approximation (ω_0 = 45 cm^{-1}, α = 4) at a temperature of 298.15 K. A correction from 1 atm to 1 M standard state was automatically carried out in *otherm*, which adds RTln(1 mol dm^{-3} / (1/24.5 mol dm^{-3})) = 1.89 kcal mol^{-1} to the free energies of all species [$G_{\mathbf{M1},1M} = G_{\mathbf{M1},1atm}$ + RTln(24.5)]. The free energy of H_2O in implicit water was subsequently adjusted from 1 M to 55.5 M concentration by adding RTln(55.5 mol dm^{-3} / 1 mol dm^{-3}) = 2.38 kcal mol^{-1} [$G_{\mathbf{M1},55.5M}(H_2O_{H_2O}) = G_{\mathbf{M1},1M}(H_2O_{H_2O})$ + RTln(55.5)].[27] Single point energies were calculated at the SMD-ωB97X-D3/def2-TZVPP (ma-def2-TZVPP on anions, I and Te) level of theory (**M2**). Free energies at this level of theory were approximated as $G_{\mathbf{M2}} \simeq E_{\mathbf{M2}} + (G_{\mathbf{M1}} - E_{\mathbf{M1}})$. Electrostatic potential surfaces were calculated using the Gaussian 16 (rev A.03)[28] suite of programs at the SMD($CHCl_3$)-ωB97X-D/def2-TZVP level of theory, and visualised with UCSF Chimera.[29] The thermodynamic parameters used to describe anion transport are anion dehydration ($\Delta G_{dehyd} = G_{CHCl_3}(X^-) - G_{H_2O}(X^-)$) and anion binding ($\Delta G_{bind} = G_{CHCl_3}$(transporter···$X^-$ complex) – (G_{CHCl_3}(transporter) + $G_{CHCl_3}(X^-)$)). The transport process is then described by the sum of these parameters: $\Delta G_{dehyd} + \Delta G_{bind} = \Delta G_{total}$.

Transport of partially hydrated hydroxide

Bidentate binding of OH^- to **1•XB/1•ChB** leaves at least one vacant coordination site that could be occupied by a water molecule via a hydrogen bond. ΔG_{dehyd} was calculated to be 0.3 kcal mol^{-1} more favourable for $[OH\cdots H_2O]^-$ than for OH^-, which suggests that $[OH\cdots H_2O]^-$ transport is likely. Interestingly, while binding of $[OH\cdots H_2O]^-$ and OH^- to **1•XB** is similar (ΔG_{bind} = –15.1 vs –15.0 kcal mol^{-1} respectively), binding of $[OH\cdots H_2O]^-$ to **1•ChB** is weaker than for OH^- (ΔG_{bind} = –12.2 vs –14.4 kcal mol^{-1} respectively). The resulting difference in ΔG_{total} between **1•XB** and **1•ChB** increases from 0.6 kcal mol^{-1} to 3.0 kcal mol^{-1}, in better agreement with the observed Cl^-/OH^- selectivity enhancement for **1•ChB** in comparison with **1•XB**.

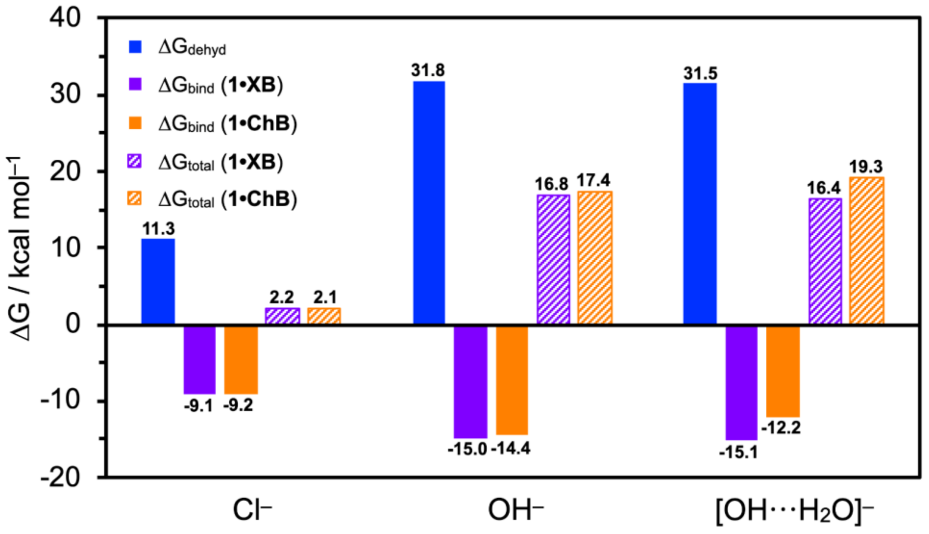

Figure S58. Calculated dehydration (ΔG_{dehyd}) and binding (ΔG_{bind}) free energies at 298.15 K for **1•XB** and **1•ChB** with Cl^-, OH^- and $[OH\cdots H_2O]^-$ at the SMD-[ωB97X-D3/def2-TZVPP (ma-def2-TZVPP on anions, I and Te)//ωB97X-D3/def2-SVP (ma-def2-SVP on anions, I and Te)] level of theory. $\Delta G_{total} = \Delta G_{dehyd} + \Delta G_{bind}$, all values in kcal mol^{-1}.

Dehydration energies of anions

Implicit solvation models are known to underestimate the solvation free energy change associated with the transfer of anions between ideal gas and aqueous phase, primarily due to the lack of short-range solvent-solute interactions (e.g. hydrogen bonds) in continuum models.[30] Therefore, it came as no surprise that using the SMD solvent model the computed desolvation energy (ΔG_{desolv}) of OH^- was underestimated by ~13 kcal mol^{-1} (91.9 kcal mol^{-1} versus the experimental value of 105.0 kcal mol^{-1}).[31] While we anticipate the error in the dehydration (i.e. H_2O to $CHCl_3$) energy considered in this study may be lower than the error in desolvation (i.e. H_2O to vacuum), a systematic underestimation of this value is likely to remain and be on the order of 10 kcal mol^{-1}. However, this error will not change the qualitative trend in ΔG_{total} observed in this study.

Table S4. Changes in electronic energy (ΔE_{el}), zero point energy (ΔZPE), enthalpy (ΔH), quasi-RRHO entropy (TΔqh-S), total thermodynamic corrections (ΔTotal corr) and quasi-RRHO free energy (Δqh-G) for the dehydration and binding processes involved in anion transport, calculated at the SMD-ωB97X-D3/def2-SVP (ma-def2-SVP on anions, I and Te) level of theory (**M1**) or [SMD-ωB97X-D3/def2-TZVPP (ma-def2-TZVPP on anions, I and Te)**//**SMD-ωB97X-D3/def2-SVP (ma-def2-SVP on anions, I and Te)] level of theory (**M2**//**M1**). Superscript terms denote the implicit solvent used in the calculation. Free energies were calculated at 298.15 K and 1M, except $H_2O_{H_2O}$ which was calculated at 55.5 M concentration and OH^{-}_{vacuum} which was calculated at 1 atm.

	**M1**						**M2//M1**	
**Process**	**ΔE_{el}**	**ΔZPE**	**ΔH**	**TΔqh-S**	**ΔTotal corr**	**Δqh-G**	**ΔE_{el}**	**Δqh-G**
$Cl^{-}_{H_2O} \longrightarrow Cl^{-}_{CHCl_3}$	11.4	0.0	11.4	0.0	0.0	11.4	11.3	11.3
$OH^{-}_{H_2O} \longrightarrow OH^{-}_{CHCl_3}$	33.0	0.0	33.0	0.0	0.0	33.0	31.8	31.8
$OH^{-}_{H_2O} \longrightarrow OH^{-}_{vacuum}$	97.0	0.0	97.0	1.9	-1.9	95.1	93.8	91.9
$OH^{-}_{H_2O} + H_2O_{H_2O} \longrightarrow [OH\cdots H2O]^{-}_{CHCl_3}$	22.6	2.3	29.3	-2.6	9.3	31.9	22.2	31.5
**1•XB**$_{H_2O} \longrightarrow$ **1•XB**$_{CHCl_3}$	-5.5	-0.1	-5.5	0.6	-0.6	-6.0	-5.8	-6.3
**1•ChB**$_{H_2O} \longrightarrow$ **1•ChB**$_{CHCl_3}$	-7.3	0.2	-7.2	-0.7	0.9	-6.5	-7.3	-6.5
**1•XB**$_{CHCl_3}$ + $Cl^{-}_{CHCl_3} \longrightarrow$ [**1•XB**···Cl]$^{-}_{CHCl_3}$	-15.3	0.3	-15.9	-6.0	5.4	-9.9	-14.5	-9.1
**1•XB**$_{CHCl_3}$ + $OH^{-}_{CHCl_3} \longrightarrow$ [**1•XB**···OH]$^{-}_{CHCl_3}$	-31.6	1.9	-24.7	-7.7	14.5	-17.1	-29.5	-15.0
**1•XB**$_{CHCl_3}$ + $[OH\cdots H_2O]^{-}_{CHCl_3} \longrightarrow$ [**1•XB**···OH···H_2O]$^{-}_{CHCl_3}$	-30.7	1.6	-29.0	-10.1	11.8	-18.9	-26.9	-15.1
**1•ChB**$_{CHCl_3}$ + $Cl^{-}_{CHCl_3} \longrightarrow$ [**1•ChB**···Cl]$^{-}_{CHCl_3}$	-14.0	-0.1	-14.9	-4.9	4.1	-9.9	-13.3	-9.2
**1•ChB**$_{CHCl_3}$ + $OH^{-}_{CHCl_3} \longrightarrow$ [**1•ChB**···OH]$^{-}_{CHCl_3}$	-29.4	1.5	-22.8	-6.7	13.3	-16.1	-27.8	-14.4
**1•ChB**$_{CHCl_3}$ + $[OH\cdots H_2O]^{-}_{CHCl_3} \longrightarrow$ [**1•ChB**···OH···H_2O]$^{-}_{CHCl_3}$	-30.1	2.1	-28.1	-10.6	12.5	-17.6	-24.7	-12.2
**1•XB**$_{CHCl_3}$ + $Cl^{-}_{H_2O} \longrightarrow$ [**1•XB**···Cl]$^{-}_{CHCl_3}$	-3.9	0.3	-4.5	-6.0	5.4	1.5	-3.2	2.2
**1•XB**$_{CHCl_3}$ + $OH^{-}_{H_2O} \longrightarrow$ [**1•XB**···OH]$^{-}_{CHCl_3}$	1.5	1.9	8.3	-7.7	14.5	16.0	2.4	16.8
**1•XB**$_{CHCl_3}$ + $OH^{-}_{H_2O} + H_2O_{H_2O} \longrightarrow$ [**1•XB**···OH···H_2O]$^{-}_{CHCl_3}$	-8.1	3.9	0.4	-12.7	21.1	13.0	-4.7	16.4
**1•ChB**$_{CHCl_3}$ + $Cl^{-}_{H_2O} \longrightarrow$ [**1•ChB**···Cl]$^{-}_{CHCl_3}$	-2.6	-0.1	-3.4	-4.9	4.1	1.5	-2.0	2.1
**1•ChB**$_{CHCl_3}$ + $OH^{-}_{H_2O} \longrightarrow$ [**1•ChB**···OH]$^{-}_{CHCl_3}$	3.7	1.5	10.3	-6.7	13.3	17.0	4.1	17.4
**1•ChB**$_{CHCl_3}$ + $OH^{-}_{H_2O} + H_2O_{H_2O} \longrightarrow$ [**1•ChB**···OH···H_2O]$^{-}_{CHCl_3}$	-7.5	4.3	1.2	-13.2	21.8	14.4	-2.5	19.4

Calculated transporter geometries and complexes

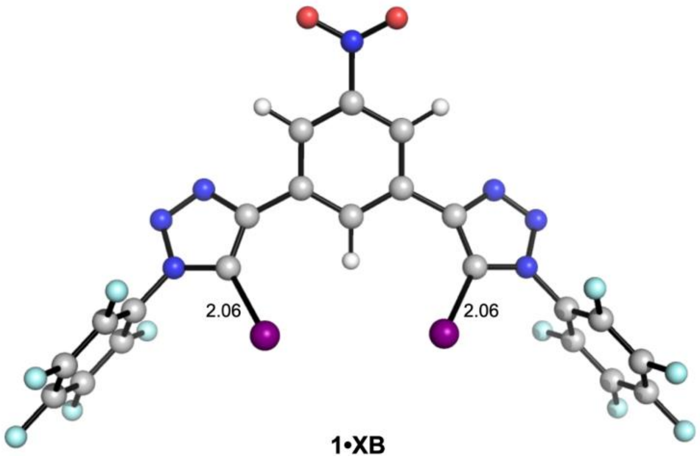

Figure S59. Calculated geometry of **1•XB** at the SMD($CHCl_3$)-ωB97X-D3/def2-SVP (ma-def2-SVP on I) level of theory**.** Key distances are shown in Å.

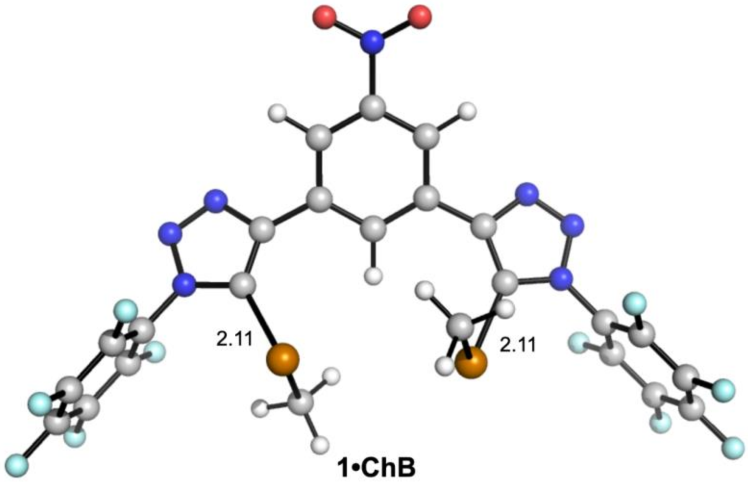

Figure S60. Calculated geometry of **1•ChB** at the SMD($CHCl_3$)-ωB97X-D3/def2-SVP (ma-def2-SVP on I) level of theory**.**

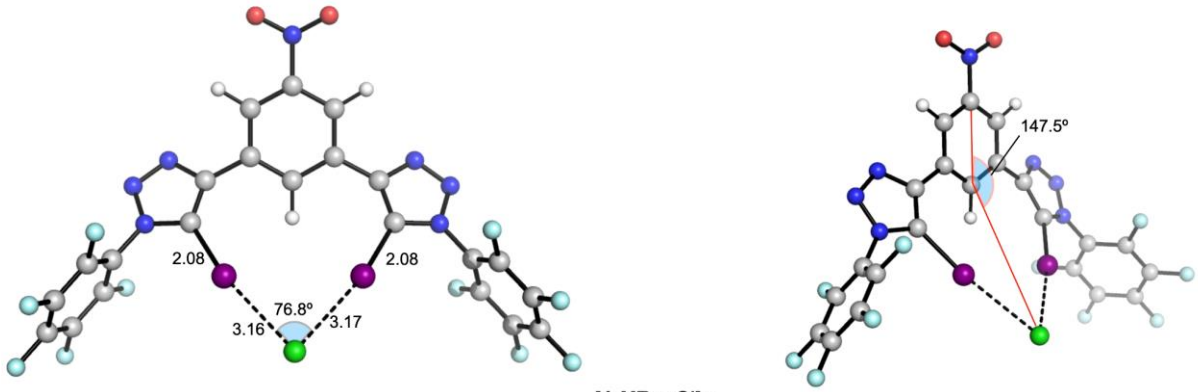

Figure S61. Calculated geometry of [**1•XB**···Cl]$^-$ at the SMD($CHCl_3$)-ωB97X-D3/def2-SVP (ma-def2-SVP on I) level of theory**.** Key distances are shown in Å, and angles in degrees.

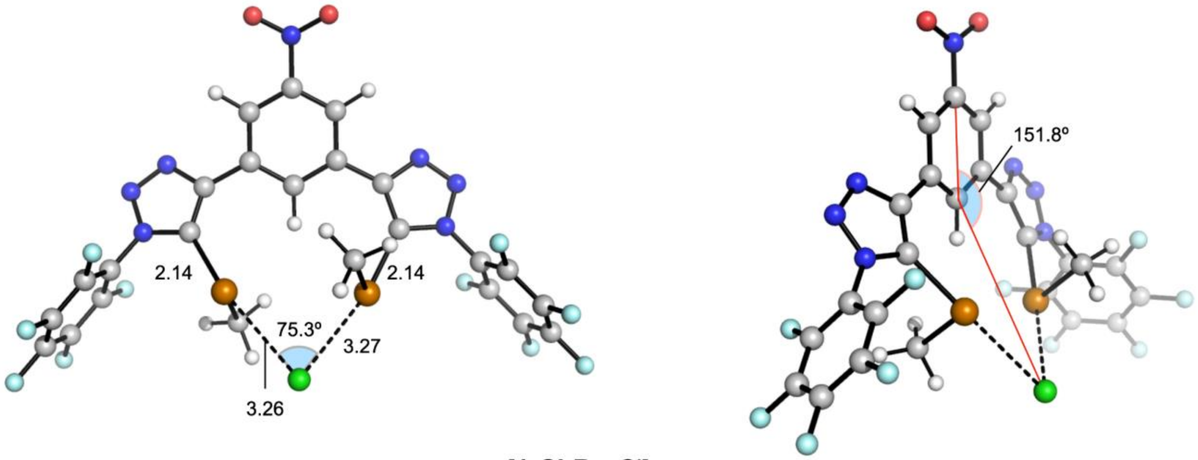

[**1•ChB**···Cl]–

Figure S62. Calculated geometry of [**1•ChB**···Cl]– at the SMD($CHCl_3$)-ωB97X-D3/def2-SVP (ma-def2-SVP on I) level of theory**.** Key distances are shown in Å, and angles in degrees.

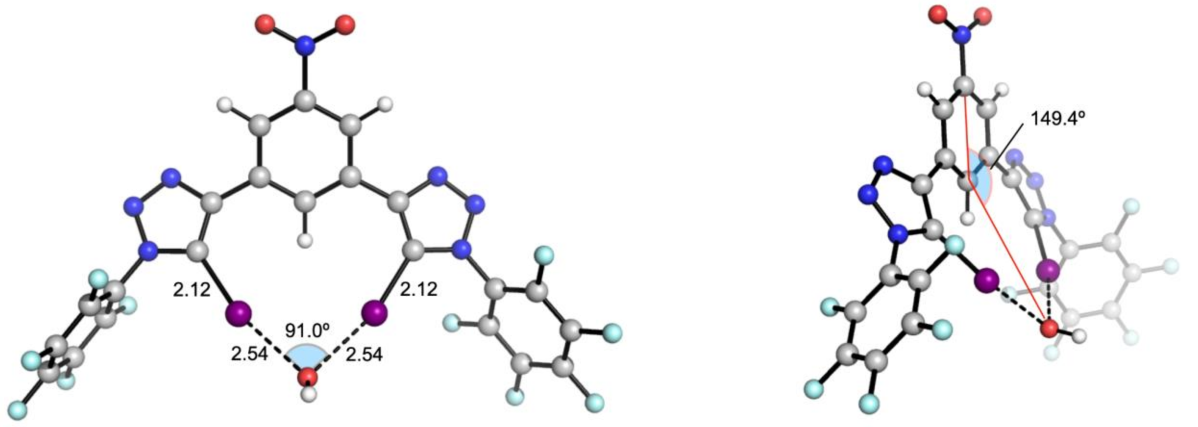

[**1•XB**···OH]–

Figure S63. Calculated geometry of [**1•XB**···OH]– at the SMD($CHCl_3$)-ωB97X-D3/def2-SVP (ma-def2-SVP on I) level of theory**.** Key distances are shown in Å, and angles in degrees.

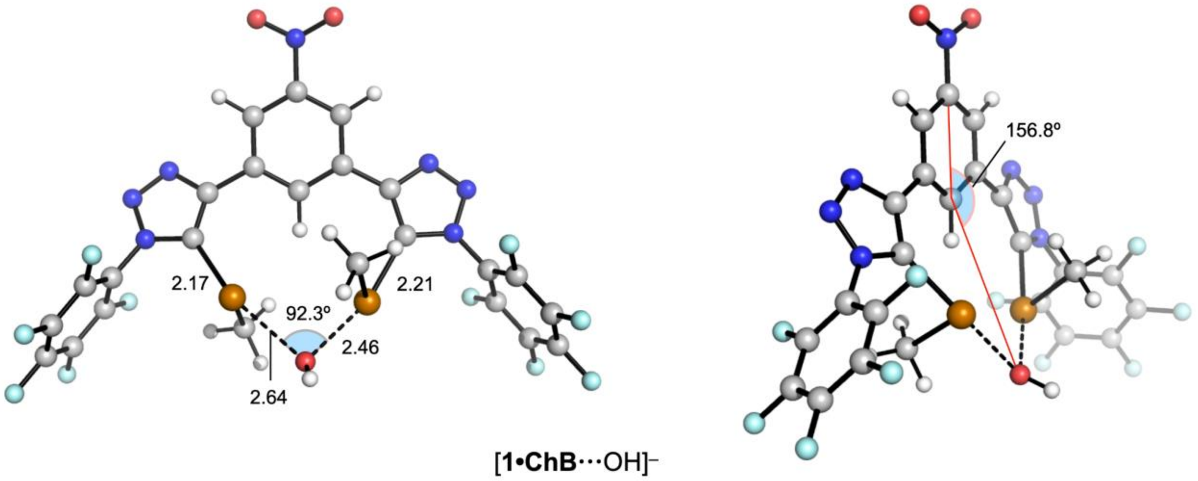

[**1•ChB**···OH]–

Figure S64. Calculated geometry of [**1•ChB**···OH]– at the SMD($CHCl_3$)-ωB97X-D3/def2-SVP (ma-def2-SVP on I) level of theory**.** Key distances are shown in Å, and angles in degrees.

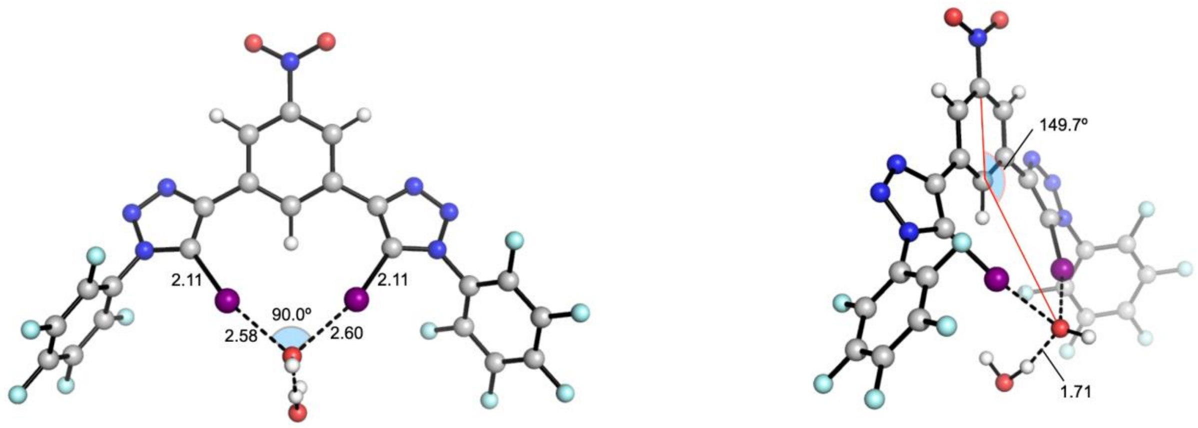

[**1•XB**···OH···H_2O]$^-$

Figure S65. Calculated geometry of [**1•XB···**OH···H_2O]$^-$ at the SMD($CHCl_3$)-ωB97X-D3/def2-SVP (ma-def2-SVP on I) level of theory**.** Key distances are shown in Å, and angles in degrees.

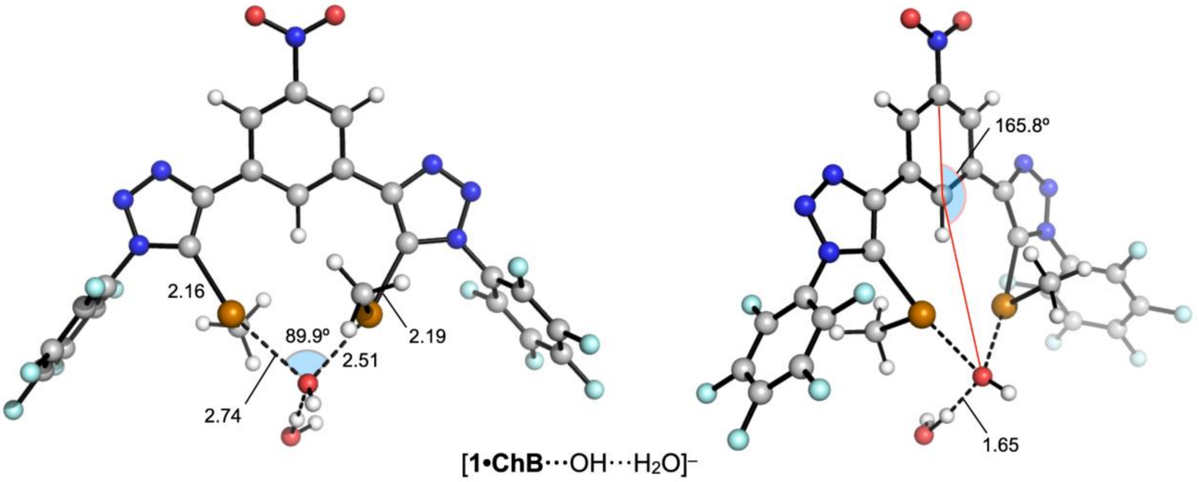

[**1•ChB**···OH···H_2O]$^-$

Figure S66. Calculated geometry of [**1•ChB**···OH···H_2O]$^-$ at the SMD($CHCl_3$)-ωB97X-D3/def2-SVP (ma-def2-SVP on I) level of theory**.** Key distances are shown in Å, and angles in degrees.

Table S5. Electronic energy (E_{el}), zero point energy (ZPE), enthalpy (H), quasi-RRHO entropy (Tqh-S), total thermodynamic corrections (Total corr) and quasi-RRHO free energy (qh-G) for the dehydration and binding processes involved in anion transport, calculated at the SMD-ωB97X-D3/def2-SVP (ma-def2-SVP on anions, I and Te) level of theory (**M1**) or [SMD-ωB97X-D3/def2-TZVPP (ma-def2-TZVPP on anions, I and Te)**//**SMD-ωB97X-D3/def2-SVP (ma-def2-SVP on anions, I and Te)] level of theory (**M2**//**M1**). Superscript terms denote the implicit solvent used in the calculation. Free energies were calculated at 298.15 K and 1M, except $H_2O_{H_2O}$ which was calculated at 55.5 M concentration, and OH^-_{vacuum} which was calculated at 1 atm.

	**M1**						**M2//M1**	
**Species**	E_{el}	**ZPE**	**H**	**Tqh-S**	**Total corr**	**qh-G**	E_{el}	**qh-G**
$Cl^-_{CHCl_3}$	-460.22041	0.00001	-460.21663	0.01438	-0.01060	-460.23101	-460.36747	-460.37807
$OH^-_{CHCl_3}$	-75.81959	0.00885	-75.81629	0.01593	-0.01263	-75.83222	-75.90735	-75.91998
$[OH\cdots H_2O]^-_{CHCl_3}$	-152.18628	0.03394	-152.14704	0.02646	0.01278	-152.17350	-152.37759	-152.36481
**1•XB**$_{CHCl_3}$	-2965.14643	0.24590	-2964.86350	0.10215	0.18077	-2964.96566	-2967.88550	-2967.70473
[**1•XB**···Cl]$^-_{CHCl_3}$	-3425.39120	0.24636	-3425.10547	0.10698	0.17874	-3425.21246	-3428.27609	-3428.09734
[**1•XB**···OH]$^-_{CHCl_3}$	-3041.01630	0.25786	-3040.71920	0.10588	0.19121	-3040.82509	-3043.83981	-3043.64860
[**1•XB**···OH···H_2O]$^-_{CHCl_3}$	-3117.38163	0.28242	-3117.05668	0.11257	0.21237	-3117.16926	-3120.30597	-3120.09360
**1•ChB**$_{CHCl_3}$	-2985.55474	0.32046	-2985.19276	0.11043	0.25155	-2985.30319	-2988.38752	-2988.13598
[**1•ChB**···Cl]$^-_{CHCl_3}$	-3445.79750	0.32032	-3445.43307	0.11692	0.24751	-3445.54999	-3448.77616	-3448.52865
[**1•ChB**···OH]$^-_{CHCl_3}$	-3061.42115	0.33168	-3061.04533	0.11568	0.26014	-3061.16101	-3064.33910	-3064.07896
[**1•ChB**···OH···H_2O]$^-_{CHCl_3}$	-3137.78897	0.35772	-3137.38465	0.12006	0.28426	-3137.50471	-3140.80442	-3140.52016
$Cl^-_{H_2O}$	-460.23863	0.00000	-460.23485	0.01438	-0.01060	-460.24923	-460.38546	-460.39606
$OH^-_{H_2O}$	-75.87225	0.00888	-75.86894	0.01593	-0.01263	-75.88488	-75.95806	-75.97069
$H_2O_{H_2O}$	-76.35005	0.02147	-76.32480	0.01471	0.01053	-76.33951	-76.45488	-76.44435
**1•XB**$_{H_2O}$	-2965.13769	0.24605	-2964.85477	0.10126	0.18165	-2964.95603	-2967.87633	-2967.69468
**1•ChB**$_{H_2O}$	-2985.54304	0.32010	-2985.18127	0.11159	0.25018	-2985.29286	-2988.37585	-2988.12567
OH^-_{vacuum}	-75.71772	0.00880	-75.71442	0.01895	-0.01565	-75.73337	-75.80852	-75.82417

7. References

[1] C. J. Brassard, X. Zhang, C. R. Brewer, P. Liu, R. J. Clark, L. Zhu, *J. Org. Chem.* **2016**, *81*, 12091–12105.

[2] L.-M. Jin, X. Xu, H. Lu, X. Cui, L. Wojtas, X. P. Zhang, *Angew. Chem. Int. Ed.* **2013**, *52*, 5309–5313.

[3] J. Charoenpattarapreeda, Y. S. Tan, J. Iegre, S. J. Walsh, E. Fowler, R. S. Eapen, Y. Wu, H. F. Sore, C. S. Verma, L. Itzhaki, D. R. Spring, *Chem. Commun.* **2019**, *55*, 7914–7917.

[4] A. Docker, T. Bunchuay, M. Ahrens, A. J. Martinez-Martinez, P. D. Beer, *Chem. Eur. J.* **2021**, DOI:10.1002/chem.202100579.

[5] S.-N. Chen, T.-T. Hung, T.-C. Lin, F.-Y. Tsai, *J. Chin. Chem. Soc.* **2009**, *56*, 1078–1081.

[6] J. Cosier, A. M. Glazer, *J. Appl. Cryst.* **1986**, *19*, 105–107.

[7] *CrysAlisPRO*, Oxford Diffraction /Agilent Technologies UK Ltd, Yarnton, England., **n.d.**

[8] L. Palatinus, G. Chapuis, *J. Appl. Cryst.* **2007**, *40*, 786–790.

[9] P. W. Betteridge, J. R. Carruthers, R. I. Cooper, K. Prout, D. J. Watkin, *J. Appl. Cryst.* **2003**, *36*, 1487–1487.

[10] A. V. Jentzsch, D. Emery, J. Mareda, S. K. Nayak, P. Metrangolo, G. Resnati, N. Sakai, S. Matile, *Nat. Commun.* **2012**, *3*, 905.

[11] N. Busschaert, R. B. P. Elmes, D. D. Czech, X. Wu, I. L. Kirby, E. M. Peck, K. D. Hendzel, S. K. Shaw, B. Chan, B. D. Smith, K. A. Jolliffe, P. A. Gale, *Chem. Sci.* **2014**, *5*, 3617–3626.

[12] X. Wu, L. W. Judd, E. N. W. Howe, A. M. Withecombe, V. Soto-Cerrato, H. Li, N. Busschaert, H. Valkenier, R. Pérez-Tomás, D. N. Sheppard, Y.-B. Jiang, A. P. Davis, P. A. Gale, *Chem* **2016**, *1*, 127–146.

[13] R. E. Dawson, A. Hennig, D. P. Weimann, D. Emery, V. Ravikumar, J. Montenegro, T. Takeuchi, S. Gabutti, M. Mayor, J. Mareda, C. A. Schalley, S. Matile, *Nat. Chem.* **2010**, *2*, 533–538.

[14] “BindFit v0.5 can be found under http://app.supramolecular.org/bindfit/

[15] D. Brynn Hibbert, P. Thordarson, *Chem. Commun.* **2016**, *52*, 12792–12805.

[16] F. Neese, *Wiley Interdiscip. Rev. Comput. Mol. Sc.* **2018**, *8*, 4–9.

[17] S. Kozuch, J. M. L. Martin, *J. Chem. Theory Comput.* **2013**, *9*, 1918–1931.

[18] L. N. Anderson, F. W. Aquino, A. E. Raeber, X. Chen, B. M. Wong, *J. Chem. Theory Comput.* **2018**, *14*, 180–190.

[19] Y.-S. Lin, G.-D. Li, S.-P. Mao, J.-D. Chai, *J. Chem. Theory Comput.* **2013**, *9*, 263–272.

[20] A. V. Marenich, C. J. Cramer, D. G. Truhlar, *J. Phys. Chem. B* **2009**, *113*, 6378–6396.

[21] F. Weigend, R. Ahlrichs, *Phys. Chem. Chem. Phys.* **2005**, *7*, 3297–3305.

[22] L. E. Bickerton, A. J. Sterling, P. D. Beer, F. Duarte, M. J. Langton, *Chem. Sci.* **2020**, *11*, 4722–4729.

[23] F. Neese, F. Wennmohs, A. Hansen, U. Becker, *Chem. Phys.* **2009**, *356*, 98–109.

[24] F. Weigend, *Phys. Chem. Chem. Phys.* **2006**, *8*, 1057–1065.

[25] K. A. Peterson, *J. Chem. Phys.* **2003**, *119*, 11099–11112.

[26] Tom Young, *Duartegroup/Otherm: Major Symmetry Improvements*, Zenodo, **2020**. DOI: 10.5281/zenodo.3294010

[27] S. Grimme, *Chem. Eur. J.* **2012**, *18*, 9955–9964.

[28] M. J. Frisch, G. W. Trucks, H. B. Schlegel, G. E. Scuseria, M. A. Robb, J. R. Cheeseman, G. Scalmani, V. Barone, G. A. Petersson, X. L. H. Nakatsuji, M. Caricato, A. Marenich, J. Bloino, B. G. Janesko, R. Gomperts, B. Mennucci, H. P. Hratchian, J. V. Ortiz, A. F. Izmaylov, J. L. Sonnenberg, D. Williams-Young, F. Ding, F. Lipparini, F. Egidi, J. Goings, B. Peng, A. Petrone, T. Henderson, D. Ranasinghe, V. G. Zakrzewski, J. Gao, N. Rega, G. Zheng, W. Liang, M. Hada, M. Ehara, K. Toyota, R. Fukuda, J. Hasegawa, M. Ishida, T. Nakajima, Y. Honda, O. Kitao, H. Nakai, T. Vreven, K. Throssell, J. A. J. Montgomery, J. E. Peralta, F. Ogliaro, M. Bearpark, J. J. Heyd, E. Brothers, K. N. Kudin, V. N. Staroverov, T. Keith, R. Kobayashi, J. Normand, K. Raghavachari, A. Rendell, J. C. Burant, S. S. Iyengar, J. Tomasi, M. Cossi, J. M. M. Millam, M. Klene, C. Adamo, R.

Cammi, J. W. Ochterski, R. L. Martin, K. Morokuma, O. Farkas, J. B. Foresman and D. J. Fox, **2016**, Gaussian 16, Revision A.03.
[29] E. F. Pettersen, T. D. Goddard, C. C. Huang, G. S. Couch, D. M. Greenblatt, E. C. Meng, T. E. Ferrin, *J. Comput.l Chem.* **2004**, *25*, 1605–1612.
[30] Y. Takano, K. N. Houk, *J. Chem. Theory Comput.* **2005**, *1*, 70–77.
[31] J. R. P. Jr, J. M. Riveros, *Phys. Chem. Chem. Phys.* **2002**, *4*, 1622–1627.
